# Enzymatic aminolysis in continuous flow: an efficient strategy for the synthesis of salicylamide derivatives

**DOI:** 10.1039/d5ra09739h

**Published:** 2026-03-02

**Authors:** Li-Hua Du, Miao-Miao Xue, Bing-Lin Yan, Lin Wang, Xi-Ping Luo

**Affiliations:** a College of Pharmaceutical Science, ZheJiang University of Technology Hangzhou 310014 Zhejiang China orgdlh@zjut.edu.cn +86 571 88320903 +86-189-690-693-99; b Zhejiang Provincial Key Laboratory of Chemical Utilization of Forestry Biomass, Zhejiang A&F University Hangzhou 311300 Zhejiang China

## Abstract

Salicylamide derivatives with different amine substitutions demonstrate potent biological activities such as anti-tumor, antibacterial and neuroprotective properties. In this work, a green and efficient strategy for the synthesis of salicylamide derivatives catalyzed by Lipozyme®TL IM from *Thermomyces lanuginosus* in continuous flow microreactors was developed. Enzymatic aminolysis from methyl salicylate and amines (cyclic and linear aliphatic amines, aryl alkyl amines, tryptamines) in a continuous-flow microreactor was used for the synthesis of salicylamide derivatives. Reaction parameters were optimized, and continuous flow was compared with conventional batch methods using space-time yield (STY). This strategy showed broad substrate applicability, enabling the synthesis of 34 salicylamide derivatives with ideal yields. It provides a novel sustainable approach for precise modification of the salicylamide skeleton, offering valuable compounds for drug screening and structure–activity relationship studies.

## Abstract 2

Reaction parameters were optimized in a K_2_CO_3_/Lipozyme® TL IM catalyzed continuous-flow microreactor. An efficient biocatalytic strategy afforded 34 salicylamide derivatives. This work supports their synthesis and industrialization.

## Graphical abstract

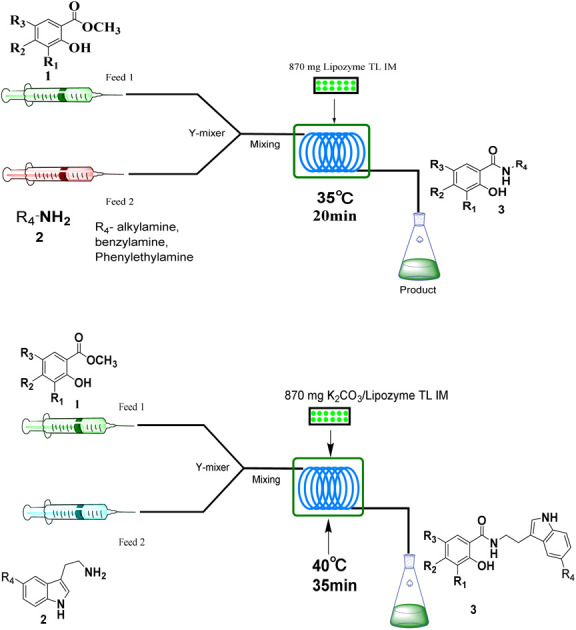

## Introduction

**Fig. 1:**
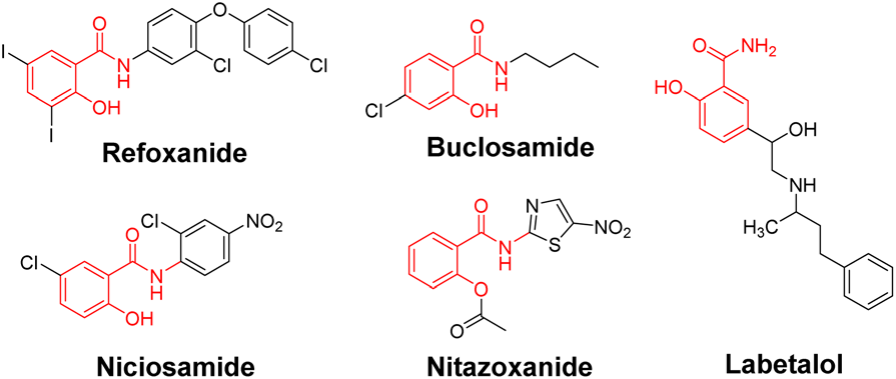
Structures of salicylamide derivatives.

Salicylamide derivatives possess biological activities such as anti-tumor, antibacterial and neuroprotective properties,^1–3^ which can be applied to Alzheimer's disease,^4^ Parkinson's disease,^5^ depression,^6^ viral infections,^7^ hypertension,^8^ and parasitic infections.^9^ Numerous drugs containing the salicylamide structure have been widely used in clinical practice,^10–14^ like rafoxanide (antiparasitic activity), niclosamide (STAT3 inhibitory activity), nitazoxanide (antiviral activity), and labetalol (antihypertensive activity) (Fig. 1). Salicylic acid serves as a fundamental pharmacophore, conferring anti-inflammatory, analgesic, antioxidant, and metal-chelating properties; the introduction of amines with different structures further demonstrates a variety of biological activities on the basis of this activity.^15^ Salicylanilides synthesized by coupling with aniline are renowned for their antibacterial, antiviral and antitumor activities,^16^ and conjugation with tryptamine generates *N*-salicyltryptamine derivatives, providing new insight for designing multi-functional candidates for neuroinflammation related neurodegenerative diseases.^17^ Salicylamide derivatives produced by the reaction with heterocyclic amines have antibacterial, antioxidant, enzyme inhibitory and other activities,^18,19^ while salicylamide derivatives with various amino acid linkers have yielded compounds with diverse biological profiles, such as anticancer activity, inhibition of *Pseudomonas aeruginosa*, and anti-human adenovirus (HAdV) effects.^20^

**Fig. 2:**
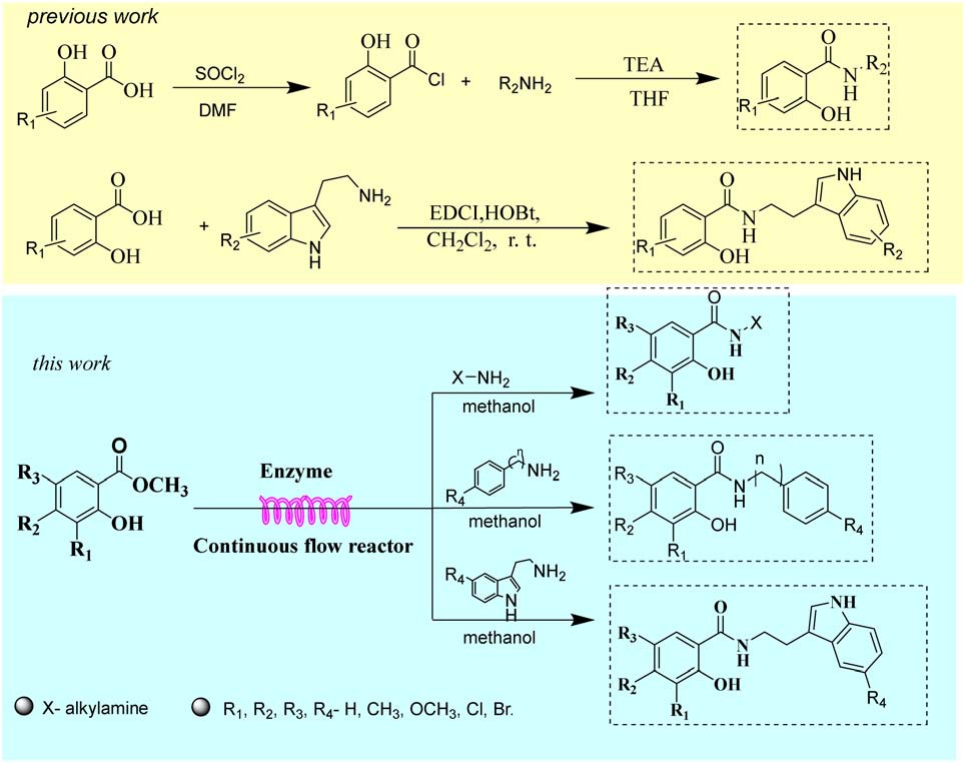
Synthetic route of salicylamide derivatives.

Construction of amide bonds is the core step in the synthesis of salicylamide derivatives, and the structure and formation efficiency of amide bonds determine the structural diversity and biological activities. Among the synthetic methodologies reported for salicylamide derivatives, the two-step acyl chloride-mediated route remains the widely adopted strategy in both laboratory research and industrial production. Salicylic acid activates *via* acylation with thionyl chloride to form salicylyl chloride, which then reacts with corresponding amine substrates through nucleophilic substitution to produce salicylamide derivatives.^21–24^ This method requires the separation and purification of salicyloyl chloride, and the acyl chlorination reaction must be carried out under anhydrous conditions. Another viable route to obtain salicylamide derivatives is the condensation of salicylic acid and amines, such as using catalysts like EDCI/HOBt, EDCI/DMAP, or transition metal catalysts (*e.g.*, copper or palladium).^25–29^ The treatment of the EDCI/HOBt and EDCI/DMAP catalytic systems increases the purification cost, the leaching of heavy metal ions from transition metal catalysts into products impacts product purity and environmental pollution, and the cost of palladium catalysts restricts industrial application. The synthetic strategy of salicylamide derivatives is shown in Fig. 2.

Biocatalysis has found widespread application in pharmaceuticals,^30^ agrochemicals,^31^ and fine chemicals.^32,33^ An industrial panel of synthetic chemists has identified biocatalysis as one of the most promising approaches for developing sustainable chemical alternatives.^34–38^ While lipases are well-known for their high efficiency in hydrolysis and esterification reactions, several studies have confirmed their substantial potential to catalyze amide bond formation *via* aminolysis or reverse hydrolysis pathways.^39,40^ Mycelial lipases from *Rhizopus arrhizus* and *Penicillium cyclopium* have been employed for the aminolysis reaction of methyl salicylate with primary amines, affording salicylamide derivatives in 40–60% yields.^41^ However, these mycelial lipases suffer from several limitations in practical application: their catalytic efficiency is relatively low, affording moderate yields; they are directly used as catalysts in the form of mycelia without immobilization, which may lead to poor stability and difficulty in recycling, increasing the cost of continuous reaction; additionally, their substrate scope is narrow, with no catalytic activity toward secondary amines and biogenic amines, thus limiting the synthetic scope of salicylamide derivatives. After trying the above two lipases, immobilized *Candida Antarctica* Lipase B, Lipozyme®TL IM, and Lipura®Flex for the synthesis of salicylamide derivatives, we found that the catalytic effect of Lipozyme®TL IM was better. Therefore, we propose a one-step strategy for the synthesis of salicylamide derivatives catalyzed by Lipozyme® TL IM, an immobilized lipase derived from *Thermomyces lanuginosus*. Lipozyme®TL IM has demonstrated effective application in the amidation reaction, such as the catalyzed synthesis of *trans*-ferulic acid tyramine (Barsi *et al.*), a process that achieved the yield of 96% after 52 h.^42^ Despite the application of Lipozyme®TL IM in amidation reactions, there are relatively few studies on the synthesis of salicylamide derivatives catalyzed by Lipozyme®TL IM. Although enzymatic reactions proceed under mild conditions, they are often associated with extended reaction times (24 h or more). To address this challenge, we propose to enhance the process using continuous-flow technology, which has gained significant attention in recent years for its potential in enzymatic reactions.

**Scheme 1:**
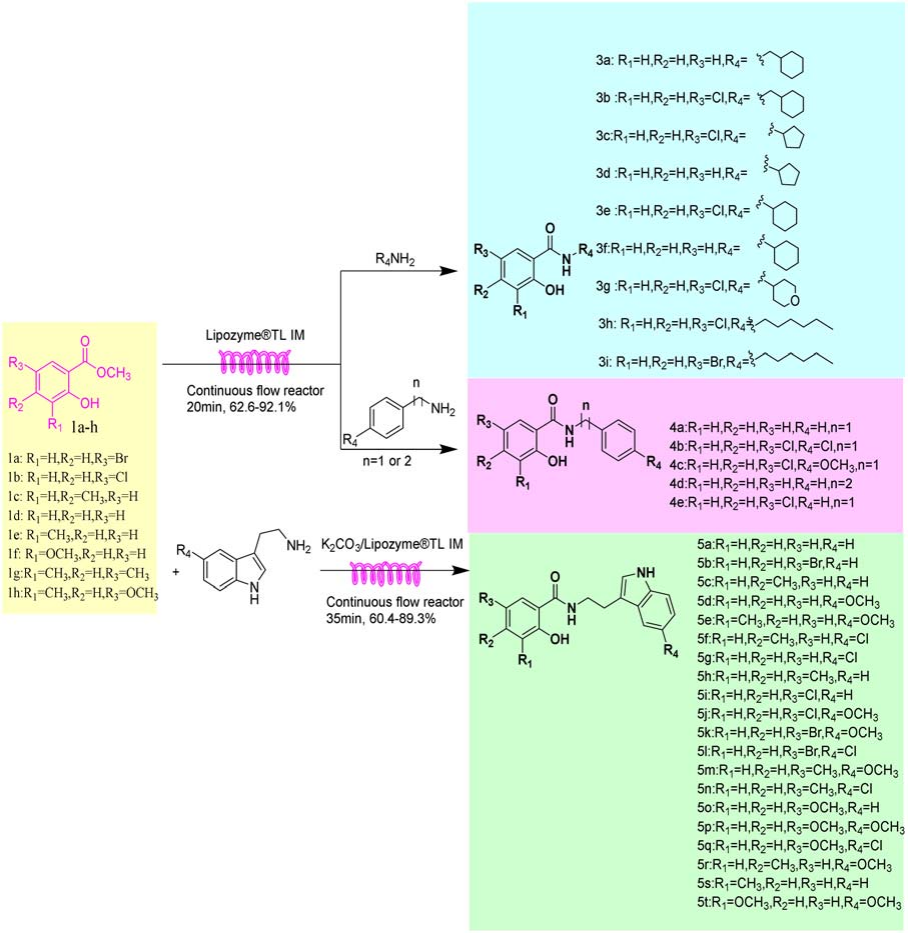
Synthesis of salicylamide derivatives in the continuous flow microreactors.

Continuous-flow microreactor technology acts as a core driver of innovation across pharmaceuticals, fine chemicals, and many other industries.^43–45^ The confined microchannels in such reactors enhance mass and heat transfer, ensuring highly efficient and controllable reactions.^46,47^ The improved mass/heat transfer capabilities of flow systems not only shorten processing times, thereby opening new avenues for the scalable and economical application of technologies like enzyme catalysis,^48,49^ but also boost reaction selectivity and simplify downstream purification.^50,51^ Herein, we try to study a method of combining continuous flow technology and biocatalysis technology to apply to the synthesis of salicylamide derivatives. This technology is researched from salicylate esters and various types of amines (cyclic aliphatic amines and linear aliphatic amines, arylalkyl amines, and tryptamines) as starting materials. Reaction parameters, such as enzyme type, solvent, substrate ratio, reaction temperature, residence time, and mixing ratio of K_2_CO_3_/Lipozyme®TL IM were systematically examined. The influence of reaction substrate structure and electronic effect on reaction yield has also been analyzed and studied. A comparative study was conducted on the synthesis of salicylamide derivatives by continuous-flow and batch operations. Under the same parameters, continuous flow microreactors and shaker reactors were respectively used for reactions to fairly compare and determine the yield, and their production efficiency was quantitatively evaluated through spatio-temporal yield (STY). Finally, we conducted a study on the substrate applicability and found that this technology has a wide range of substrate applicability, which can catalyze salicyl esters and different types of amines to carry out enzymatic aminolysis, and synthesize 34 different types of salicylamide derivatives in one step (Scheme 1). All products were isolated, purified, and structurally characterized.

## Results and discussion

### Effect of reaction solvent and enzyme

Methyl salicylate and cyclohexanemethylamine were chosen as model substrates to investigate the synthesis of salicylamide derivatives in a continuous-flow microreactor. The reaction solvent has different effects on the activity of the enzyme and the reaction rate. To determine the optimal reaction medium, we made active attempts with many solvents, such as methanol, ethanol, isopropyl alcohol, *tert*-amyl alcohol, acetonitrile, acetone, *n*-hexane, DMF, cyclohexane and DMSO. The experimental results are shown in Table 1, methanol and *tert*-amyl alcohol effectively promote the reaction, methanol was selected as the reaction solvent due to its cost-effectiveness and ease of recovery. In addition, methanol has a moderate polarity (log *P* ≈ −0.77), which not only ensures the solubility of the polar substrate methyl salicylate but also maintains the hydrated state of the enzyme-preserving the necessary water layer around the enzyme active site to avoid enzyme inactivation. The intrinsic selectivity and activity of an enzyme, which are influenced by its unique three-dimensional structure, influence the reaction's efficiency. The yields of using Lipozyme®TL IM, CAL-B, Lipura®Flex, and Lipozyme®RM IM were compared under the same reaction conditions. Lipozyme® TL IM exhibited the highest catalytic efficiency, with a yield of 81.3%, and thus was ultimately selected as the catalyst.

**Table 1:** The effect of reaction solvent and enzyme type on the synthesis of salicylamide derivatives in continuous-flow microreactors^a^

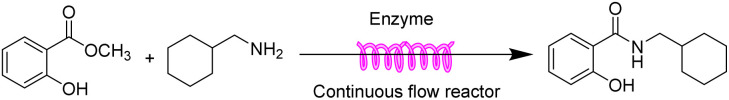			
Entry	Solvent	Catalysts	Yield^b^ (%)
1	Methanol	None	n.d.
2	**Methanol**	Lipozyme®TL IM	81.3 ± 0.7%
3	DMSO	Lipozyme®TL IM	71.6 ± 0.6%
4	Acetone	Lipozyme®TL IM	47.3 ± 1.1%
5	Cyclohexane	Lipozyme®TL IM	32.1 ± 0.9%
6	Ethanol	Lipozyme®TL IM	58.6 ± 0.4%
7	Isopropyl alcohol	Lipozyme®TL IM	68.9 ± 0.8%
8	*n*-Hexane	Lipozyme®TL IM	19.4 ± 0.7%
9	Acetonitrile	Lipozyme®TL IM	43.1 ± 0.5%
10	*tert*-Amyl alcohol	Lipozyme®TL IM	80.7 ± 0.9%
11	DMF	Lipozyme®TL IM	63.9 ± 0.3%
12	Methanol	CAL-B	80.3 ± 1.3%
13	Methanol	Lipura®Flex	67.1 ± 1.1%
14	Methanol	Lipozyme®RM IM	69.3 ± 0.8%

a General experimental conditions: in the continuous flow reactors, feed 1, 5.0 mmol methyl salicylate was taken and added to solvent to prepare 10 mL of solution; feed 2, 10 mL solvent contained 10.0 mmol cyclohexanemethylamine, 35 °C, flow rate 31.2 µL min^−1^ residence time 20 min, enzyme 870 mg.

b Isolated yield. Yield: 100× (actual received amount/ideal calculated amount). The data are presented as average ± standard deviation (SD) of triplicate experiments. n. d. means no reaction was found.

### Effect of substrate ratio

**Fig. 3:**
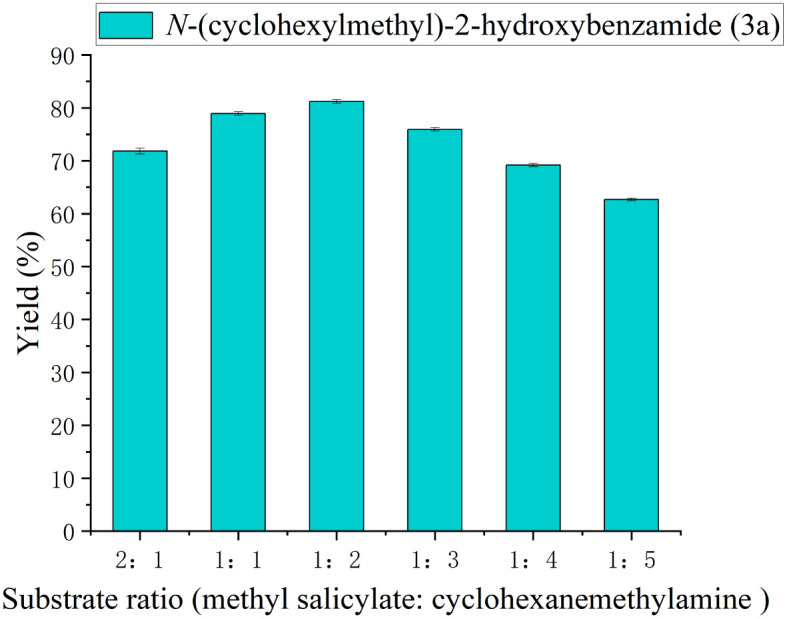
The effect of substrate ratio (methyl salicylate : cyclohexanemethylamine) on the enzymatic synthesis of salicylamide derivatives in continuous-flow microreactors.

Achieving an optimal balance in substrate ratio is crucial for maximizing product yield and minimizing the formation of wasteful by-products. To maximize the conversion rate of methyl salicylate and suppress possible side reactions, we investigated the influence of the molar ratio of amines to methyl salicylate on the reaction. As shown in Fig. 3, the substrate molar ratio of methyl salicylate: cyclohexanemethylamine (2 : 1, 1 : 1, 1 : 2, 1 : 3, 1 : 4, 1 : 5) was investigated. As the ratio reached 1 : 2, the yield of salicylamide derivatives improved, indicating that the slightly excessive amine effectively promoted the reaction equilibrium. However, when the molar ratio was further increased to 1 : 3, the yield did not continue to rise and even a slight decline was observed. This might be due to the fact that the high concentration of amines caused a certain degree of substrate inhibition on Lipozyme® TL IM. Therefore, a molar ratio of 1 : 2 was selected for the further study.

### Effect of reaction temperature

**Fig. 4:**
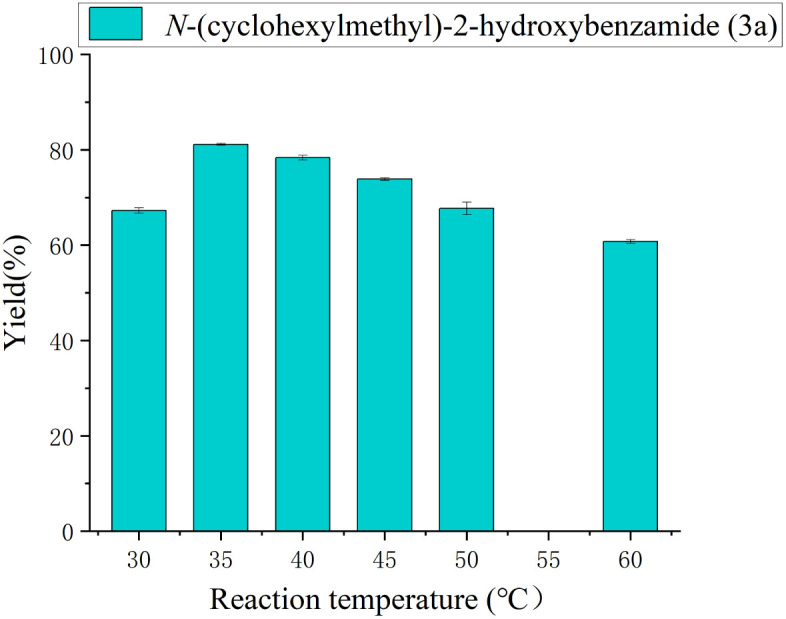
The effect of reaction temperature on the enzymatic synthesis of salicylamide derivatives in continuous-flow microreactors.

The influence of reaction temperature on the product yield was investigated from 30 to 60 °C. Before reaching the optimal temperature, the reaction rate increases stably due to the accelerated molecular motion and the increased collision frequency between the substrate and the enzyme. However, after exceeding the optimal temperature, the catalytic activity of the enzyme would decline, causing the reaction rate to drop. As shown in Fig. 4, the maximum yield was obtained at 35 °C.

### Effect of residence time

**Fig. 5:**
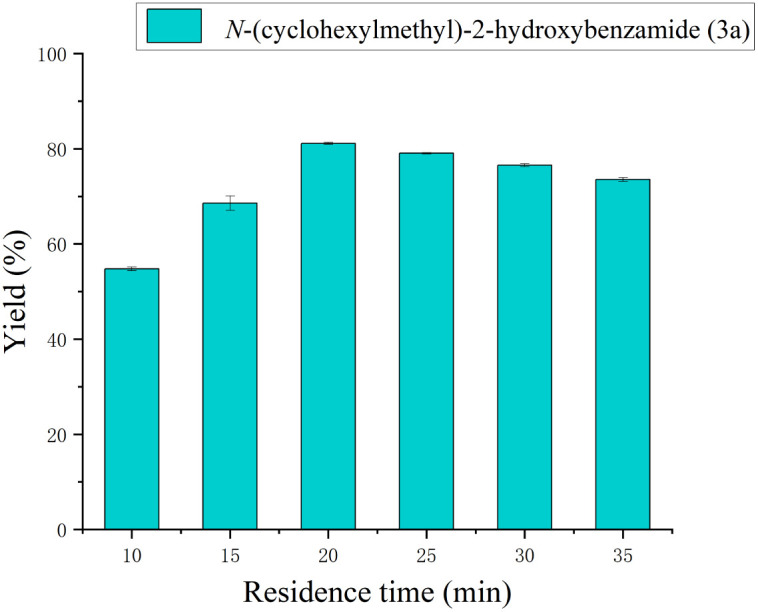
The effect of residence time on the synthesis of salicylamide derivatives in continuous-flow microreactors.

The optimal residence time typically depends on the type of reaction, the characteristics of the enzyme, the substrate concentration, and the reaction conditions. Here, the effect of reaction residence time on the synthesis of salicylamide derivatives in the range of 10 to 35 minutes was studied. With the increase of time, the yield of salicylamide derivatives gradually rises, as shown in Fig. 5. When the reaction time was 20 minutes, the reaction yield was 81.3%, which reached the highest yield. With the increase of reaction time, the yield of the reaction decreases gradually. Therefore, 20 minutes is chosen as the optimal reaction time for the synthesis of salicylanilide derivatives in continuous flow microreactors.

### Effect of enzyme reusability in a continuous-flow microreactor and a shaker reactor

**Fig. 6:**
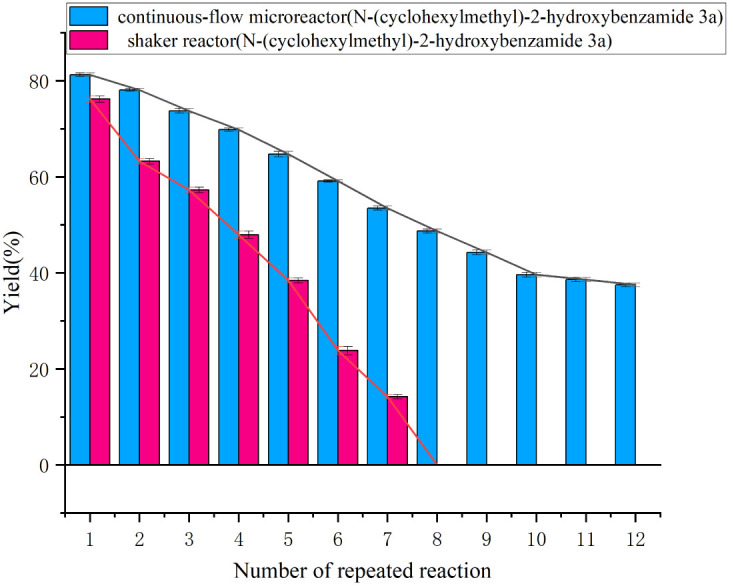
The effect of enzyme reusability in a continuous-flow microreactor and a shaker reactor.

Since the reuse of immobilized lipase can reduce production costs, we evaluated the reusability of the lipase in both a continuous-flow microreactor and a shaker reactor. After 12 catalytic cycles under identical conditions using the same enzyme sample, the catalytic yield in the continuous-flow microreactor remained at 37.8% in the final cycle. In contrast, the lipase lost its catalytic activity entirely after eight cycles in the shaken flask reactor (Fig. 6).

### Synthesis of *N*-salicyltryptamine derivatives

**Fig. 7:**
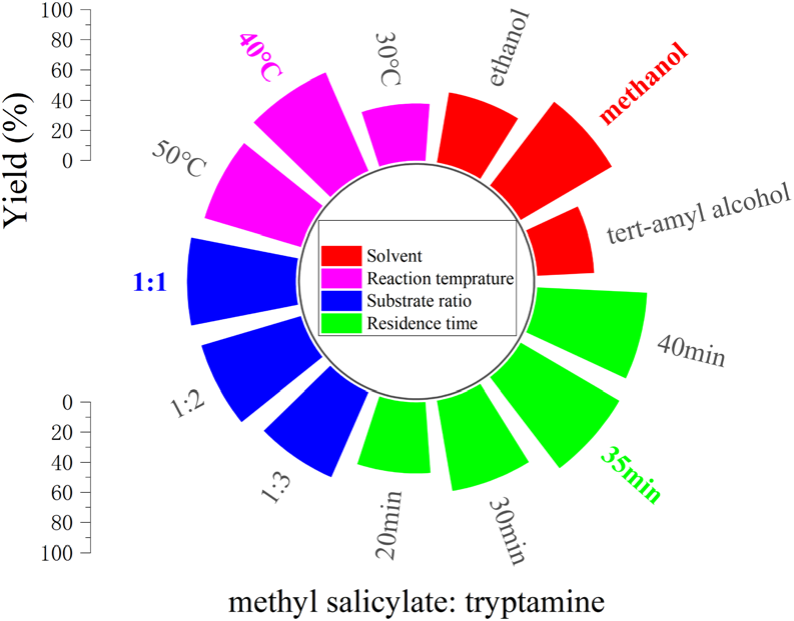
The effect of reaction parameters on the synthesis of *N*-salicyltryptamine derivatives.

**Fig. 8:**
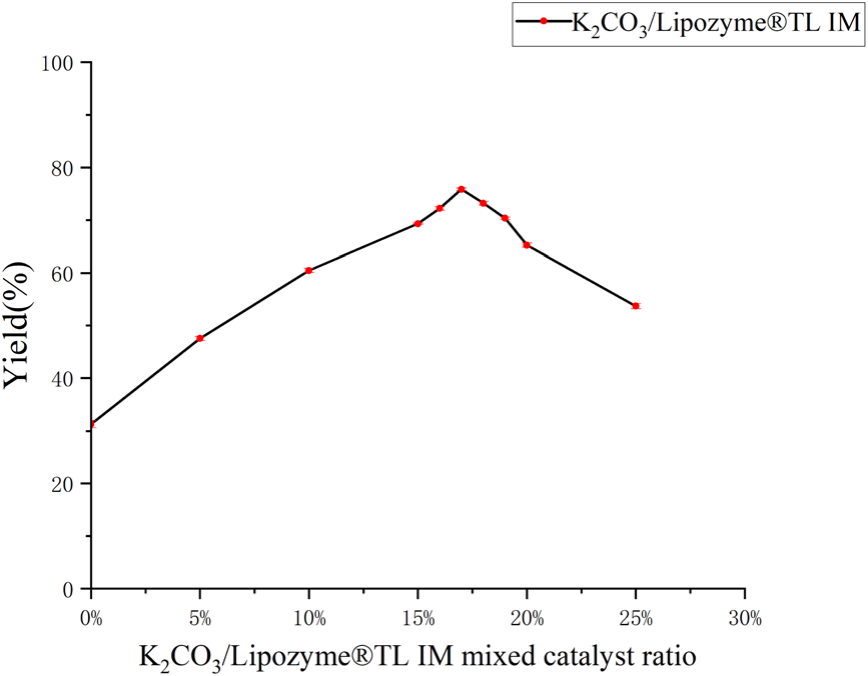
K_2_CO_3_/Lipozyme®TL IM mixed catalyst ratio of *N*-salicyltryptamine.

Tryptamine is an important natural biogenic amine that serves as a precursor for many biologically active molecules. Its core activity is closely associated with the presence of the indole ring and the primary amino group. *N*-salicyltryptamine derivatives have been investigated for various biological activities, including effects on neurodegenerative diseases, antidepressant properties, and antioxidant capacity. We explored the Lipozyme® TL IM-catalyzed ammonolysis reaction from salicylate and tryptamine in a continuous-flow microreactor. The results showed that the yield of *N*-salicyltryptamine catalyzed by Lipozyme® TL IM was 31.2%. Given the low efficiency of Lipozyme® TL IM alone in catalyzing the synthesis of *N-*salicyltryptamine derivatives, a cooperative catalysis system was introduced. After evaluating alternatives with similar mild basic properties, such as sodium carbonate (Na_2_CO_3_) and sodium bicarbonate (NaHCO_3_), potassium carbonate (K_2_CO_3_) was ultimately selected to enhance the reaction efficiency. As a weak to moderately strong base (pH ≈ 11.6 in aqueous solution), K_2_CO_3_ can effectively activate the nucleophilicity of the amino group (–NH_2_) in tryptamine to promote amide bond formation, without deactivating Lipozyme® TL IM (which would occur with strong bases like NaOH and KOH). Potassium carbonate (K_2_CO_3_) generates ions in the reaction system to reduce the bond energy of reactant molecules and the reaction activation energy, thereby accelerating the reaction rate, shortening the equilibrium time and facilitating amide bond synthesis. These findings confirmed that K_2_CO_3_ exhibits excellent adaptability for the present reaction system. Using the reaction of methyl salicylate with tryptamine as a template, we explored the optimal reaction parameters and ultimately determined the following conditions: reaction at a molar ratio of 1 : 1 in methanol at 40 °C for 35 min (Fig. 7), the optimal mixed catalyst ratio was determined to be 17% K_2_CO_3_/Lipozyme® TL IM (Fig. 8). *N*-Salicyltryptamine derivatives 5a–t were obtained through the synergistic catalysis of enzymes and potassium carbonate (K_2_CO_3_) in Table 2.

**Table 2:** Synthesis of *N*-salicyltryptamine derivatives 5a–t^.^^a^

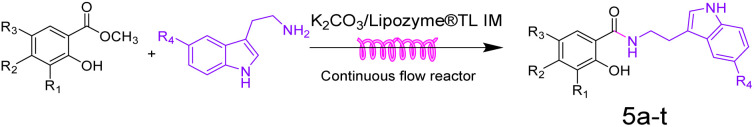				
Compound	R_1_	R_2_	R_3_	R_4_
5a	H	H	H	H
5b	H	H	Br	H
5c	H	CH_3_	H	H
5d	H	H	H	OCH_3_
5e	CH_3_	H	H	OCH_3_
5f	H	CH_3_	H	Cl
5g	H	H	H	Cl
5h	H	H	CH_3_	H
5i	H	H	Cl	H
5j	H	H	Cl	OCH_3_
5k	H	H	Br	OCH_3_
5l	H	H	Br	Cl
5m	CH_3_	H	H	Cl
5n	H	H	CH_3_	Cl
5o	H	H	OCH_3_	H
5p	H	H	OCH_3_	OCH_3_
5q	H	H	OCH_3_	Cl
5r	H	CH_3_	H	OCH_3_
5s	CH_3_	H	H	H
5t	OCH_3_	H	H	OCH_3_

a General experimental conditions: continuous flow reactors, 5 mmol methyl salicylate, 5 mmol tryptamines, methanol, flow rate 20.8 µL min^−1^, residence time 35 min, 0.1479 g K_2_CO_3_ + 0.7221 g Lipozyme®TL IM, 40 °C.

### Comparison of space-time yield in continuous flow microreactors and shaker reactors

Methyl salicylate reacting with cyclohexanemethylamine serving as a model reaction. Space-time yield (STY) was used as a metric to compare the efficiency of the enzymatic reaction between the continuous-flow microreactor and the shaker reactor. As summarized in Table 3, STY was higher in the continuous-flow microreactor, demonstrating that this system is more suitable for the synthesis of salicylamide derivatives. The reaction required 16 hours to reach completion in the conventional shaker reactor (Method B), whereas it was accomplished in 20 minutes within the continuous-flow microreactor (Method A).
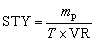
where *m*_p_ is the mass of the generated product (g),*T* is the residence time (h),and VR is the reactor volume (L).

**Table 3:** Enzymatic synthesis of salicylamide derivatives in a continuous-flow microreactor and a shaker reactor^a^

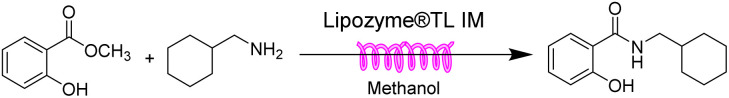			
Entry	Method	STY(g L^−1^ h^−1^)	Yield^b^ (%)
1	A	284.57	81.3 ± 0.9%
2	B	1.12	76.5 ± 1.2%

a General experimental conditions: Method A: continuous flow reactors, feed 1, dissolve 5 mmol of methyl salicylate in 10 mL methanol; feed 2, dissolve 10 mmol of cyclohexanemethylamine in 10 mL methanol, flow rate 31.2 µL min^−1^, residence time 20 min, enzyme 870 mg, 35 °C. Method B: shaker reactors, add 5 mmol of methyl salicylate, 10 mmol of cyclohexanemethylamine and 20 mL methanol to a 50 mL erlenmeyer flask, Lipozyme®TL IM 870 mg, 160 rpm, 35 °C, 16 h.

b Isolated yield. Yield: 100× (actual obtained amount/calculated amount). The data are presented as average ± SD of triplicate experiments.

### The scope and limitation of the synthesis of salicylamide derivatives catalyzed by lipozyme TL IM in continuous-flow microreactors

As shown in Table 4, salicylate esters and amines were used for the synthesis of salicylamide derivatives in continuous flow microreactors and shaker reactors. Under the same reaction conditions, different reaction times and yields were obtained. Based on this foundation, we explored the scope and limitations of the biocatalytic ammonolysis for the synthesis of salicylamide derivatives. As summarized in Table 4, benzylamines bearing electron-donating groups (4c, 72.6%) were more favorable for the reaction than those with electron-withdrawing groups (4b, 62.6%). Concurrently, for cyclic aliphatic amines, salicylate esters containing an electron-withdrawing substituent (3b, 84.1%) yielded higher product formation compared to the unsubstituted salicylate ester (3a, 81.3%). Salicylate esters with 3-position electron-donating substituents (5s, 87.1%) afford higher yields in the amidation with tryptamine than their 4-substituted analogues (5c, 71.3%) or 5-substituted analogues (5h, 72.9%).

**Table 4:** Comparison of the synthesis of salicylamide derivatives in continuous flow microreactors and shaker reactors^a^

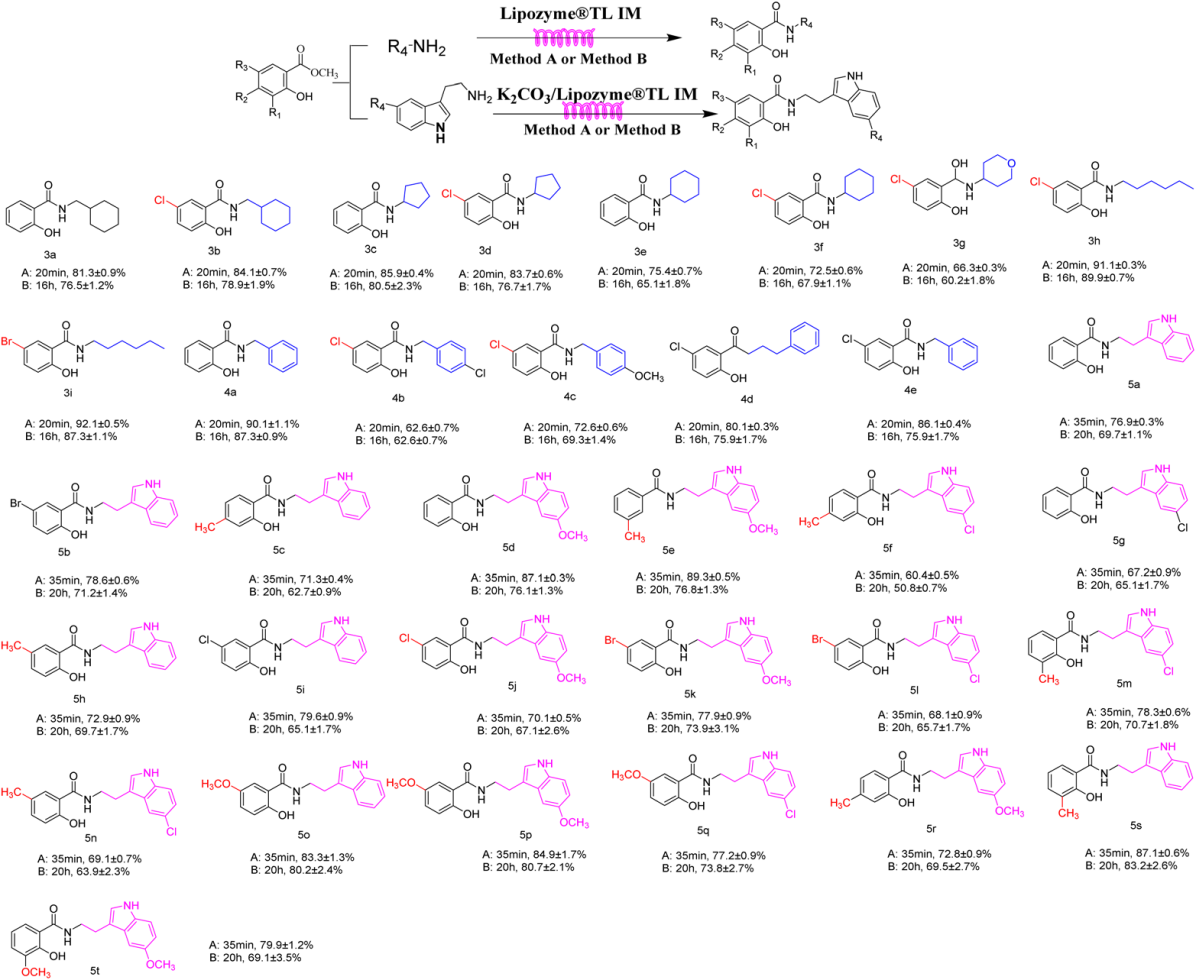

a General experimental conditions: Method A: continuous flow reactors, feed 1, dissolve 5 mmol of methyl salicylate in 10 mL methanol; feed 2, dissolve 10 mmol of amines in 10 mL methanol or 5 mmol of tryptamines in 10 mL methanol, flow rate 31.2 or 20.8 µL min^−1^, residence time 20 or 35 min, Lipozyme®TL IM 870 mg or 0.1479 g K_2_CO_3_ + 0.7221 g Lipozyme®TL IM, 35 or 40 °C. Method B: shaker reactors, add 5 mmol of methyl salicylate, 10 mmol of amines in 20 mL methanol to a 50 mL erlenmeyer flask or 5 mmol of methyl salicylate, 5 mmol tryptamines in 20 mL methanol to a 50 mL erlenmeyer flask, Lipozyme®TL IM 870 mg or 0.1479 g K_2_CO_3_ + 0.7221 g Lipozyme®TL IM, 160 rpm, 35 or 40 °C, 16 or 20 h.

## Experimental section

**Fig. 9:**
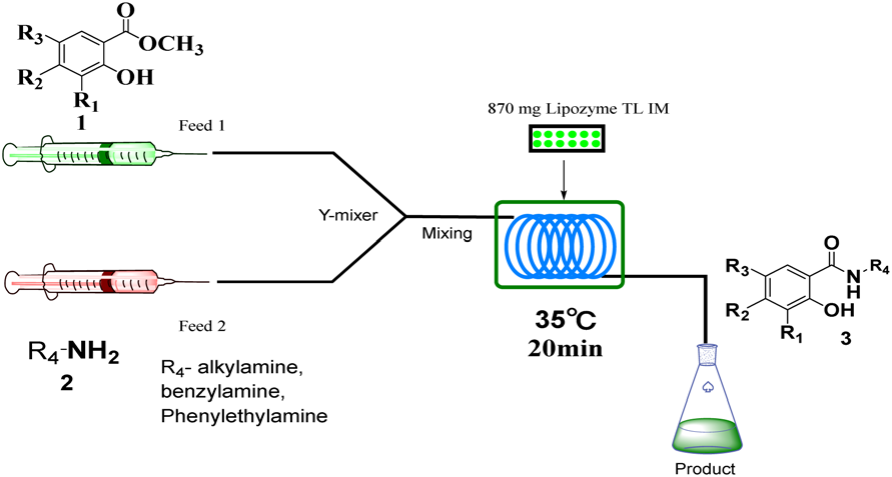
The equipment diagram for the synthesis of salicylamide derivatives in the continuous-flow microreactor catalyzed by Lipozyme® TL IM.

**Fig. 10:**
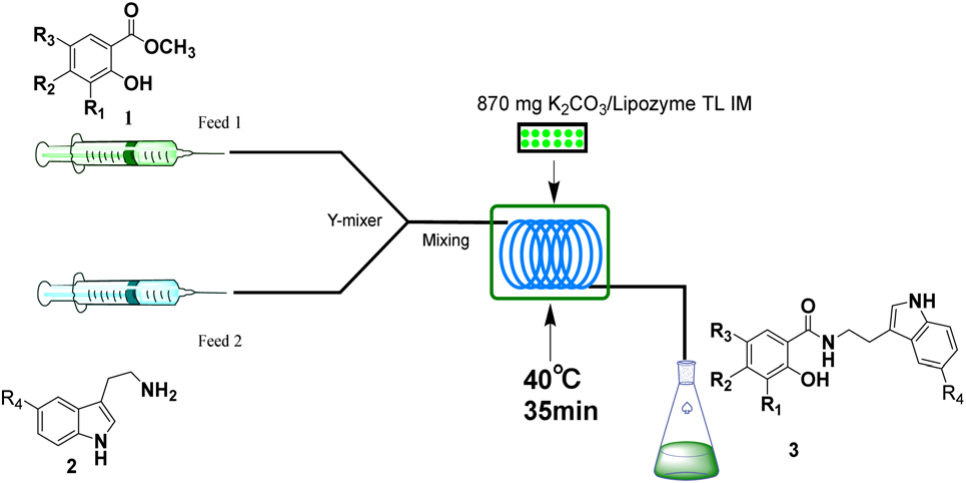
The equipment diagram for the synthesis of *N*-salicyltryptamine derivatives in the continuous-flow microreactor catalyzed by K_2_CO_3_/Lipozyme® TL IM.

The equipment diagram for the synthesis of salicylamide derivatives in the continuous-flow microreactor is depicted in Fig. 9 and 10. The experimental setup consists of a syringe pump (Harvard Apparatus Dr 2000), two substrate injectors, a *Y*-mixer, a flow reactor with 100 cm × 2 mm PFA tubing and a product collector. Silica gel tubes were filled with 870 mg of Lipozyme® TL IM from *Thermomyces lanuginosus* (reactivity 250 IUN g^−1^) or 870 mg K_2_CO_3_ + Lipozyme® TL IM and then immersed in a constant temperature water bath at 35 °C or 40 °C. 5 mmol of methyl salicylate derivatives were dissolved in 10 mL methanol (feed 1) and 10 mmol of amines (cyclic and linear aliphatic amines, aryl alkyl amines) were dissolved in 10 mL methanol (feed 2). Feed 1 and 2 were delivered to the *Y*-mixer at a flow rate of 31.2 µL min^−1^ with a residence time of 20 min (Fig. 9). 5 mmol of methyl salicylate derivatives were dissolved in 10 mL methanol (feed 1), and 5 mmol of amines (tryptamines) were dissolved in 10 mL methanol (feed 2). Feed 1 and 2 were delivered to the *Y*-mixer at a flow rate of 20.8 µL min^−1^ with a residence time of 35 min (Fig. 10). The resulting stream was connected to a sample vial to collect the final mixture. The main products were separated by silica gel chromatography and were confirmed by ^1^H NMR, ^13^C NMR.

## Conclusion

In conclusion, this study successfully developed a green and efficient synthetic strategy for salicylamide derivatives by integrating continuous flow technology with biocatalysis. Lipozyme®TL IM from *Thermomyces lanuginosus* was first used for the synthesis of salicylamide derivatives in a continuous flow microreactor. Systematic investigation and optimization of reaction parameters (solvent, enzyme type, substrate ratio, temperature, residence time, and K_2_CO_3_/Lipozyme®TL IM mixing ratio) provided a reliable technical basis for efficient synthesis. Substrate structure and electronic effect studies, using salicylate esters with different substituted groups and diverse amines (cyclic/linear aliphatic, arylalkyl, tryptamines), clarified their influence on the reaction. Comparative experiments between continuous flow and batch operations (shaker reactor) under the same reaction parameters were conducted. Quantitative evaluation based on space-time yield (STY) showed that the continuous flow technology improved the production efficiency, shortened the reaction time and increased the yield. Substrate scope studies verified the universality of this integrated technology, which enabled the one-step enzymatic aminolysis from salicylate esters and various amines to synthesize 34 distinct salicylamide derivatives. Compared with the reported works, this method features mild reaction conditions (methanol, 35 or 40 °C), shorter reaction time (20 or 35 min), a simple post-treatment process (methanol), and readily available catalyst. Overall, this continuous flow enzymatic process advances sustainable and green synthesis by enhancing efficiency, increasing yield, and shortening reaction time, aligning with green chemistry principles. Future work will focus on expanding the substrate library to access more complex analogs and extending this versatile platform to the synthesis of other valuable amide-based compounds.

## Conflicts of interest

There are no conflicts to declare.

## Supplementary Material

RA-016-D5RA09739H-s001

## Data Availability

The authors confirm that the data supporting the findings of this study are available within its supplementary information (SI). Supplementary information is available. See DOI: https://doi.org/10.1039/d5ra09739h.
