# Supplementary material for: Enzymatic aminolysis in continuous flow: an efficient strategy for the synthesis of salicylamide derivatives
Source: RSC Adv. 2026 Mar 2;16(13):11744–51. doi: 10.1039/d5ra09739h (PMC12951286; doi:10.1039/d5ra09739h)
Supplement: RA-016-D5RA09739H-s001 [file RA-016-D5RA09739H-s001.pdf]

Supplementary Information (SI) for RSC Advances.
This journal is © The Royal Society of Chemistry 2026

Supporting Information for

Enzymatic Aminolysis in Continuous Flow: An Efficient Strategy for the Synthesis of Salicylamide Derivatives

Li-Hua Du, *[a] Miao-Miao Xue, [a] Bing-Lin Yan, [a] Lin Wang , [a] Xi-Ping Luo,*[b]

Materials

Unless otherwise stated, all chemicals were obtained from commercial sources and used without further purification. Lipozyme® TL IM from *Thermomyces lanuginosus* was purchased from Novo Nordisk (Copen hagen, Denmark). Methyl salicylate, Methyl 5-chloro-2-hydroxybenzoate, Methyl 4-methylsalicylate, Methyl 2-Hydroxy-3-methylbenzoate were purchased from Meryer (Shanghai) Chemical Technology Co., Ltd.. cycl ohexanemethylamine, Cyclohexylamine, 4-Chlorobenzylamine, 2-PhenylethylaMine were purchased from Sh anghai Bepharm Science&Technology Co., Ltd.. Harvard Apparatus PHD 2000 was purchased from Harvar d University (Holliston, Massachusetts, USA). The flow reactor and Y-mixer were purchased from Beijing Haigui Medical Engineering Design Co., Ltd (Beijing China). A 400 MHz NMR spectrometer (Billerica, MA, USA) was also used in this study.

Purification of the product

When the conversion of the salicylamide derivative reached its maximum (as determined by TLC), the reaction was terminated by filtering off the enzyme. Since methanol was used as the solvent for the reaction, the reaction mixture was first subjected to reduced-pressure distillation to remove the methanol, yielding a crude product as an oil. The product was purified using silica gel chromatography (mobile phase: petroleum ether/ethyl acetate, 6/1). Furthermore, as the synthesis of the *N*-salicyltryptamine derivatives involved the use of potassium carbonate(K_2CO_3), the reaction mixture was collected and filtered under vacuum using a Büchner funnel equipped with a filter aid (such as diatomaceous earth) to retain the very fine potassium carbonate particles, yielding a clear filtrate, which was then concentrated. The purification process was monitored by thin-layer chromatography (TLC). The fractions containing the main product were combined, the solvent was evaporated, and the residue was analyzed by ^{1}H NMR and ^{13}C NMR.

Experimental setup

Figure 1 describes the continuous flow scheme for synthesizing salicylamide derivatives through salicylate

esters and amines(cyclic and linear aliphatic amines, aryl alkyl amines). The experimental setup consists of a microsystem device: two syringe pumps, a coil reactor, and a Y-shaped mixer (ϕ = 1.8 mm). Separate feed streams were introduced using syringe pumps (Harvard Apparatus PHD 2000) into a 3.1 mL PFA coil reactor (2.0 mm I.D.). In microreactor, a solution of salicylate (methyl salicylate, methyl 5-chlorosalicylate, methyl 4-methylsalicylate) was contacted with an amine solution to form salicylamide derivatives, Coil reactor 1 was filled with Lipozyme® TL IM (catalyst activity: 250 IU g^{-1}). Figure 2 describes the continuous flow scheme for synthesizing *N*-salicyltryptamine derivatives through salicylate esters and tryptamines, coil reactor 2 was filled with K_2CO_3/Lipozyme® TL IM. Both were submerged into a thermostatic water bath to control the reaction temperature. The final products stream exiting this unit is directed into collection bottles.

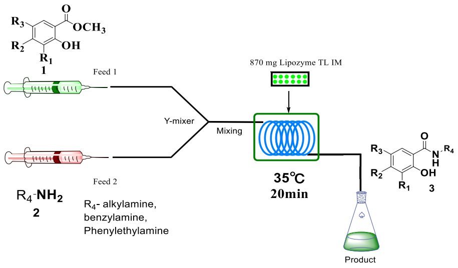

Figure 1. The equipment diagram for the synthesis of salicylamide derivatives in the continuous-flow microreactor catalyzed by Lipozyme® TL IM.

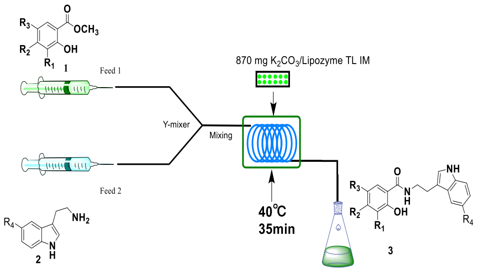

Figure 2. The equipment diagram for the synthesis of *N*-Salicyltryptamine derivatives in the continuous-flow microreactor catalyzed by K_2CO_3/Lipozyme® TL IM .

Experiment

General Procedure for salicylamide derivatives Synthesis in Continuous Flow Microreactors.

Method A (Reaction method under microfluidics):

5 mmol of salicylate Esters (methyl salicylate, methyl 5-chlorosalicylate, methyl 5-bromosalicylate, methyl 4-methylsalicylate) were taken and added to 10 mL of methanol solution and dissolved to obtain a methanol solution of 0.5 mmol mL^{-1} of salicylate Esters (feed 1); 10 mmol of amines (cyclohexanemethylamine, cyclohexylamine, cyclopentylamine, 4-aminotetrahydropyran, hexylamine, benzylamine, p-chlorophenylamine, 4-Chlorobenzylamine, 4-methoxybenzylamine) were taken and added to methanol to prepare 10 mL of solution (feed 2). A mixture of 0.87 g of Lipozyme® TL IM was evenly filled in the reaction tube. Feeds 1 and 2 were placed in separate 10 mL feeders and mixed together in a Y-mixer. The residence time was 20 min and the reaction temperature was controlled by a water bath thermostat at 35 °C. The reaction solution in the two feeds was mixed at a flow rate of 31.2 μL min^{-1} by a microfluidic pump through a Y-mixer and then fed into the reaction channel. The resulting stream (31.2 μL min^{-1}) was connected to a sample vial for collection of the final mixture. 5

mmol of salicylate esters (methyl salicylate, 5-chlorosalicylate, 5-bromosalicylate, 5-methylsalicylate, 5-methoxy salicylate, 4-methylsalicylate, 3-methylsalicylate, 3-methoxy salicylate) were taken and added to 10 mL of methanol solution and dissolved to obtain a methanol solution of 0.5 mmol mL^{-1} of salicylate esters (feed 1); 5 mmol of amines(tryptamine, 5-chlorotryptamine, 5-methoxytryptamine) were taken and added to methanol to prepare 10 mL of solution (feed 2). A mixture of 0.87 g of catalyst (0.1479 g of K_2CO_3 and 0.7221 g of Lipozyme® TL IM) was evenly filled in the reaction tube. Feeds 1 and 2 were placed in separate 10 mL feeders and mixed together in a Y-mixer. The residence time was 35 min and the reaction temperature was controlled by a water bath thermostat at 40 °C. The resulting stream (20.8 μL min^{-1}) was connected to a sample vial for collection of the final mixture. The reaction was followed up qualitatively by thin layer chromatography TLC. After the reaction was completed, the reactants were post-treated and the pure compounds obtained were subjected to structural characterization. Since methanol was used as the solvent for the reaction, the reaction mixture was first subjected to reduced-pressure distillation to remove the methanol, yielding a crude product as an oil. The product was purified using silica gel chromatography (mobile phase: petroleum ether/ethyl acetate, 6/1).

General Procedure for salicylamide derivatives Synthesis in Shaker Reactor.

Method B (Reaction method under shaking conditions):

5 mmol of salicylate esters (methyl salicylate, methyl 5-chlorosalicylate, methyl 5-bromosalicylate, methyl 4-methylsalicylate) were placed in a 50 mL conical flask and dissolved by adding 20 mL of methanol. This was followed by the addition of 10 mmol of amines (Cyclohexanemethylamine, cyclohexylamine, cyclopentylamine, 4-aminotetrahydropyran, hexylamine, benzylamine, p-chlorophenylamine, 4-Chlorobenzylamine, 4-methoxybenzylamine), diethylene-adipate, which was shaken until mixed, and 0.87 g of Lipozyme® TL IM was added. The conical flask was placed in a water bath thermostatic shaking reactor at 35 °C and 180 r.p.m. and the reaction was followed qualitatively by TLC. 5 mmol of salicylate esters (methyl salicylate, 5-chlorosalicylate, 5-bromosalicylate, 5-methylsalicylate, 5-methoxy salicylate, 4-methylsalicylate, 3-methylsalicylate, 3-methoxy salicylate) were placed in a 50 mL conical flask and dissolved by adding 20 mL of methanol. This was followed by the addition of 5 mmol of amines (tryptamine, 5-chlorotryptamine, 5-methoxytryptamine), diethylene-adipate, which was shaken until mixed, and 0.87 g catalyst (0.1479 g K_2CO_3 + 0.7221 g Lipozyme® TL IM) was added. The conical flask was placed in a water bath thermostatic shaking reactor at 40 °C and 180 r.p.m. and the reaction was followed qualitatively by TLC.

The reaction was monitored qualitatively by TLC for 16-20 h at 35-40 °C and 180 r.p.m. in a water bath with a thermostatic shaker. At the end of the reaction, the reactants were post-treated and the resulting pure

compounds were subjected to structural characterization. Since the reaction took methanol as solvent, the reaction solution was first distilled under reduced pressure to remove methanol from the reaction solution. The silica gel used for the column chromatography was 300-400 mesh, wet loaded and purified using petroleum ether/ethyl acetate = 6:1 as eluent.

Thin Layer Chromatography

Analytical TLC was performed on silica gel 60 plates (Merck) using petroleum ether/ethyl acetate (6:1, v/v) as the developing solvent. Spots were detected by UV irradiation at 254 nm.

Experimental data of products

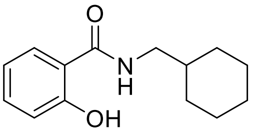

***N*-(cyclohexylmethyl)-2-hydroxybenzamide(3a).[1]** white solid, isolated yield 81.3%, m.p. 102℃，Purity＞99.5%. ^{1}H NMR (400 MHz, DMSO-d6) δ 12.82 (s, 1H), 8.81 (t, J = 5.9 Hz, 1H), 7.91 (d, J = 8.0 Hz, 1H), 7.36 (t, J = 7.8 Hz, 1H), 7.03 – 6.68 (m, 2H), 3.15 (t, J = 6.4 Hz, 2H), 1.80 – 1.60 (m, 4H), 1.61 – 1.46 (m, 2H), 1.25 – 1.02 (m, 3H), 1.00 – 0.80 (m, 2H). ^{13}C NMR (101 MHz, DMSO-d6) δ 169.55, 160.83, 133.89, 128.02, 118.75, 117.80, 115.59, 45.54, 37.79, 30.95(2C), 26.48, 25.88(2C).

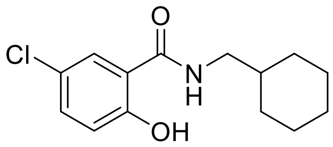

5-Chloro-*N*-(cyclohexylmethyl)-2-hydroxybenzamide(3b).[2] white solid, isolated yield 84.1%, m.p. 112℃，Purity＞99.5%. ^{1}H NMR (400 MHz, DMSO-d6) δ 12.75 (s, 1H), 8.86 (t, J = 5.8 Hz, 1H), 7.97 (d, J = 2.6 Hz, 1H), 7.41 (d, J = 8.8 Hz, 1H), 6.92 (d, J = 8.8 Hz, 1H), 3.13 (t, J = 6.3 Hz, 2H), 1.74 – 1.48 (m, 6H), 1.26 – 1.04 (m, 3H), 0.98 – 0.84 (m, 2H). ^{13}C NMR (101 MHz, DMSO-d6) δ 168.09, 159.26, 133.61, 127.58, 122.69, 119.74, 117.06, 45.68, 37.64, 30.90(2C), 26.44, 25.84(2C).

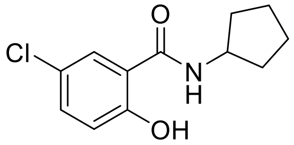

5-Chloro-*N*-cyclopentyl-2-hydroxybenzamide (3c).[2] white solid, isolated yield 85.9%, m.p. 129℃，Purity＞99.5%. ^{1}H NMR (400 MHz, DMSO-d6) δ 12.80 (s, 1H), 8.69 (d, J = 4.1 Hz, 1H), 8.02 (d, J = 2.6 Hz, 1H), 7.41 (d, J = 7.9 Hz, 1H), 6.91 (d, J = 9.0 Hz, 1H), 4.47 – 4.06 (m, 1H), 2.00 –

1.80 (m, 2H), 1.78 – 1.40 (m, 6H). ^{13}C NMR (101 MHz, DMSO-d6) δ 167.84, 159.39, 133.66, 127.63, 122.61, 119.71, 116.91, 51.34, 32.38(2C), 24.11(2C).

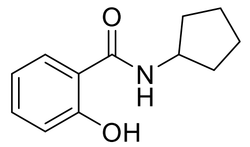

***N*-cyclopentyl-2-hydroxybenzamide (3d).[3]** white solid, isolated yield 83.7%, m.p. 119℃ Purity＞99.5%. ^{1}H NMR (400 MHz, DMSO-d6) δ 12.73 (s, 1H), 8.59 (d, J = 7.2 Hz, 1H), 7.92 (dd, J = 7.9, 1.7 Hz, 1H), 7.38 (t, J = 7.8 Hz, 1H), 6.92 – 6.83 (m, 2H), 4.31 – 4.22 (m, 1H), 1.98 – 1.84 (m, 2H), 1.78 – 1.64 (m, 2H), 1.64 – 1.49 (m, 4H). ^{13}C NMR (101 MHz, DMSO-d6) δ 169.18, 160.70, 133.99, 128.28, 118.76, 117.76, 115.63, 51.20, 32.43(2C), 24.09(2C).

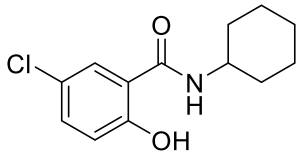

5-Chloro-*N*-cyclohexyl-2-hydroxybenzamide(3e).[2] white solid, isolated yield 75.4%, m.p. 148℃ Purity＞99.5%. ^{1}H NMR (400 MHz, DMSO-d6) δ 12.76 (s, 1H), 8.64 (d, J = 7.8 Hz, 1H), 8.00 (d, J = 2.6 Hz, 1H), 7.42 (d, J = 8.7 Hz, 1H), 6.92 (d, J = 9.1 Hz, 1H), 4.00 – 3.65 (m, 1H), 1.93 – 1.66 (m, 4H), 1.60 (d, J = 13.0 Hz, 1H), 1.41 – 1.07 (m, 5H). ^{13}C NMR (101 MHz, DMSO-d6) δ 167.15, 159.30, 133.65, 127.66, 122.66, 119.73, 117.09, 48.79, 32.50(2C), 25.60, 25.20(2C).

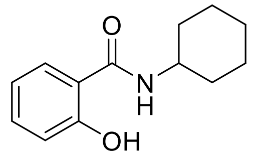

***N*-cyclohexyl-2-hydroxybenzamide(3f).[3]** white solid, isolated yield 72.5%, m.p. 139℃ Purity＞99.5%. ^{1}H NMR (400 MHz, DMSO-d6) δ 12.71 (s, 1H), 8.53 (d, J = 7.9 Hz, 1H), 7.91 (d, J = 7.8 Hz, 1H), 7.53 – 7.25 (m, 1H), 6.97 – 6.70 (m, 2H), 3.88 – 3.64 (m, 1H), 1.89 – 1.67 (m, 4H), 1.64 – 1.55 (m, 1H), 1.40 – 1.10 (m, 5H). ^{13}C NMR (101 MHz, DMSO-d6) δ 168.52, 160.67, 133.96, 128.27, 118.77, 117.76, 115.73, 48.60, 32.59(2C), 25.63, 25.27(2C).

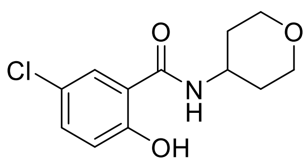

5-Chloro-2-hydroxy-*N*-(tetrahydro-2H-pyran-4-yl)benzamide(3g).[2] white solid, isolated yield 66.3%, m.p. 162℃ Purity＞99.5%. ^{1}H NMR (400 MHz, DMSO-d6) δ 12.68 (s, 1H), 8.89 (t, J = 5.7 Hz, 1H), 7.96 (d, J = 2.7 Hz, 1H), 7.40 (s, 1H), 6.93 (d, J = 6.1 Hz, 1H), 3.88 – 3.80 (m, 2H), 3.31 – 3.15 (m, 4H), 1.87 – 1.73 (m, 1H), 1.62 – 1.54 (m, 2H), 1.27 – 1.13 (m, 2H). ^{13}C NMR (101 MHz, DMSO-d6) δ 168.24, 159.24, 133.61, 127.62, 122.72, 119.70, 117.05, 67.17(2C), 45.17, 35.05, 30.89(2C).

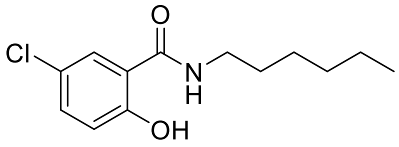

5-Chloro-*N*-hexyl-2-hydroxybenzamide (3h).[2] white solid, isolated yield 91.1%, m.p. 83℃ Purity＞99.5%. ^{1}H NMR (400 MHz, DMSO-d6) δ 12.75 (s, 1H), 8.88 (d, J = 5.6 Hz, 1H), 7.95 (d, J = 2.6 Hz, 1H), 7.42 (dd, J = 8.8, 2.6 Hz, 1H), 6.92 (d, J = 8.8 Hz, 1H), 3.27 (q, J = 6.7 Hz, 2H), 1.58 – 1.46 (m, 2H), 1.36 – 1.22 (m, 6H), 0.89 – 0.81 (m, 3H). ^{13}C NMR (101 MHz, DMSO-d6) δ 168.04, 159.30, 133.64, 127.51, 122.67, 119.76, 117.00, 39.53, 31.43, 29.10, 26.58, 22.51, 14.35.

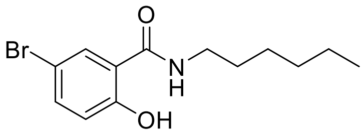

5-Bromo-*N*-hexyl-2-hydroxybenzamide (3i).[12] white solid, isolated yield 92.1%, m.p. 87℃ Purity＞99.5%. ^{1}H NMR (400 MHz, DMSO-d6) δ 12.79 (s, 1H), 8.87 (t, J = 5.7 Hz, 1H), 8.07 (s, 1H), 7.51 (d, J = 4.4 Hz, 1H), 6.87 (dd, J = 8.8, 3.0 Hz, 1H), 3.27 (q, J = 6.7 Hz, 2H), 1.65 – 1.43 (m, 2H), 1.26 (s, 6H), 0.92 – 0.78 (m, 3H). ^{13}C NMR (101 MHz, DMSO-d6) δ 167.99, 159.75, 136.43, 130.38, 120.19, 117.57, 110.07, 39.54, 31.43, 29.11, 26.59, 22.52, 14.36.

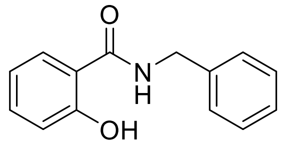

2-Hydroxy-*N*-(phenylmethyl)benzamide(4a).[4] white solid, isolated yield 90.1%, m.p. 127℃ Purity＞99.5%. ^{1}H NMR (400 MHz, DMSO-d6) δ 12.57 (s, 1H), 9.39 (t, J = 6.1 Hz, 1H), 7.92 (d, J = 6.3 Hz, 1H), 7.42 (t, J = 7.8 Hz, 1H), 7.34 (d, J = 4.4 Hz, 4H), 7.31 – 7.19 (m, 1H), 6.98 – 6.85 (m, 2H), 4.53 (d, J = 6.0 Hz, 2H). ^{13}C NMR (101 MHz, DMSO-d6) δ 169.43, 160.58, 139.45, 134.29, 128.85(2C), 128.24, 127.73(2C), 127.40, 119.13, 117.90, 115.60, 42.80.

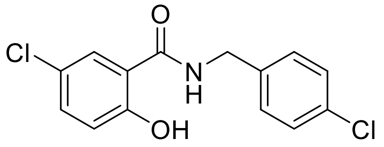

5-Chloro-*N*-[(4-chlorophenyl)methyl]-2-hydroxybenzamide(4b).[5] white solid, isolated yield 62.6%, m.p. 145℃ Purity＞99.5%. ^{1}H NMR (400 MHz, DMSO-d6) δ 12.45 (s, 1H), 9.42 (t, J = 5.7 Hz, 1H), 7.97 (d, J = 2.5 Hz, 1H), 7.49 – 7.39 (m, 2H), 7.39 – 7.33 (m, 3H), 6.96 (d, J = 8.8 Hz, 1H), 4.50 (d, J = 5.9 Hz, 2H). ^{13}C NMR (101 MHz, DMSO-d6) δ 167.93, 158.95, 138.26, 133.83, 132.03, 129.73(2C), 128.80(2C), 127.92, 122.88, 119.79, 117.32, 42.36.

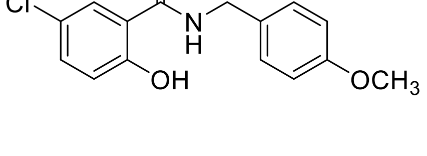

5-Chloro-*N*-[(4-methoxy)methyl]-2-hydroxybenzamide(4c).[6] white solid, isolated yield 72.6%, m.p. 147℃ Purity＞99.5%. ^{1}H NMR (400 MHz, DMSO-d6) δ 12.60 (s, 1H), 9.35 (t, J = 5.9 Hz, 1H), 7.98 (d, J = 2.7 Hz, 1H), 7.44 (dd, J = 8.9, 2.6 Hz, 1H), 7.31 – 7.23 (m, 2H), 6.95 (d, J = 8.8 Hz, 1H), 6.93 – 6.85 (m, 2H), 4.44 (d, J = 5.8 Hz, 2H), 3.73 (s, 3H). ^{13}C NMR (101 MHz, DMSO-d6) δ 167.82, 159.09, 158.83, 133.79, 131.05, 129.32(2C), 127.78, 122.82, 119.80, 117.18, 114.24(2C), 55.51, 42.48.

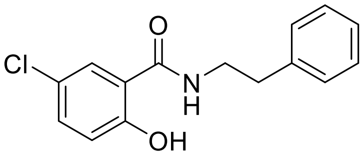

5-Chloro-2-hydroxy-*N*-(2-phenylethyl)benzamide(4d).[6] white solid, isolated yield 86.1%, m.p. 119℃ Purity＞99.5%. ^{1}H NMR (400 MHz, DMSO-d6) δ 12.57 (s, 1H), 8.95 (t, J = 5.7 Hz, 1H), 7.93 (d, J = 2.5 Hz, 1H), 7.42 (dd, J = 8.8, 1.7 Hz, 1H), 7.35 – 7.16 (m, 5H), 6.94 (d, J = 8.8 Hz, 1H), 3.59 – 3.50 (m, 2H), 2.87 (t, J = 7.4 Hz, 2H). ^{13}C NMR (101 MHz, DMSO-d6) δ 167.86, 159.00, 139.63, 133.64, 129.11, 129.11, 128.84, 128.84， 127.74, 126.67, 122.79, 119.75, 117.29, 41.12, 35.19.

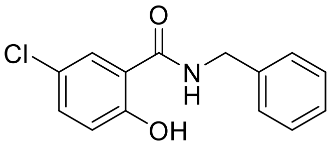

5-Chloro-N-(benzyl)-2-hydroxybenzamide(4e).[5] white solid, isolated yield 80.1%, m.p. 143℃ Purity＞99.5%. ^{1}H NMR (400 MHz, DMSO-d6) δ 12.55 (s, 1H), 9.42 (t, J = 5.6 Hz, 1H), 8.01 (d, J = 6.0 Hz, 1H), 7.44 (d, J = 6.9 Hz, 1H), 7.34 (d, J = 4.4 Hz, 4H), 7.30 – 7.21 (m, 1H), 6.96 (d, J = 8.9 Hz, 1H), 4.53 (d, J = 6.2 Hz, 2H). ^{13}C NMR (101 MHz, DMSO-d6) δ 167.99, 159.11, 139.16, 133.80, 128.85(2C), 127.85(3C), 127.46, 122.87, 119.80, 117.24, 43.01.

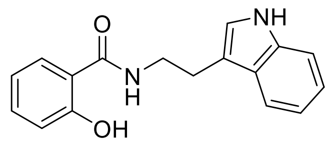

***N*-(2-(1H-indol-3-yl)ethyl)-2-hydroxybenzamide(5a).**[7] white solid, isolated yield 78.6%, m.p. 154℃, Purity＞99.5%. ^{1}H NMR (400 MHz, DMSO-d6) δ 12.72 (s, 1H), 10.85 (s, 1H), 8.98 (t, J = 5.7 Hz, 1H), 7.86 (dd, J = 8.0, 1.7 Hz, 1H), 7.60 (d, J = 7.8 Hz, 1H), 7.45 – 7.33 (m, 2H), 7.21 (d, J = 2.3 Hz, 1H), 7.14 – 7.05 (m, 1H), 7.03 – 6.96 (m, 1H), 6.95 – 6.84 (m, 2H), 3.61 (dd, J = 13.8, 6.8 Hz, 2H), 3.00 (t, J = 7.5 Hz, 2H). ^{13}C NMR (101 MHz, DMSO-d6) δ 169.43, 160.65, 136.74, 134.10, 128.10, 127.68, 123.20, 121.45, 118.99, 118.77, 118.74, 117.88, 115.70, 112.07, 111.89, 40.37, 25.40.

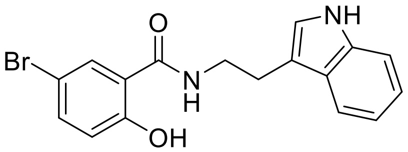

***N*-(2-(1H-indol-3-yl)ethyl)-5-bromo-2-hydroxybenzamide(5b).**[8] white solid, isolated yield 76.9%, m.p. 175℃ Purity＞99.5%. ^{1}H NMR (400 MHz, DMSO-d6) δ 12.77 (s, 1H), 10.88 (s, 1H), 9.06 (t, J = 5.6 Hz, 1H), 8.10 (d, J = 2.6 Hz, 1H), 7.60 (d, J = 7.8 Hz, 1H), 7.54 (dd, J = 8.8, 2.5 Hz, 1H), 7.38 (d, J = 8.1 Hz, 1H), 7.22 (d, J = 2.4 Hz, 1H), 7.10 (t, J = 6.9 Hz, 1H), 7.01 (t, J = 7.6 Hz, 1H), 6.90 (d, J = 8.8 Hz, 1H), 3.62 (q, J = 11.4, 3.6 Hz, 2H), 3.01 (t, J = 7.4 Hz, 2H). ^{13}C NMR (101 MHz, DMSO-d6) δ 167.94, 159.63, 136.76, 136.47, 130.59, 127.67, 123.25, 121.48, 120.22, 118.79, 118.73, 117.78, 112.00, 111.92, 110.19, 40.52, 25.27.

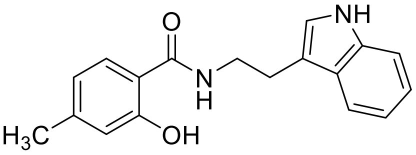

***N*-(2-(1H-indol-3-yl)ethyl)-2-hydroxy-4-methylbenzamide(5c).**[9] white solid, isolated yield 71.3%, m.p. 71℃ Purity＞99.5%. ^{1}H NMR (400 MHz, DMSO-d6) δ 12.79 (s, 1H), 10.86 (s, 1H), 8.92 (t, J = 5.5 Hz, 1H), 7.78 (d, J = 8.0 Hz, 1H), 7.62 (d, J = 6.1 Hz, 1H), 7.39 (d, J = 7.3 Hz, 1H), 7.22 (d, J = 2.7 Hz, 1H), 7.09 (t, J = 8.7 Hz, 1H), 7.01 (t, J = 7.2 Hz, 1H), 6.80 – 6.60 (m, 2H),3.61 (q, J = 14.5, 7.2 Hz, 2H), 3.02 (t, J = 7.6 Hz, 2H), 2.28 (s, 3H). ^{13}C NMR (101 MHz, DMSO-d6) δ 169.65, 160.88, 144.53, 136.76, 127.85, 127.71, 123.18, 121.45, 120.01, 118.77, 118.77, 118.04, 112.94, 112.11, 111.90, 40.32, 25.45, 21.55.

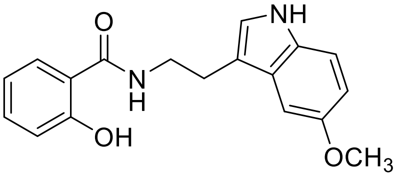

2-hydroxy-*N*-(2-(5-methoxy-1H-indol-3-yl)ethyl)benzamide(5d).[10] white solid, isolated yield 87.1%, m.p. 133℃ Purity＞99.5%. ^{1}H NMR (400 MHz, DMSO-d6) δ 12.76 (s, 1H), 10.70 (s, 1H), 8.99 (t, J = 7.2 Hz, 1H), 7.88 (s, 1H), 7.39 (d, J = 7.5 Hz, 1H), 7.27 (s, 2H), 7.09 (d, J = 8.0 Hz, 1H), 6.92 (d, J = 8.6 Hz, 1H), 6.89 (t, J = 7.8 Hz, 1H), 6.74 (s, 1H), 3.83 – 3.48 (m, 5H), 3.01 (t, J = 7.1 Hz, 2H). ^{13}C NMR (101 MHz, DMSO-d6) δ 169.50, 160.71, 153.51, 134.08, 131.89, 128.11, 124.33, 123.88, 118.96, 117.88, 115.71, 112.53, 111.99, 111.63, 100.58, 55.67, 39.92, 25.38.

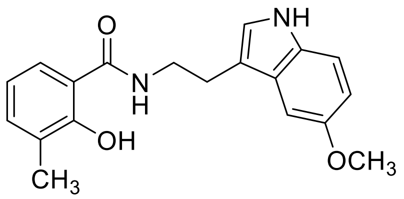

***N*-(2-(5-methoxy-1H-indol-3-yl)ethyl)-2-hydroxy-3-methylbenzamide(5e).**[11] white solid, isolated yield 89.3%, m.p. 126℃ Purity＞99.5%. ^{1}H NMR (400 MHz, DMSO-d6) δ 13.41 (s, 1H), 10.71 (s, 1H), 9.04 (t, J = 5.8 Hz, 1H), 7.71 (d, J = 8.0 Hz, 1H), 7.28 (dd, J = 9.9, 7.8 Hz, 2H), 7.18 (d, J = 2.4 Hz, 1H), 7.10 (d, J = 2.6 Hz, 1H), 6.79 (d, J = 7.6 Hz, 1H), 6.74 (s, 1H), 3.74 (s, 3H), 3.61 (q, J = 6.8 Hz, 2H), 3.00 (t, J = 7.4 Hz, 2H), 2.19 (s, 3H). ^{13}C NMR (101 MHz, DMSO-d6) δ 170.67, 159.96, 153.51, 134.89, 131.88, 128.10, 126.47, 124.98, 123.87, 118.09, 114.04, 112.54, 111.98, 111.63, 100.55, 55.65, 40.53, 25.34, 15.95.

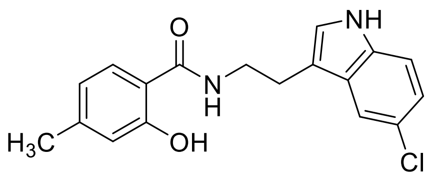

***N*-(2-(5-chloro-1H-indol-3-yl)ethyl)-2-hydroxy-4-methylbenzamide(5f).**[10] white solid, isolated yield 60.4%, m.p. 174℃ Purity＞99.5%. ^{1}H NMR (400 MHz, DMSO-d6) δ 12.75 (s, 1H), 11.08 (s, 1H), 8.91 (t, J = 5.8 Hz, 1H), 7.75 (d, J = 8.1 Hz, 1H), 7.66 (d, J = 2.2 Hz, 1H), 7.38 (d, J = 8.6 Hz, 1H), 7.29 (d, J = 2.2 Hz, 1H), 7.09 (dd, J = 8.6, 1.7 Hz, 1H), 6.74 (s, 1H), 6.70 (s, 1H), 3.58 (q, J = 4.8, 2.7 Hz, 2H), 2.98 (t, J = 7.7 Hz, 2H), 2.27 (s, 3H). ^{13}C NMR (101 MHz, DMSO-d6) δ 169.63, 160.86, 144.53, 135.19, 128.89, 127.82, 125.17, 123.58, 121.37, 120.01, 118.10, 118.03, 113.40, 112.91, 112.15, 40.25, 25.21, 21.54.

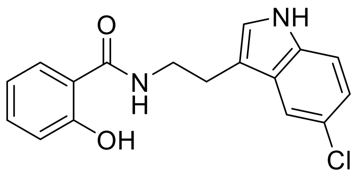

N-(2-(5-chloro-1H-indol-3-yl)ethyl)-2-hydroxybenzamide(5g).[10] white solid, isolated yield 67.2%, m.p. 135℃ Purity＞99.5%. ^{1}H NMR (400 MHz, DMSO-d6) δ 12.65 (s, 1H), 11.06 (s, 1H), 8.95 (t, J =5.6 Hz, 1H), 7.83 (d, J = 7.8 Hz, 1H), 7.63 (d, J = 2.0 Hz, 1H), 7.50 – 7.33 (m, 2H), 7.28 (d, J = 2.6 Hz, 1H), 7.07 (d, J = 8.9 Hz, 1H), 6.89 (t, J = 8.4 Hz, 2H), 3.56 (q, J = 6.9 Hz, 2H), 2.96 (t, J = 7.3 Hz, 2H). ^{13}C NMR (101 MHz, DMSO-d6) δ 169.41, 160.61, 135.18, 134.08, 128.88, 128.10, 125.19, 123.55, 121.36, 118.99, 118.09, 117.87, 115.69, 113.41, 112.12, 40.87, 25.17.

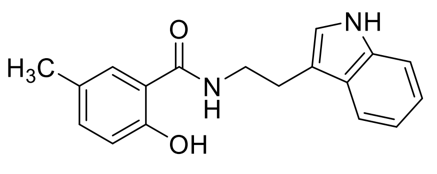

***N*-(2-(1H-indol-3-yl)ethyl)-2-hydroxy-5-methylbenzamide(5h).**[10] white solid, isolated yield 72.9%, m.p. 153℃ Purity＞99.5%. ^{1}H NMR (400 MHz, DMSO-d6) δ 12.47 (s, 1H), 10.86 (s, 1H), 8.92 (t, J = 6.2 Hz, 1H), 7.68 (s, 1H), 7.61 (d, J = 7.7 Hz, 1H), 7.37 (d, J = 8.1 Hz, 1H), 7.20 (dd, J = 6.1, 2.3 Hz, 2H), 7.09 (t, J = 7.5 Hz, 1H), 7.05 – 6.96 (m, 1H), 6.82 (d, J = 8.4 Hz, 1H), 3.60 (q, J = 8.1, 4.7 Hz, 2H), 2.99 (t, J = 7.4 Hz, 2H), 2.25 (s, 3H). ^{13}C NMR (101 MHz, DMSO-d6) δ 169.46, 158.46, 136.75, 134.73, 128.01, 127.70, 127.56, 123.18, 121.45, 118.76, 118.74, 117.65, 115.32, 112.10, 111.89, 40.33, 25.43, 20.57.

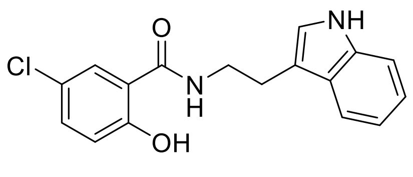

N-(2-(1H-indol-3-yl)ethyl)-5-chloro-2-hydroxybenzamide(5i).[10] white solid, isolated yield 79.6%, m.p. 171℃ Purity＞99.5%. ^{1}H NMR (400 MHz, DMSO-d6) δ 12.70 (s, 1H), 10.86 (s,

1H), 9.03 (t, J = 4.1 Hz, 1H), 7.96 (d, J = 2.6 Hz, 1H), 7.59 (d, J = 7.9 Hz, 1H), 7.43 (dd, J = 8.8, 2.5 Hz, 1H), 7.37 (d, J = 4.0 Hz, 1H), 7.21 (s, 1H), 7.09 (t, J = 6.9 Hz, 1H), 7.04 – 6.92 (m, 2H), 3.60 (q, J = 7.4, 6.7 Hz, 2H), 3.01 (t, J = 4.4 Hz, 2H). ^{13}C NMR (101 MHz, DMSO-d6) δ 167.97, 159.19, 136.76, 133.65, 127.71, 127.67, 123.24, 122.75, 121.46, 119.78, 118.77, 118.71, 117.26, 111.99, 111.90, 40.50, 25.25.

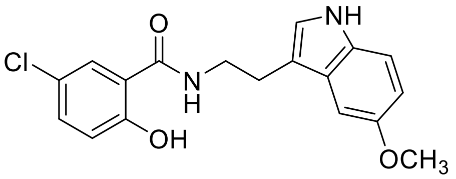

***N*-(2-(5-methoxy-1H-indol-3-yl)ethyl)-5-chloro-2-hydroxybenzamide(5j).[11]** white solid, isolated yield 70.1%, m.p. 134℃, Purity＞99.5%. ^{1}H NMR (400 MHz, DMSO-d6) δ 12.71 (s, 1H), 10.69 (s, 1H), 9.03 (t, J = 5.7 Hz, 1H), 7.96 (d, J = 2.7 Hz, 1H), 7.43 (dd, J = 8.9, 2.6 Hz, 1H), 7.25 (d, J = 8.7 Hz, 1H), 7.17 (d, J = 2.4 Hz, 1H), 7.07 (d, J = 2.4 Hz, 1H), 6.95 (d, J = 8.8 Hz, 1H), 6.73 (dd, J = 8.7, 2.4 Hz, 1H), 3.74 (s, 3H), 3.60 (q, J = 7.1 Hz, 2H), 2.97 (t, J = 7.3 Hz, 2H). ^{13}C NMR (101 MHz, DMSO-d6) δ 167.99, 159.17, 153.51, 133.66, 131.87, 128.04, 127.71, 126.89, 123.92, 122.76, 119.77, 117.24, 112.54, 111.88, 111.63, 100.54, 55.69, 25.22.

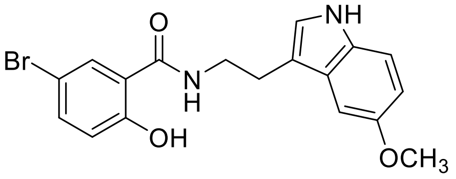

N-(2-(5-methoxy-1H-indol-3-yl)ethyl)-5-bromo-2-hydroxybenzamide(5k).[11] white solid, isolated yield 77.9%, m.p. 140℃, Purity＞99.5%. ^{1}H NMR (400 MHz, DMSO-d6) δ 12.74 (s, 1H), 10.69 (s, 1H), 9.03 (t, J = 5.5 Hz, 1H), 8.09 (d, J = 7.7 Hz, 1H), 7.54 (dd, J = 8.8, 2.5 Hz, 1H), 7.25 (d, J = 8.7 Hz, 1H), 7.17 (d, J = 8.1 Hz, 1H), 7.07 (d, J = 7.8 Hz, 1H), 6.89 (d, J = 8.8 Hz, 1H), 6.74 (dd, J = 10.5, 2.0 Hz, 1H), 3.73 (s, 3H), 3.61 (q, J = 16.3, 5.7 Hz, 2H), 2.97 (t, J = 7.5 Hz, 2H). ^{13}C NMR (101 MHz, DMSO-d6) δ 167.93, 159.61, 153.54, 153.51, 136.47, 131.87, 130.57, 128.04, 123.92, 120.21, 117.78, 112.54, 111.87, 111.63, 110.17, 100.54, 55.71, 25.22.

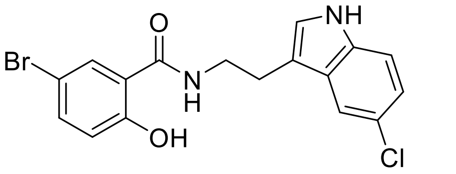

***N*-(2-(5-chloro-1H-indol-3-yl)ethyl)-5-bromo-2-hydroxybenzamide(5l).[11]** white solid, isolated yield 68.1%, m.p. 183℃, Purity＞99.5%. ^{1}H NMR (400 MHz, DMSO-d6) δ 12.67 (s, 1H), 11.07 (s, 1H), 9.01 (t, J = 5.2 Hz, 1H), 8.05 (d, J = 2.6 Hz, 1H), 7.63 (d, J = 5.4 Hz, 1H), 7.54 (dd, J = 8.7, 2.6 Hz, 1H), 7.37 (d, J = 8.6 Hz, 1H), 7.29 (d, J = 2.8 Hz, 1H), 7.07 (d, J = 8.7 Hz, 1H), 6.89 (d, J = 8.8 Hz, 1H), 3.57 (q, J = 7.4, 6.9 Hz, 2H), 2.97 (t, J = 6.9 Hz, 2H). ^{13}C NMR (101 MHz, DMSO-d6) δ 167.88, 159.52, 136.46, 135.18, 130.57, 128.86, 125.23, 123.58, 121.37, 120.20, 118.07, 117.81, 113.41, 112.05, 110.20, 40.48, 25.02.

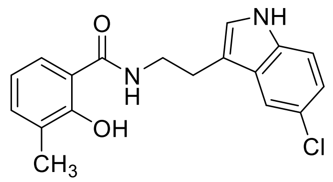

N-[2-(5-Chloro-1H-indol-3-yl)-ethyl]-2-hydroxy-3-methyl benzamide (5m).[11] white solid, isolated yield 78.3%, m.p. 175 °C, Purity＞99.5%. ^{1}H NMR (400 MHz, DMSO-d6) δ 13.31 (s, 1H), 11.12 (s, 1H), 9.05 (d, J = 3.5 Hz, 1H), 7.74 – 7.66 (m, 1H), 7.64 (d, J = 2.7 Hz, 1H), 7.37 (dd, J = 8.7, 3.9 Hz, 1H), 7.29 (d, J = 2.9 Hz, 1H), 7.29 – 7.23 (m, 1H), 7.07 (dd, J = 8.6, 2.3 Hz, 1H), 6.78 (t, J = 7.7 Hz, 1H), 3.58 (q, J = 7.1, 6.0 Hz, 2H), 3.01 (t, J = 3.9 Hz, 2H), 2.17 (s, 3H). ^{13}C NMR (101 MHz, DMSO-d6) δ 170.66, 159.94, 135.21, 134.87, 128.93, 126.47, 125.18, 124.95, 123.63, 121.38, 118.11, 118.11, 114.03, 113.40, 112.15, 40.38, 25.11, 15.96.

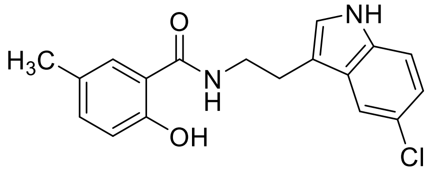

***N*-(2-(5-chloro-1H-indol-3-yl)ethyl)-2-hydroxy-5-methylbenzamide(5n).**[11] white solid, isolated yield 69.1%, m.p. 148℃, Purity＞99.5%. ^{1}H NMR (400 MHz, DMSO-d6) δ 12.40 (s, 1H), 11.06 (s, 1H), 8.90 (t, J = 5.6 Hz, 1H), 7.70 (d, J = 2.7 Hz, 1H), 7.65 (d, J = 6.0 Hz, 1H), 7.37 (dd, J = 8.6, 3.2 Hz, 1H), 7.30 (d, J = 7.8 Hz, 1H), 7.20 (d, J = 8.6 Hz, 1H), 7.08 (d, J = 8.5 Hz, 1H), 6.80 (d, J = 8.3 Hz, 1H), 3.57 (q, J = 10.2, 6.8 Hz, 2H), 2.96 (t, J = 7.1 Hz, 2H), 2.25 (s, 3H). ^{13}C NMR (101 MHz, DMSO-d6) δ 169.42, 158.41, 135.18, 134.72, 128.90, 127.99, 127.58, 125.18, 123.54, 121.36, 118.10, 117.64, 115.29, 113.40, 112.16, 40.37,25.20, 20.57.

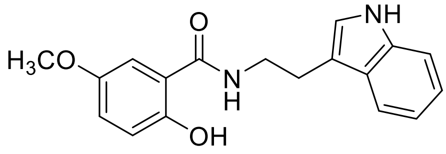

***N*-(2-(1H-indol-3-yl)ethyl)-2-hydroxy-5-methoxybenzamide(5o).**[8] white solid, isolated yield 83.3%, m.p. 133℃, Purity＞99.5%. ^{1}H NMR (400 MHz, DMSO-d6) δ 12.19 (s, 1H), 10.86 (s, 1H), 8.98 (t, J = 5.7 Hz, 1H), 7.62 (d, J = 7.8 Hz, 1H), 7.44 (d, J = 3.1 Hz, 1H), 7.38 (dd, J = 8.1, 1.1 Hz, 1H), 7.22 (d, J = 2.4 Hz, 1H), 7.10 (t, J = 7.5 Hz, 1H), 7.07 – 6.97 (m, 2H), 6.86 (d, J = 8.9 Hz, 1H), 3.74 (s, 3H), 3.62 (q, J = 13.9, 6.6 Hz, 2H), 3.01 (t, J = 7.4 Hz, 2H). ^{13}C NMR (101 MHz, DMSO-d6) δ 169.06, 154.54, 151.92, 136.76, 127.70, 123.21, 121.46, 121.21, 118.78, 118.75, 118.67, 115.64, 112.10, 111.90, 111.71, 56.14, 40.45, 25.44.

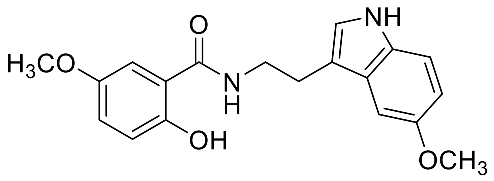

***N*-(2-(5-methoxy-1H-indol-3-yl)ethyl)-2-hydroxy-5-methoxybenzamide(5p).**[10] white solid, isolated yield 84.9%, m.p. 141℃, Purity＞99.5%. ^{1}H NMR (400 MHz, DMSO-d6) δ 12.20 (s, 1H), 10.70 (s, 1H), 8.96 (t, J = 7.3 Hz, 1H), 7.43 (s, 1H), 7.26 (d, J = 7.2 Hz, 1H), 7.18 (d, J = 2.2 Hz, 1H), 7.09 (d, J = 2.7 Hz, 1H), 7.03 (dd, J = 9.0, 3.0 Hz, 1H), 6.86 (d, J = 7.5 Hz, 1H), 6.74 (dd, J = 8.8,

2.5 Hz, 1H), 3.73 (s, 6H), 3.61 (q, J = 6.4, 5.5 Hz, 2H), 2.98 (t, J = 6.2 Hz, 2H). ^{13}C NMR (101 MHz, DMSO-d6) δ 169.09, 154.56, 153.50, 151.92, 131.87, 128.06, 123.89, 121.20, 118.66, 115.61, 112.53, 111.98, 111.72, 111.61, 100.57, 56.13, 55.67, 40.50, 25.40.

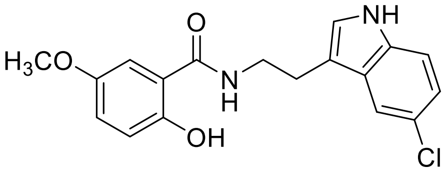

***N*-(2-(5-chloro-1H-indol-3-yl)ethyl)-2-hydroxy-5-methoxybenzamide(5q).[11]** white solid, isolated yield 77.2%, m.p. 147℃ Purity＞99.5%. ^{1}H NMR (400 MHz, DMSO-d6) δ 12.15 (s, 1H), 11.09 (s, 1H), 8.97 (t, J = 9.0 Hz, 1H), 7.66 (d, J = 5.7 Hz, 1H), 7.47 – 7.38 (m, 2H), 7.37 (d, J = 10.0 Hz, 1H), 7.29 (d, J = 5.5 Hz, 1H), 7.02 (dd, J = 9.0, 3.0 Hz, 2H), 6.85 (d, J = 5.8 Hz, 1H), 3.74 (s, 3H), 3.59 (q, J = 9.3, 7.8 Hz, 2H), 2.98 (t, J = 8.9 Hz, 2H). ^{13}C NMR (101 MHz, DMSO-d6) δ 169.03, 154.49, 151.94, 135.19, 128.89, 125.21, 123.56, 121.37, 121.21, 118.67, 118.10, 115.62, 113.41, 112.15, 111.67, 56.13, 40.40, 25.20.

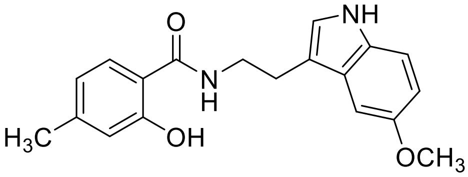

***N*-(2-(5-methoxy-1H-indol-3-yl)ethyl)-2-hydroxy-4-methylbenzamide(5r).[8]** white solid, isolated yield 72.8%, m.p. 125℃ Purity＞99.5%. ^{1}H NMR (400 MHz, DMSO-d6) δ 12.80 (s, 1H), 10.70 (s, 1H), 8.91 (t, J = 5.7 Hz, 1H), 7.76 (d, J = 8.1 Hz, 1H), 7.26 (d, J = 8.7 Hz, 1H), 7.17 (d, J = 2.4 Hz, 1H), 7.08 (d, J = 2.4 Hz, 1H), 6.78 – 6.67 (m, 3H), 3.74 (s, 3H), 3.59 (q, J = 6.8 Hz, 2H), 2.97 (t, J = 7.4 Hz, 2H), 2.28 (s, 3H). ^{13}C NMR (101 MHz, DMSO-d6) δ 169.65, 160.90, 153.49, 144.54, 131.87, 128.07, 127.84, 123.85, 119.99, 118.03, 112.92, 112.52, 111.99, 111.62, 100.55, 55.67, 30.83, 25.41, 21.54.

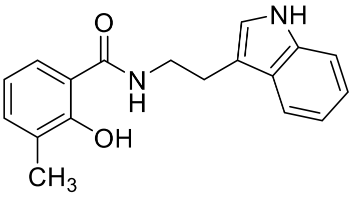

***N*-(2-(1H-indol-3-yl)ethyl)-2-hydroxy-3-methylbenzamide(5s).[8]** white solid, isolated yield 87.1%, m.p. 170℃ Purity＞99.5%. ^{1}H NMR (400 MHz, DMSO-d6) δ 13.34 (s, 1H), 10.85 (s, 1H), 9.05 (d, J = 9.0 Hz, 1H), 7.68 (d, J = 7.7 Hz, 1H), 7.60 (s, 1H), 7.35 (d, J = 8.0 Hz, 1H), 7.29 (d, J = 7.3 Hz, 1H), 7.20 (s, 1H), 7.09 (d, J = 7.2 Hz, 1H), 6.99 (t, J = 7.4 Hz, 1H), 6.78 (s, 1H), 3.59 (q, J = 9.9, 7.7 Hz, 2H), 2.99 (t, J = 7.4 Hz, 2H), 2.17 (s, 3H). ^{13}C NMR (101 MHz, DMSO-d6) δ 170.65, 159.95, 136.76, 134.90, 127.72, 126.47, 124.97, 123.19, 121.45, 118.78, 118.73, 118.11, 114.04, 112.09, 111.90, 40.44, 25.36, 15.97.

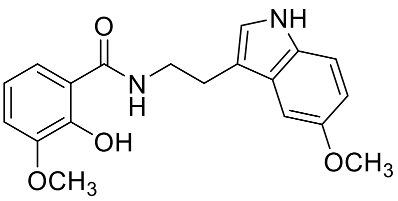

N-(2-(5-methoxy-1H-indol-3-yl)ethyl)-2-hydroxy-3-methoxybenzamide(5t).[11] white solid, isolated yield 79.9%, m.p. 151℃ Purity＞99.5%. ^{1}H NMR (400 MHz, DMSO-d6) δ 12.95 (s, 1H), 10.68 (s, 1H), 8.98 (t, J = 4.8 Hz, 1H), 7.44 (d, J = 6.8 Hz, 1H), 7.25 (d, J = 8.7 Hz, 1H), 7.16 (d, J = 2.0 Hz, 1H), 7.09 (dd, J = 9.1, 2.1 Hz, 2H), 6.81 (t, J = 8.0 Hz, 1H), 6.73 (dd, J = 8.7, 1.9 Hz, 1H), 3.79 (s, 3H), 3.73 (s, 3H), 3.58 (q, J = 6.4, 5.8 Hz, 2H), 2.97 (t, J = 6.8 Hz, 2H). ^{13}C NMR (101 MHz, DMSO-d6) δ 169.96, 153.49, 151.44, 148.96, 131.85, 128.05, 123.87, 119.03, 118.18, 115.77, 115.39, 112.53, 111.93, 111.61, 100.55, 56.20, 55.68, 40.48, 25.34.

***N*-(cyclohexylmethyl)-2-hydroxybenzamide (3a)**

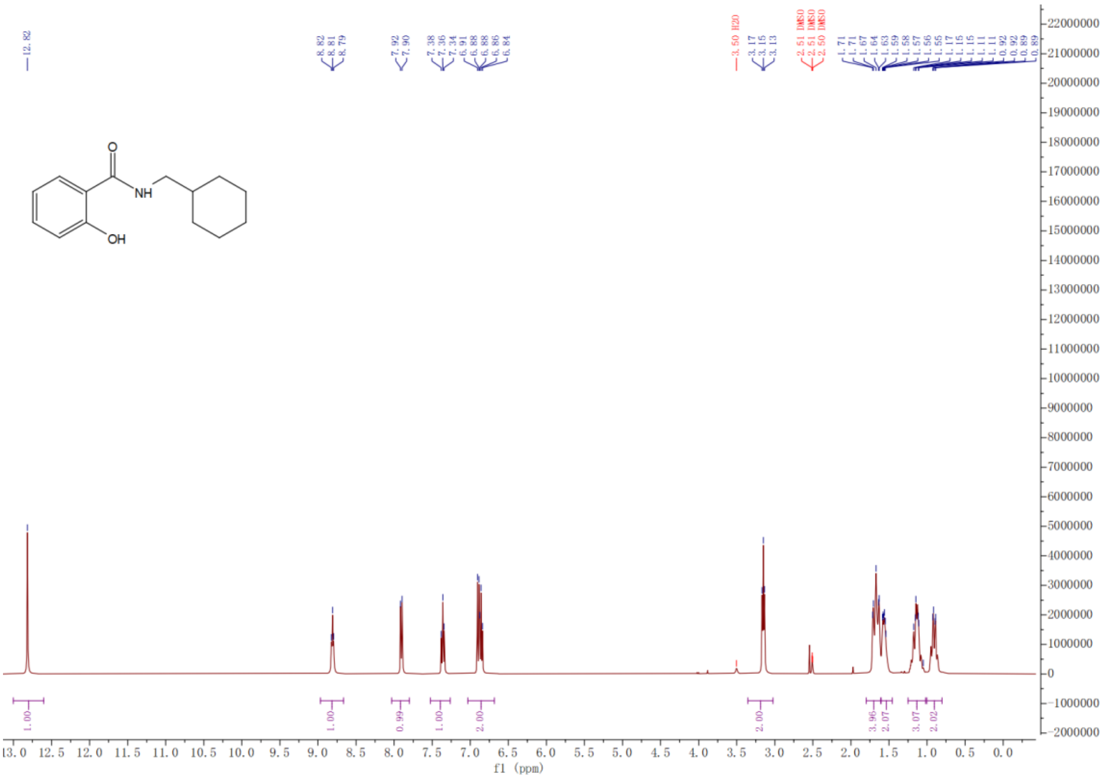

5-Chloro-*N*-(cyclohexylmethyl)-2-hydroxybenzamide (3b):

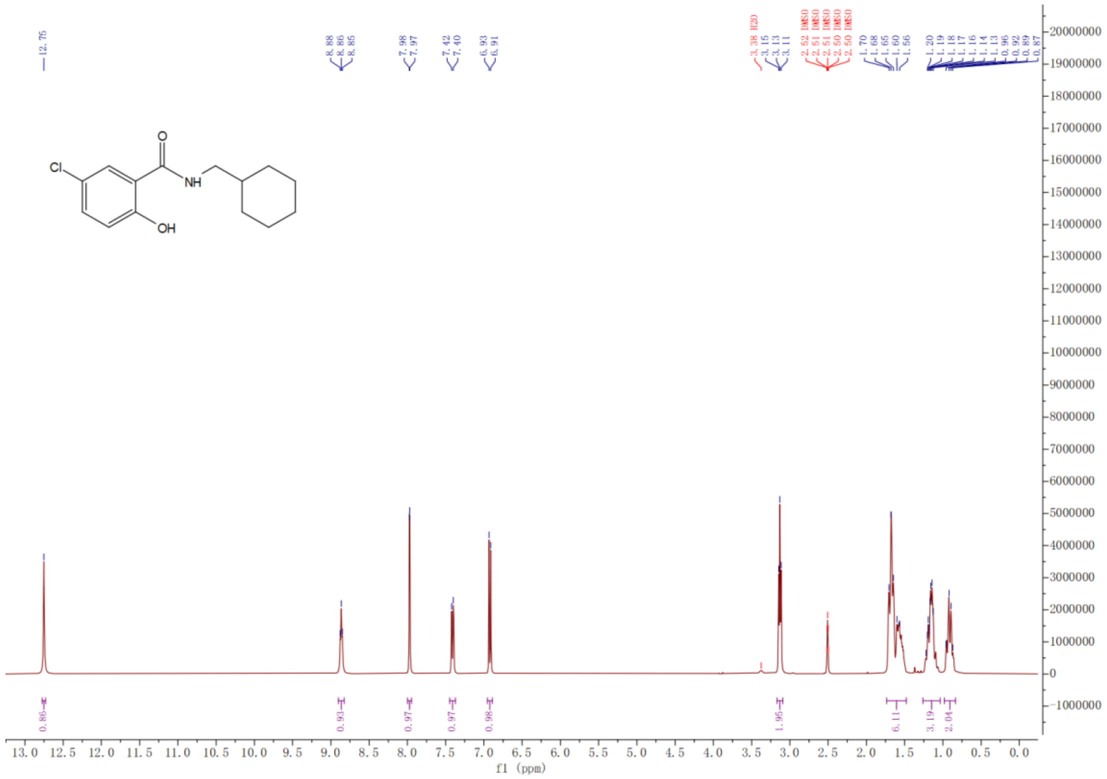

5-Chloro-*N*-cyclopentyl-2-hydroxybenzamide(3c):

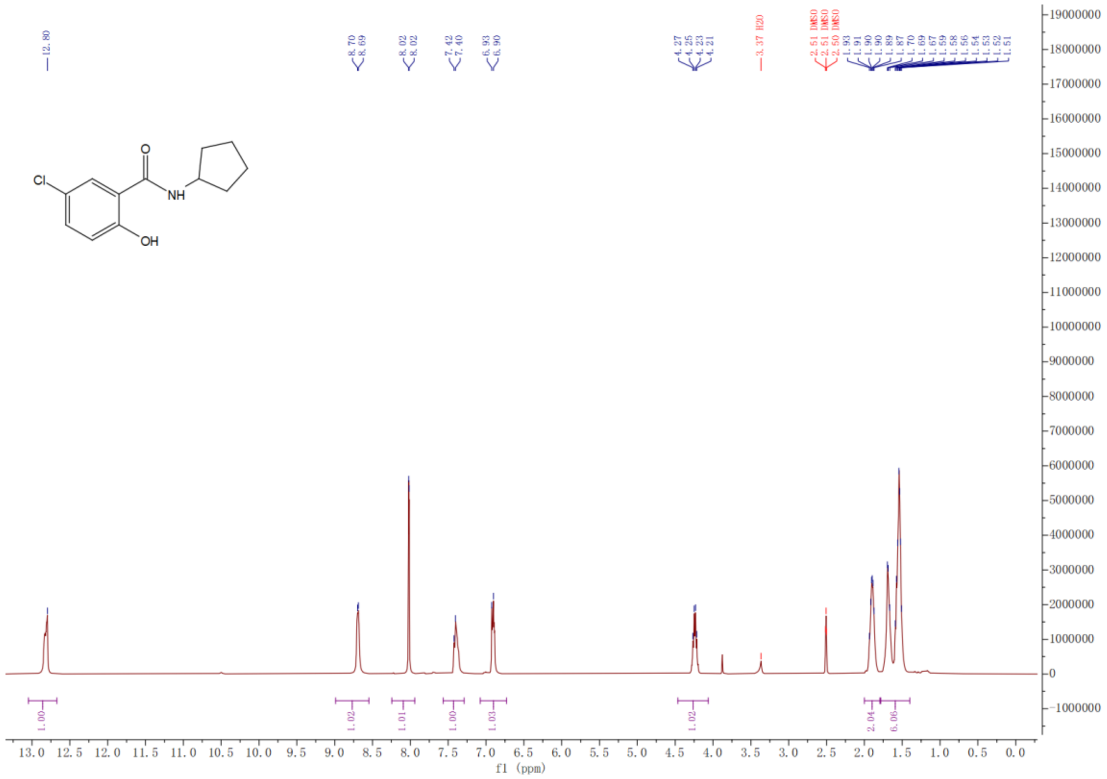

***N*-cyclopentyl-2-hydroxybenzamide (3d)**:

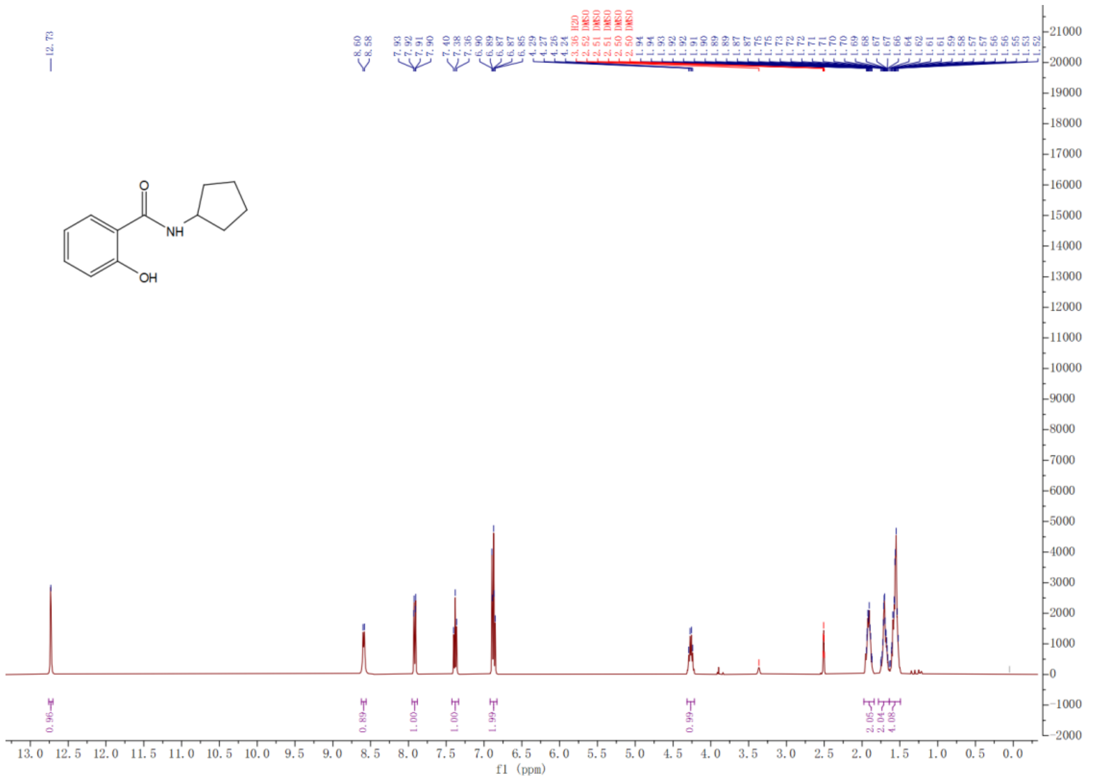

5-Chloro-*N*-cyclohexyl-2-hydroxybenzamide (3e):

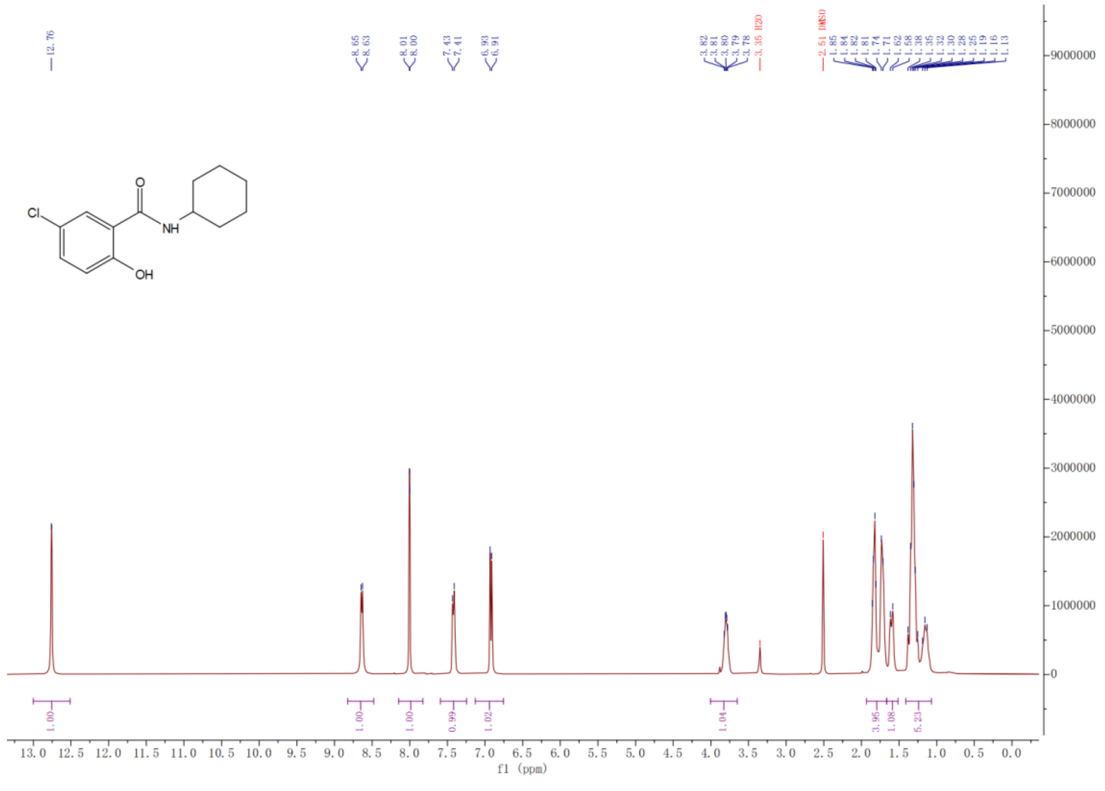

***N*-cyclohexyl-2-hydroxybenzamide (3f):**

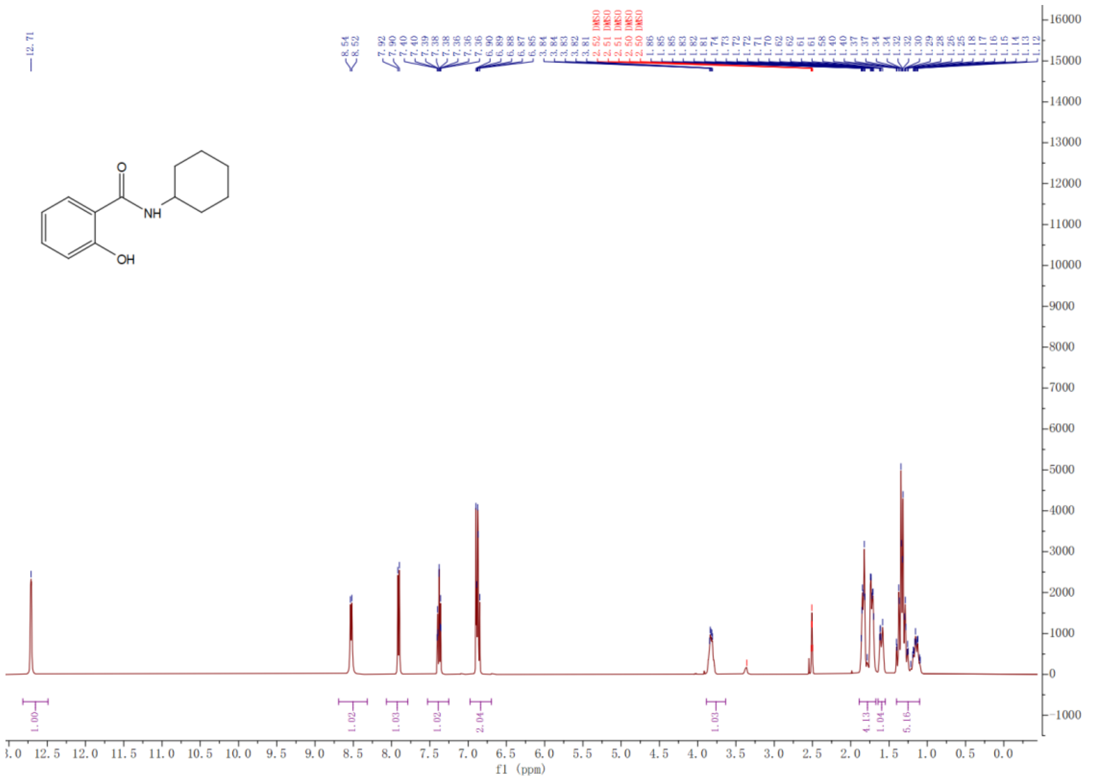

5-Chloro-2-hydroxy-*N*-(tetrahydro-2H-pyran-4-yl)benzamide (3g):

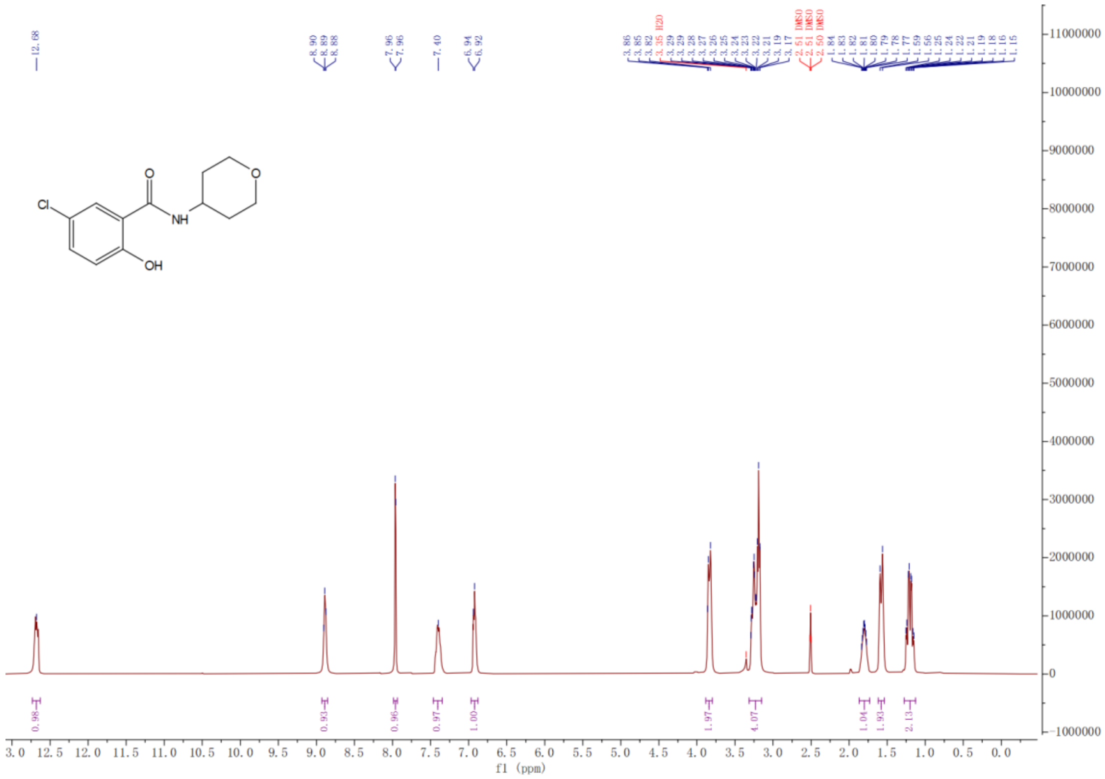

5-Chloro-*N*-hexyl-2-hydroxybenzamide (3h):

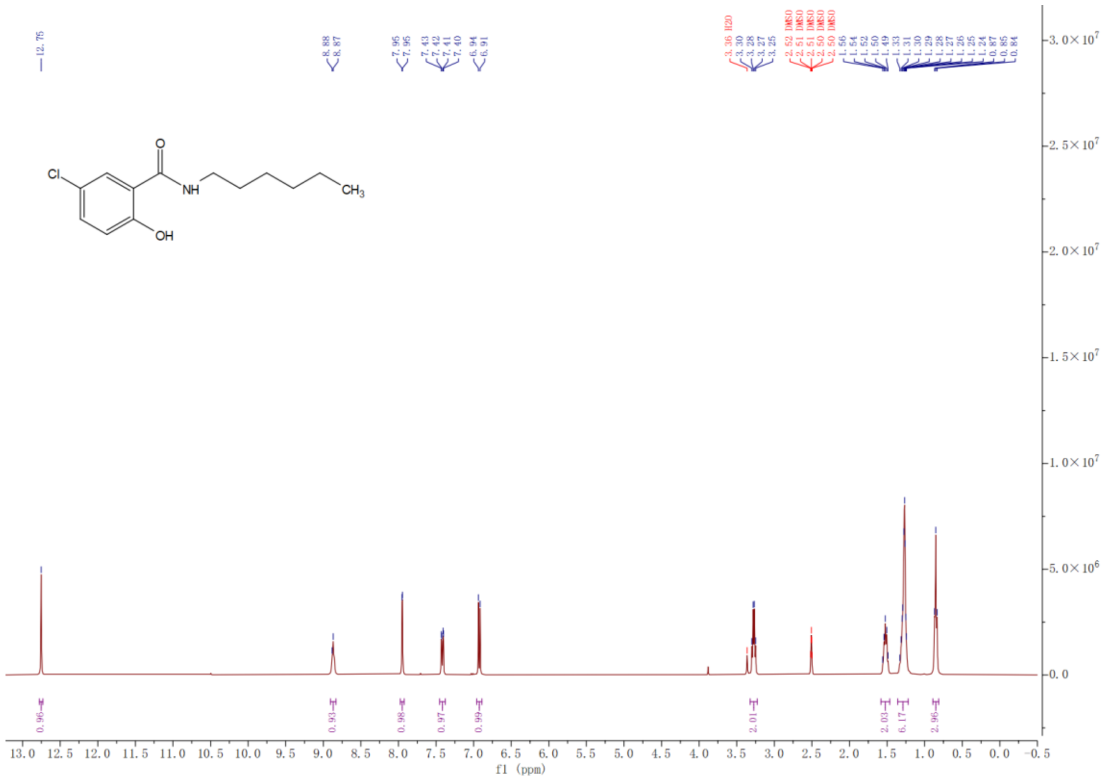

5-Bromo-*N*-hexyl-2-hydroxybenzamide (3i):

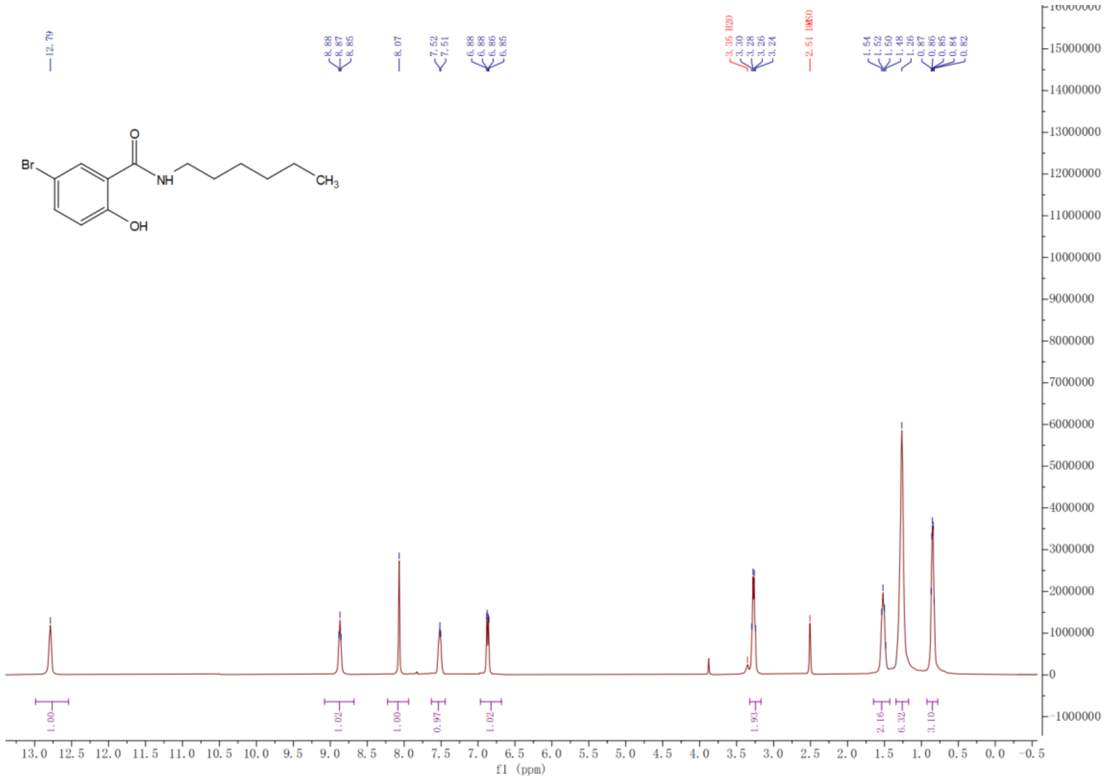

2-Hydroxy-N-(phenylmethyl)benzamide (4a):

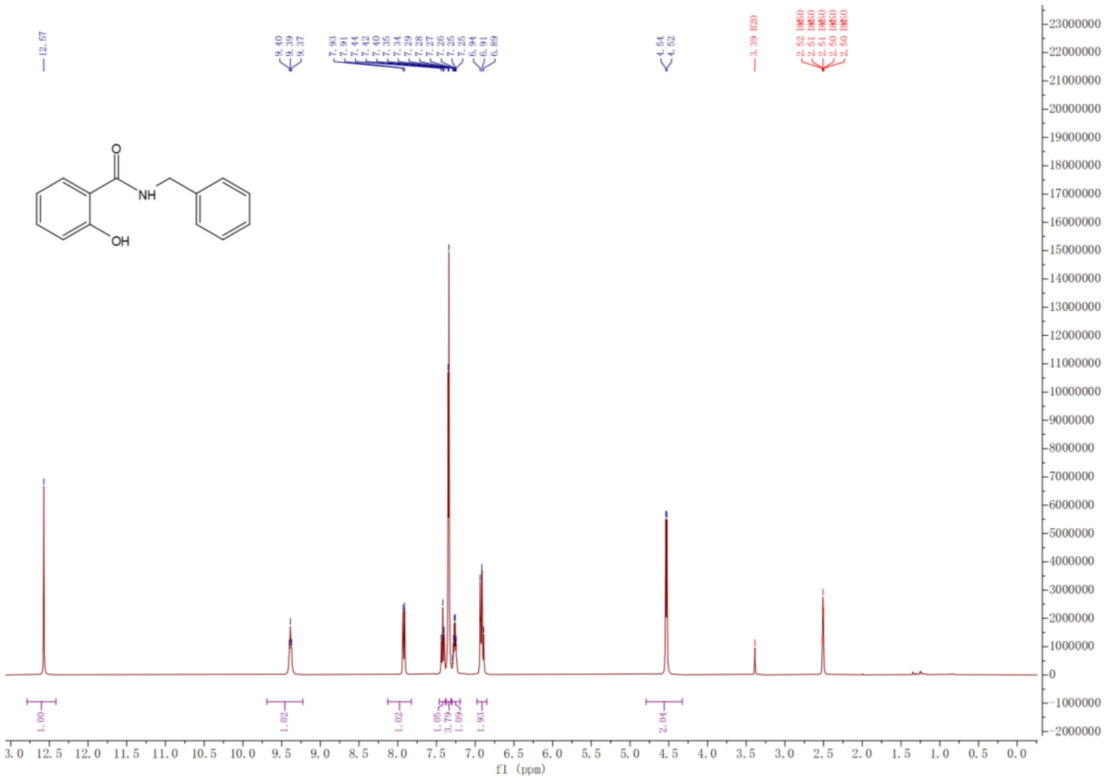

5-Chloro-*N*-[(4-chlorophenyl)methyl]-2-hydroxybenzamide (4b):

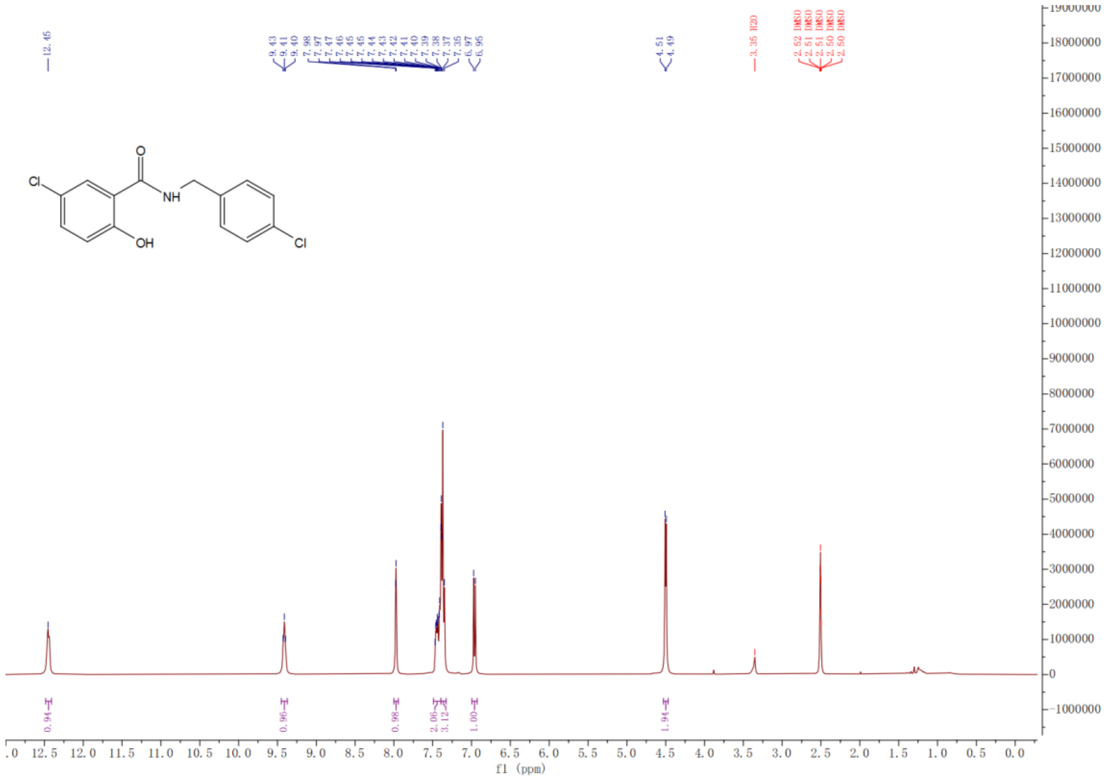

5-Chloro-*N*-[(4-methoxy)methyl]-2-hydroxybenzamide (4c):

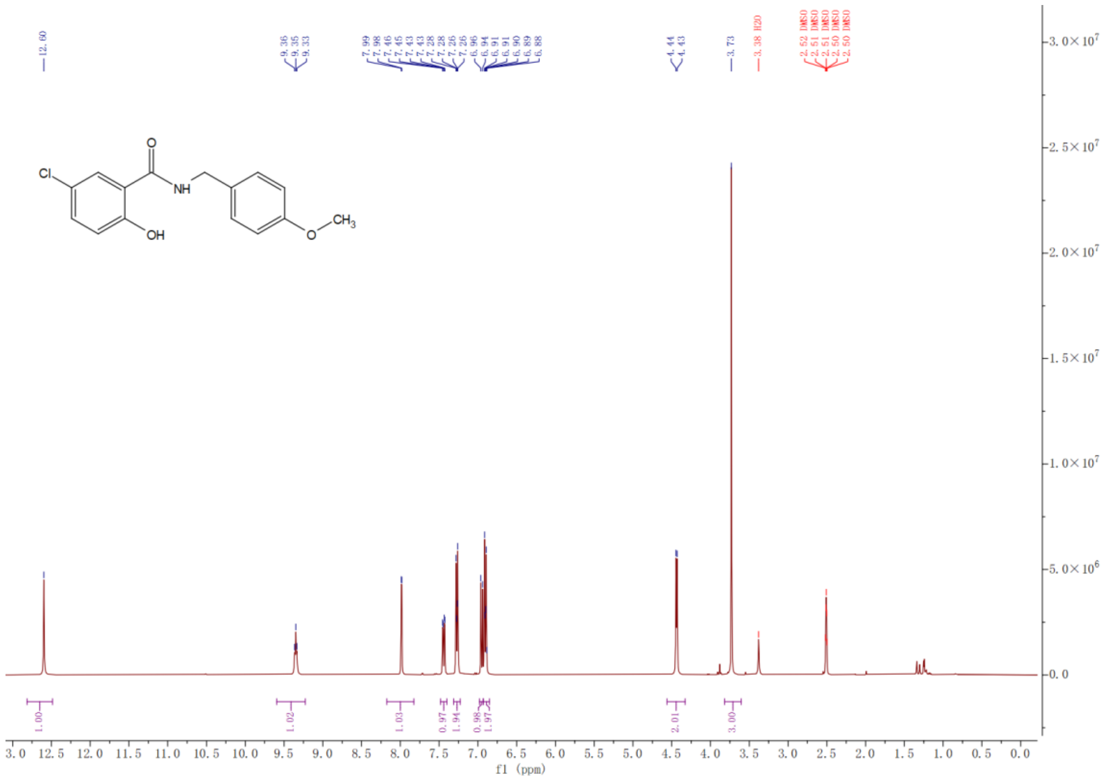

5-Chloro-2-hydroxy-*N*-(2-phenylethyl)benzamide (4d):

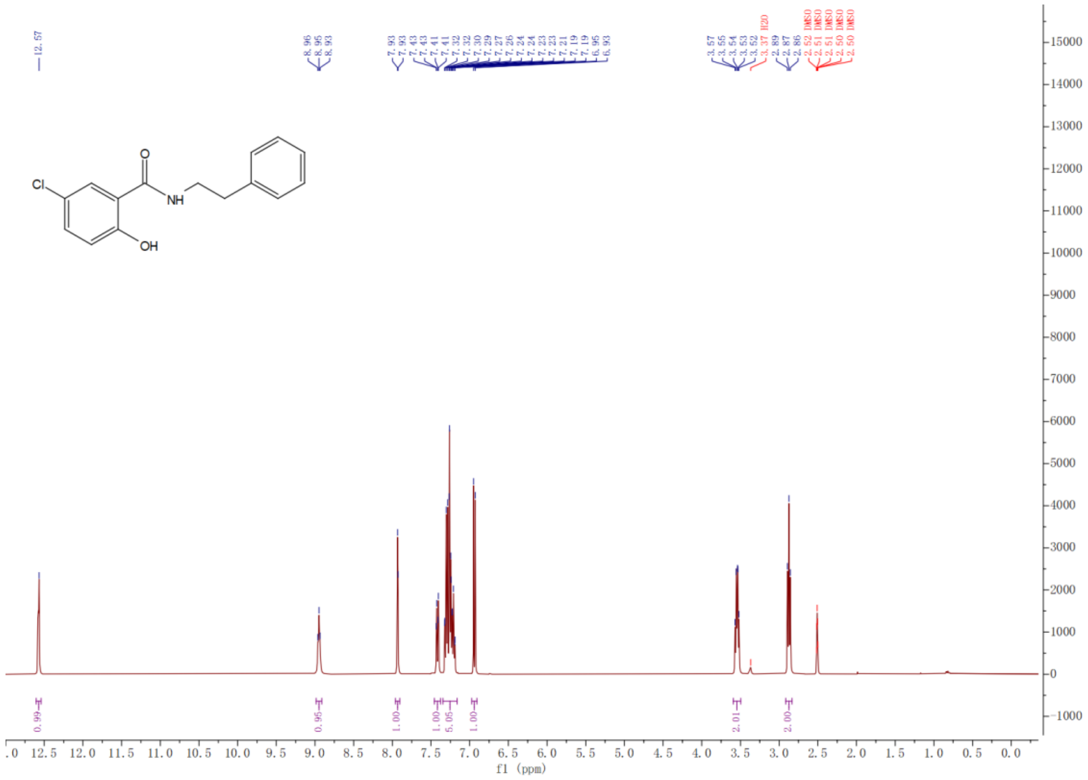

5-Chloro-N-(benzyl)-2-hydroxybenzamide(4e):

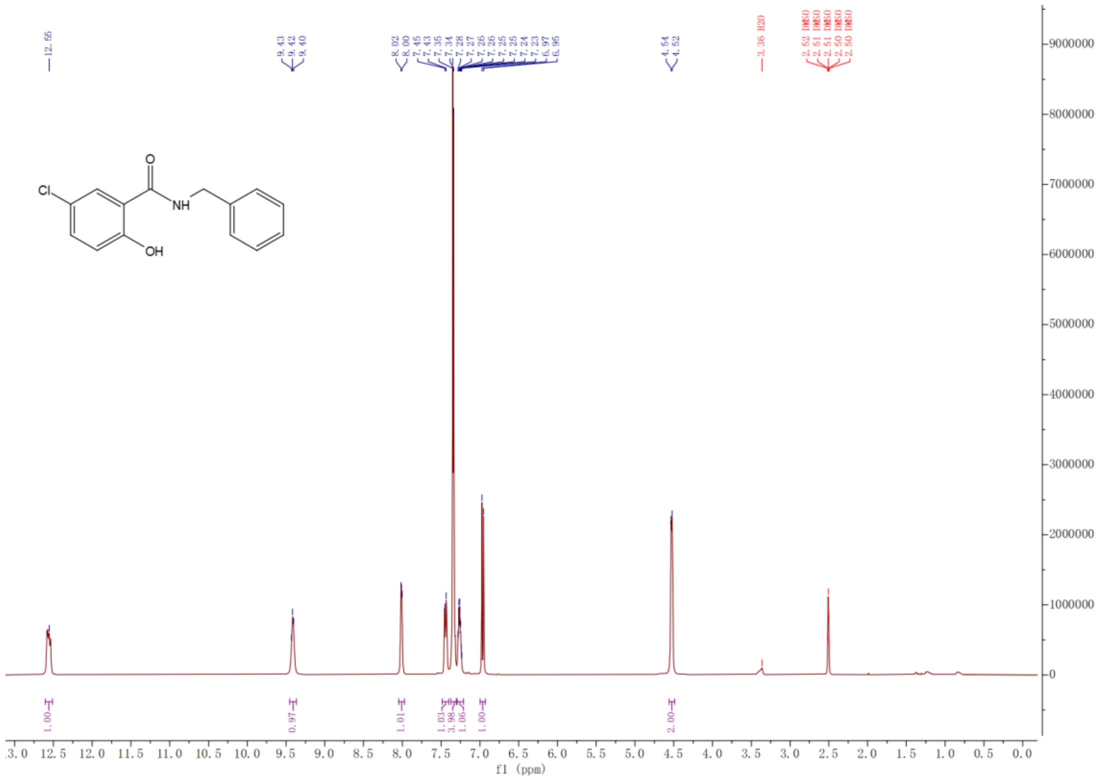

***N*-(2-(1H-indol-3-yl)ethyl)-2-hydroxybenzamide (5a)**:

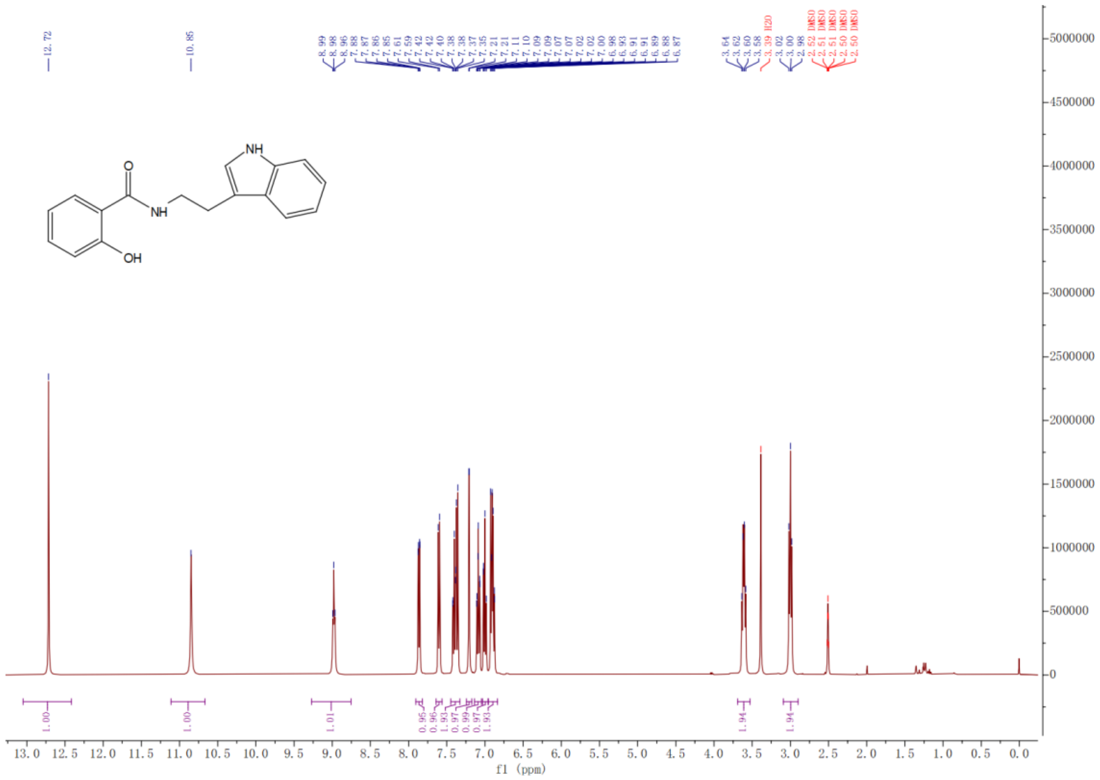

***N*-(2-(1H-indol-3-yl)ethyl)-5-bromo-2-hydroxybenzamide(5b)**:

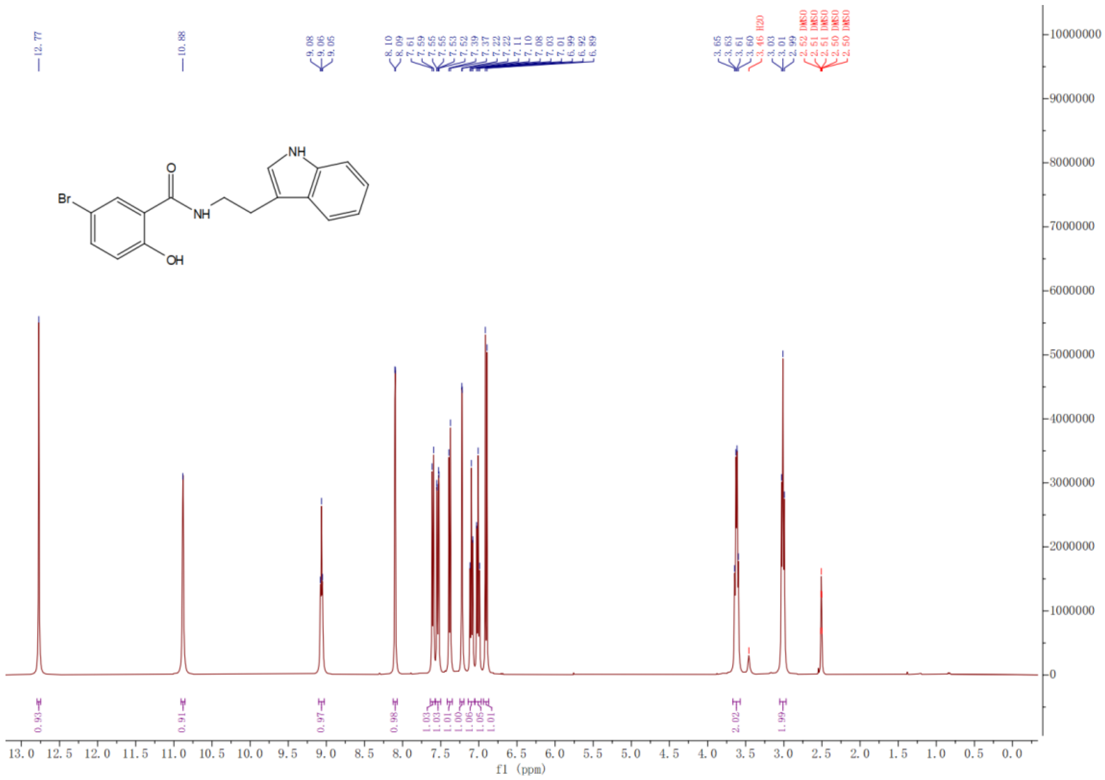

N-(2-(1H-indol-3-yl)ethyl)-2-hydroxy-4-methylbenzamide (5c):

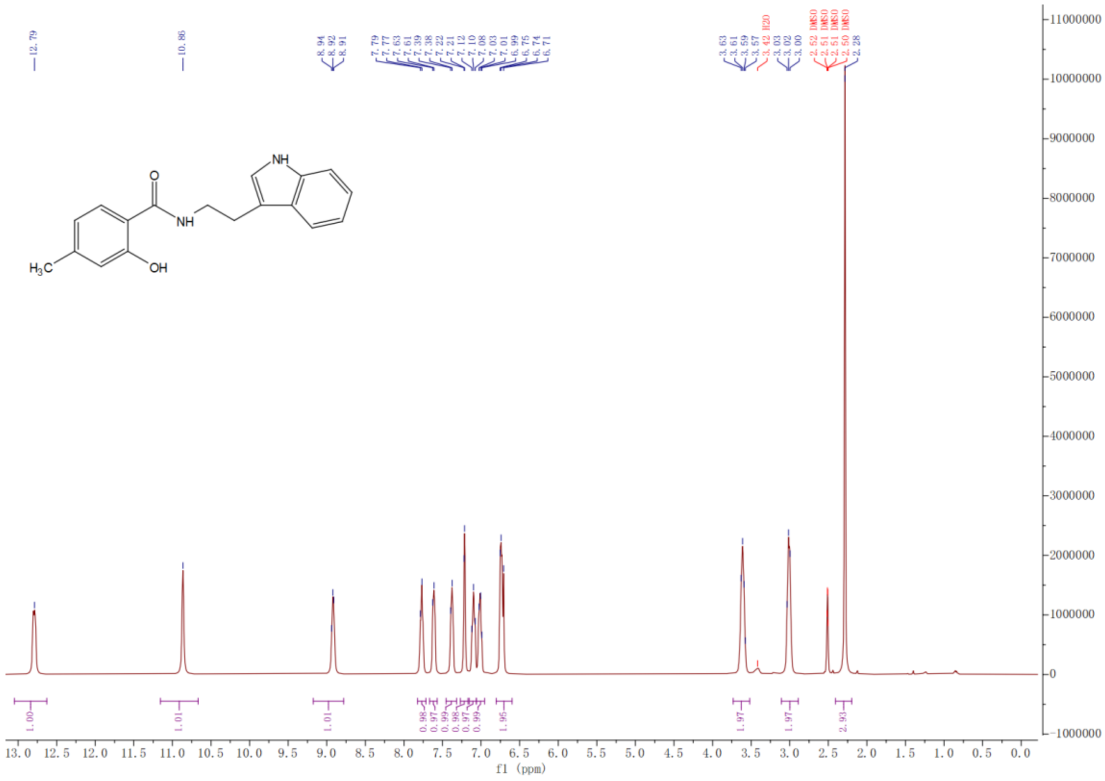

2-hydroxy-*N*-(2-(5-methoxy-1H-indol-3-yl)ethyl)benzamide (5d):

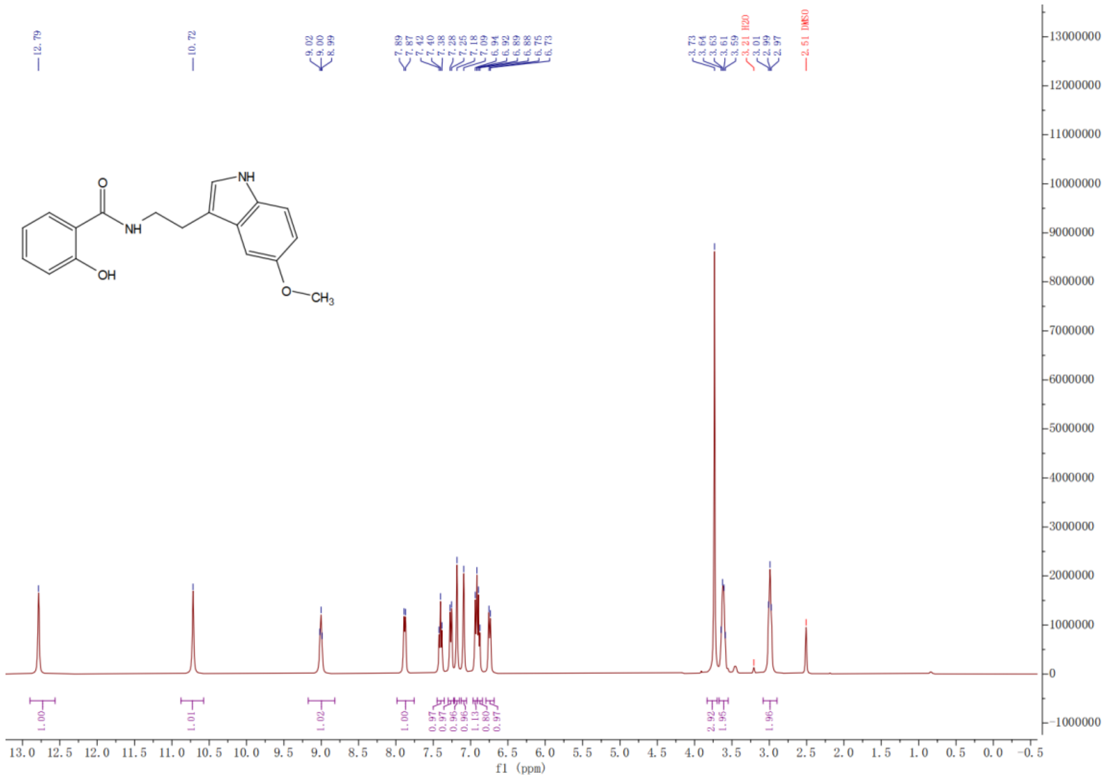

***N*-(2-(5-methoxy-1H-indol-3-yl)ethyl)-2-hydroxy-3-methylbenzamide(5e)**:

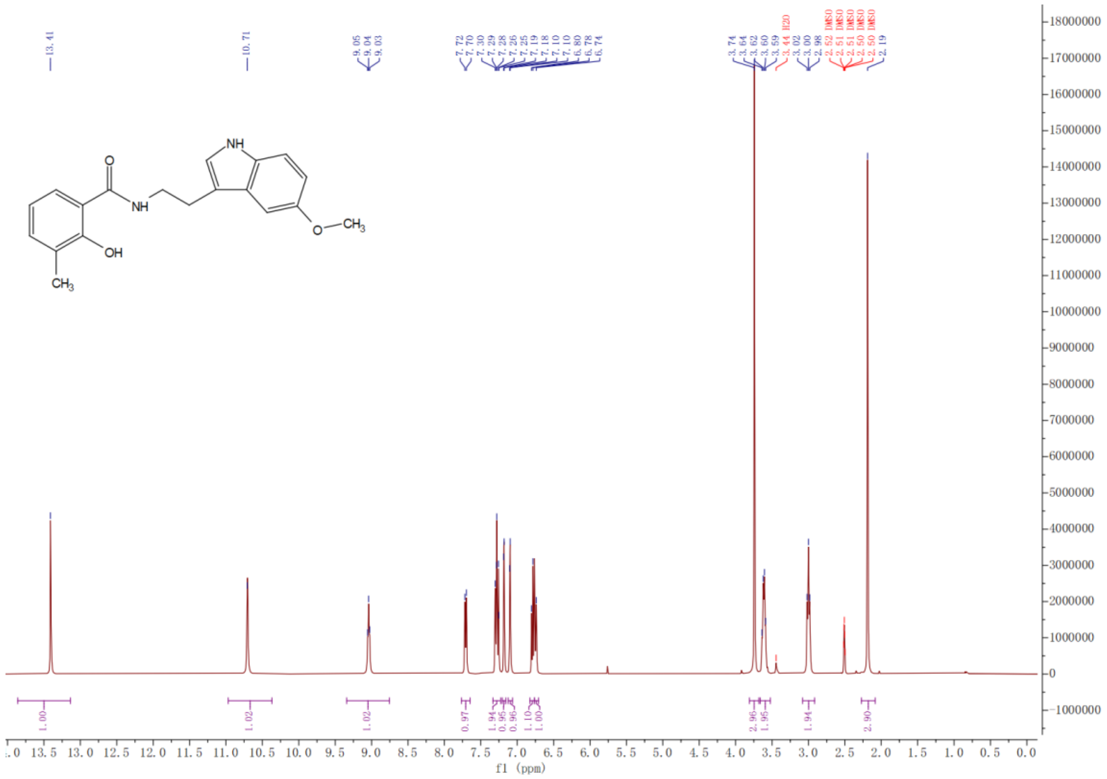

***N*-(2-(5-chloro-1H-indol-3-yl)ethyl)-2-hydroxy-4-methylbenzamide (5f)**:

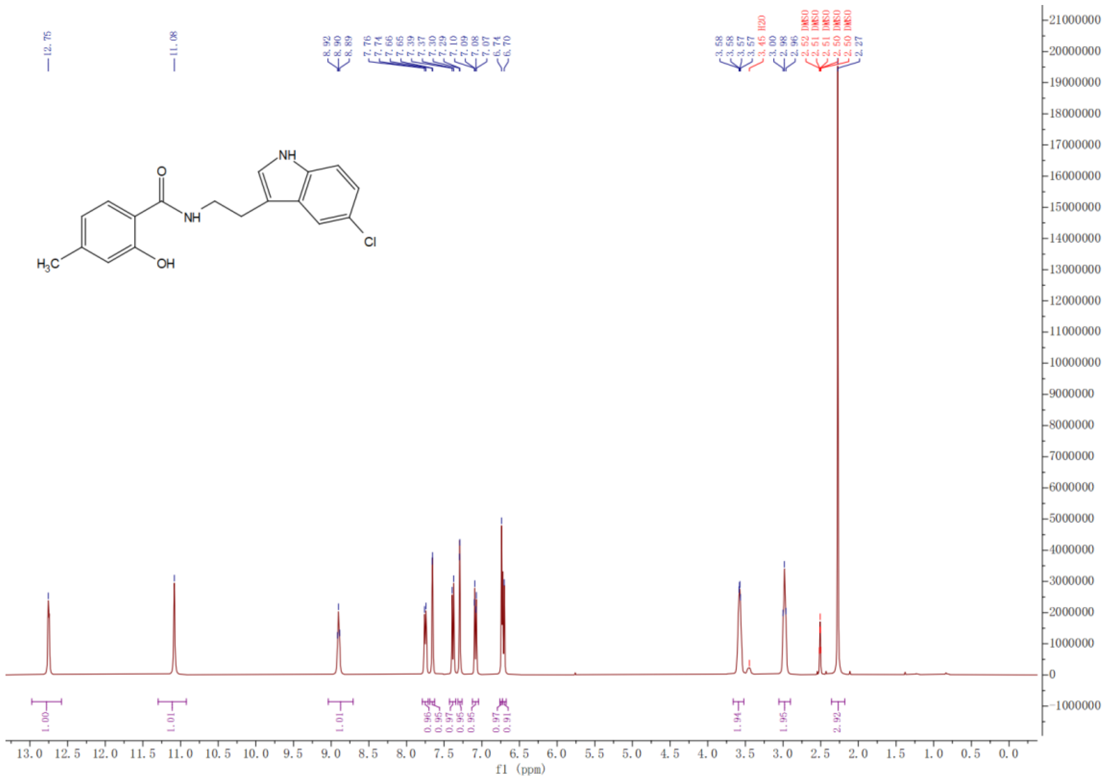

***N*-(2-(5-chloro-1H-indol-3-yl)ethyl)-2-hydroxybenzamide (5g):**

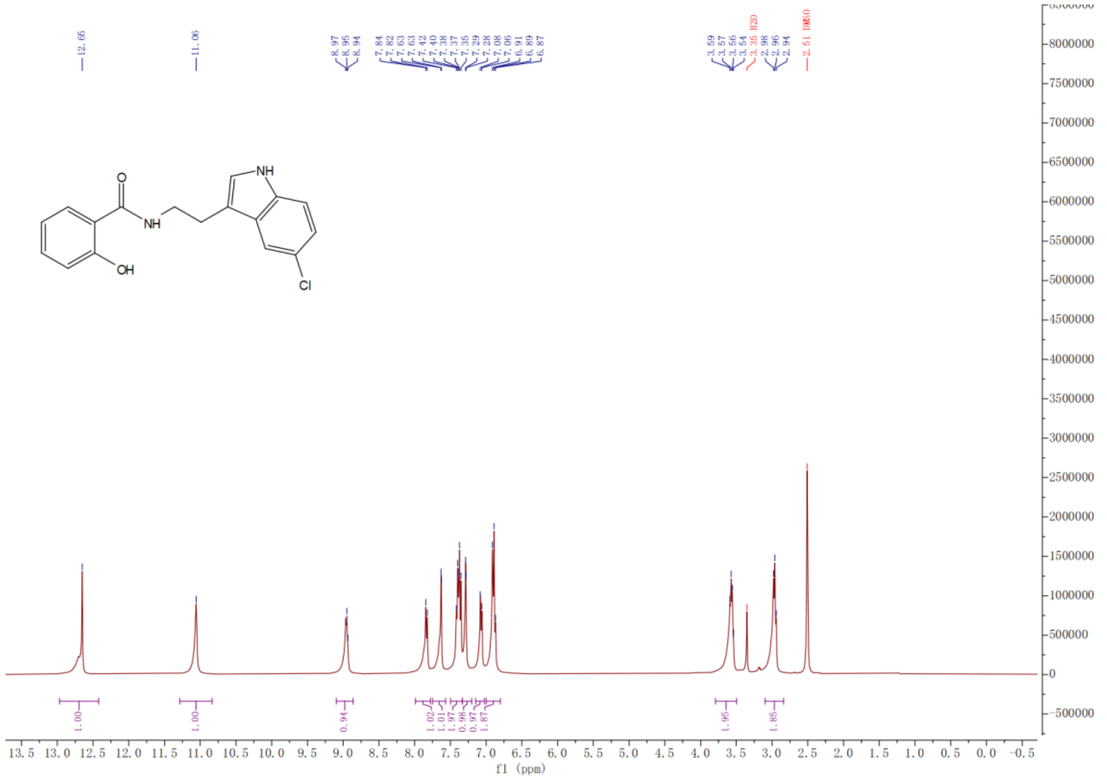

N-(2-(1H-indol-3-yl)ethyl)-2-hydroxy-5-methylbenzamide (5h):

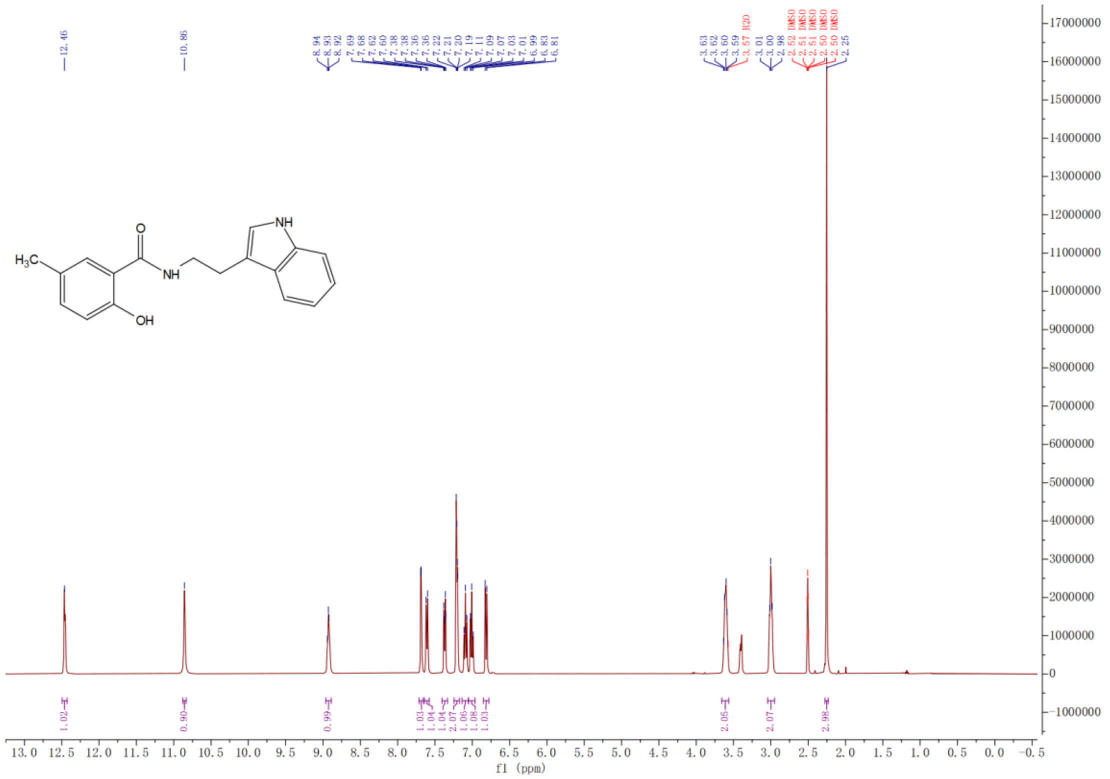

N-(2-(1H-indol-3-yl)ethyl)-5-chloro-2-hydroxybenzamide (5i):

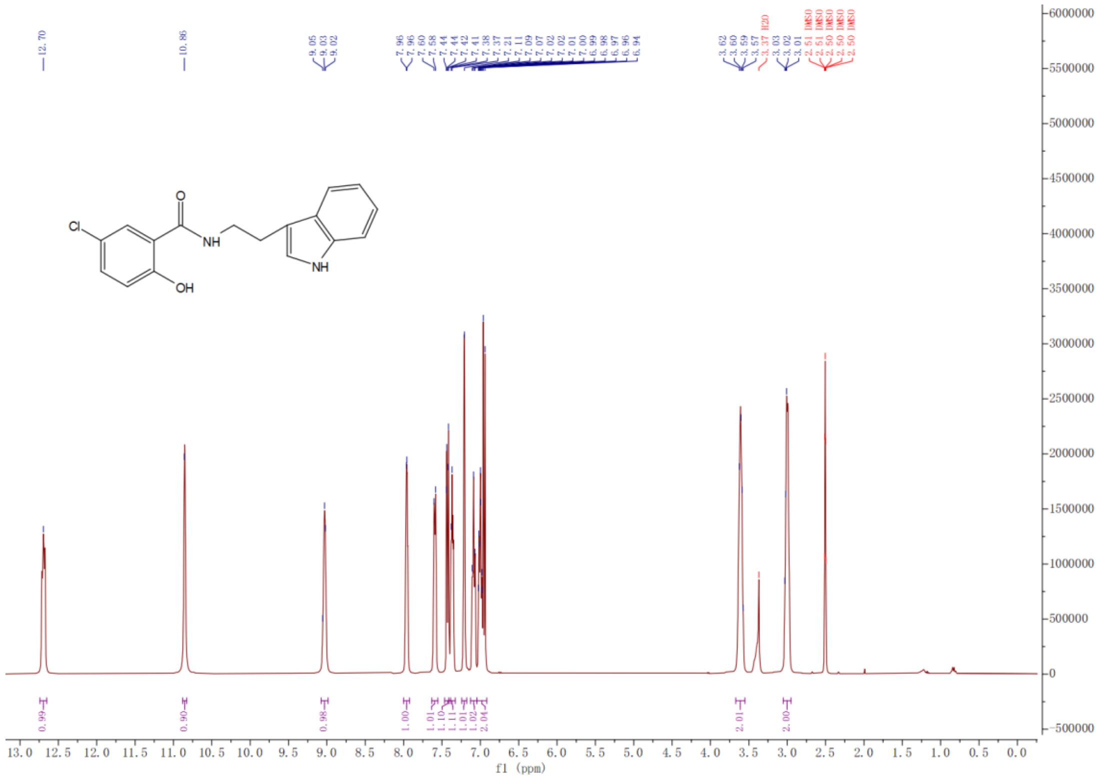

N-(2-(5-methoxy-1H-indol-3-yl)ethyl)-5-chloro-2-hydroxybenzamide (5j):

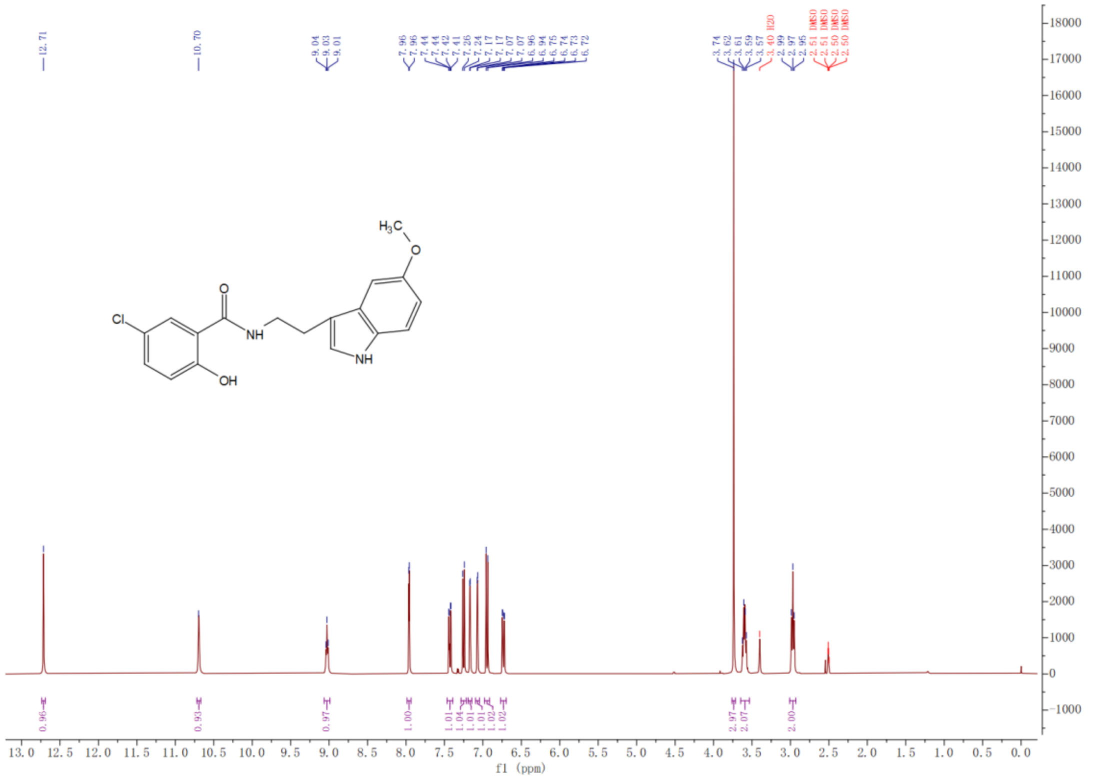

N-(2-(5-methoxy-1H-indol-3-yl)ethyl)-5-bromo-2-hydroxybenzamide (5k):

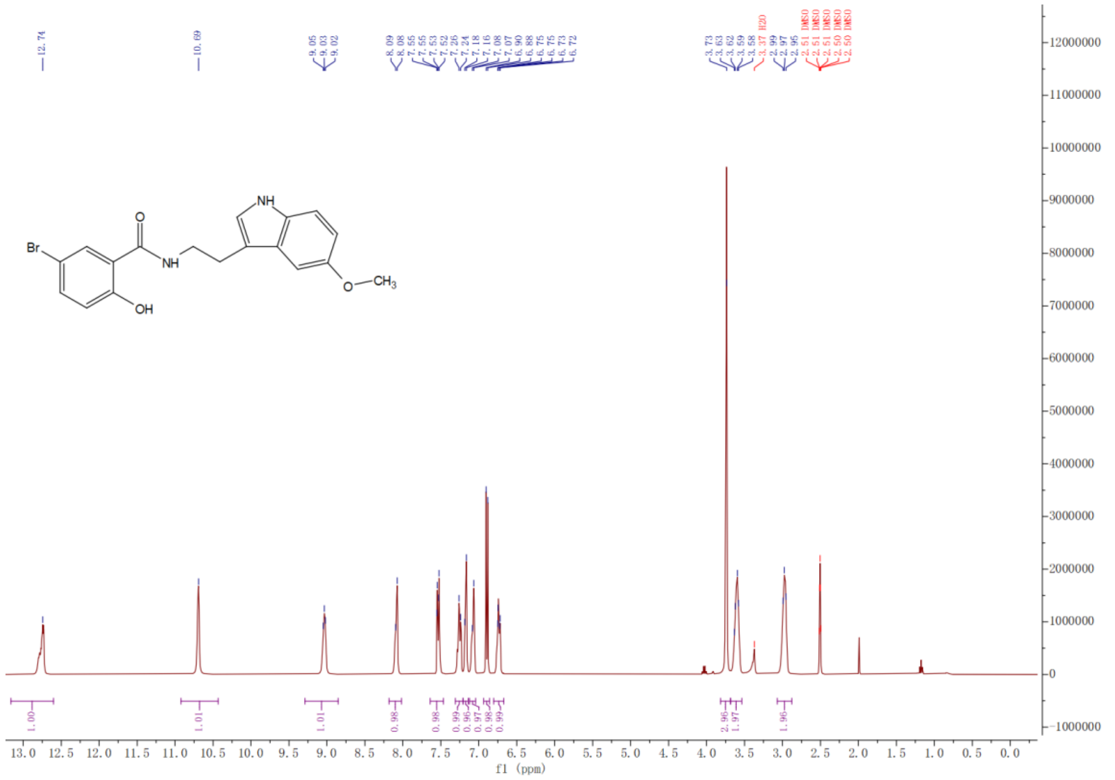

N-(2-(5-chloro-1H-indol-3-yl)ethyl)-5-bromo-2-hydroxybenzamide (5l):

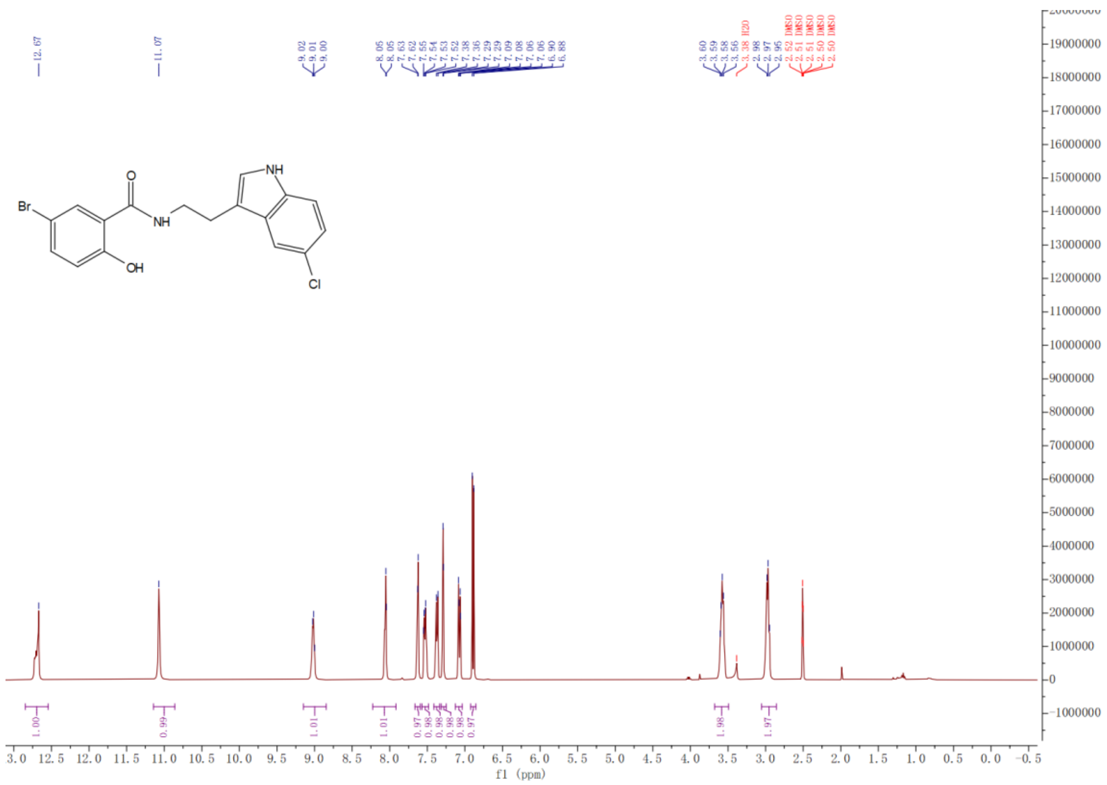

N-[2-(5-Chloro-1H-indol-3-yl)-ethyl]-2-hydroxy-3-methyl benzamide (5m):

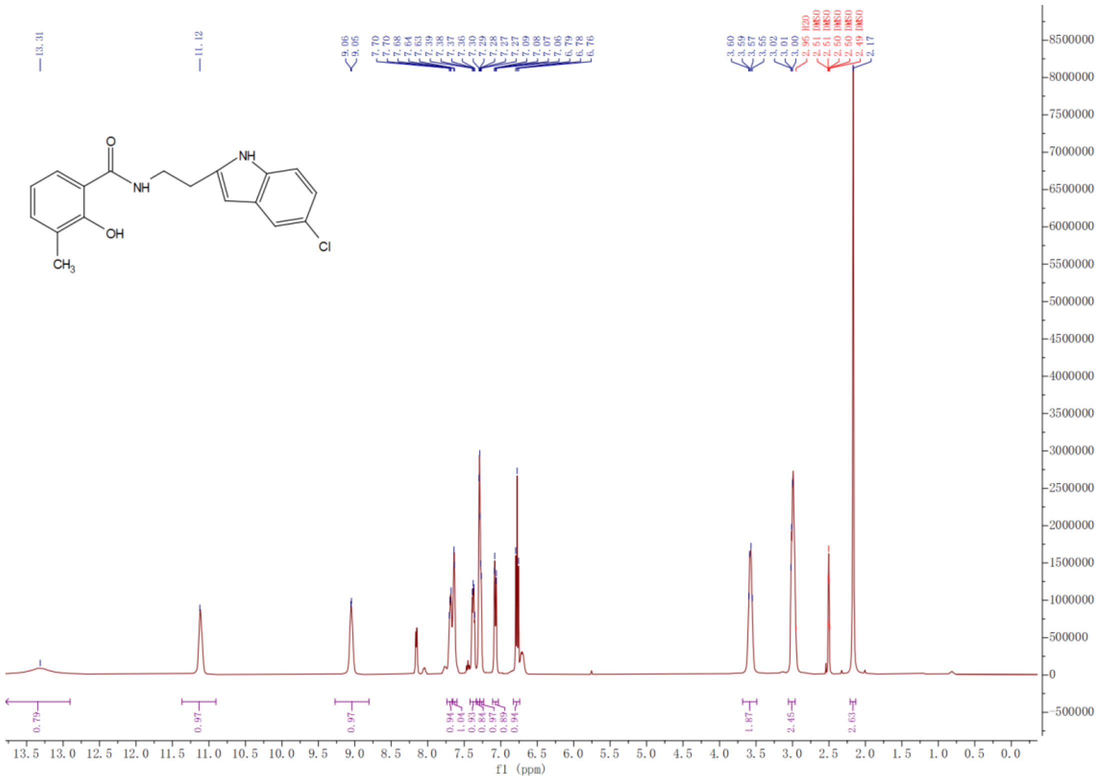

***N*-(2-(5-chloro-1H-indol-3-yl)ethyl)-2-hydroxy-5-methylbenzamide (5n):**

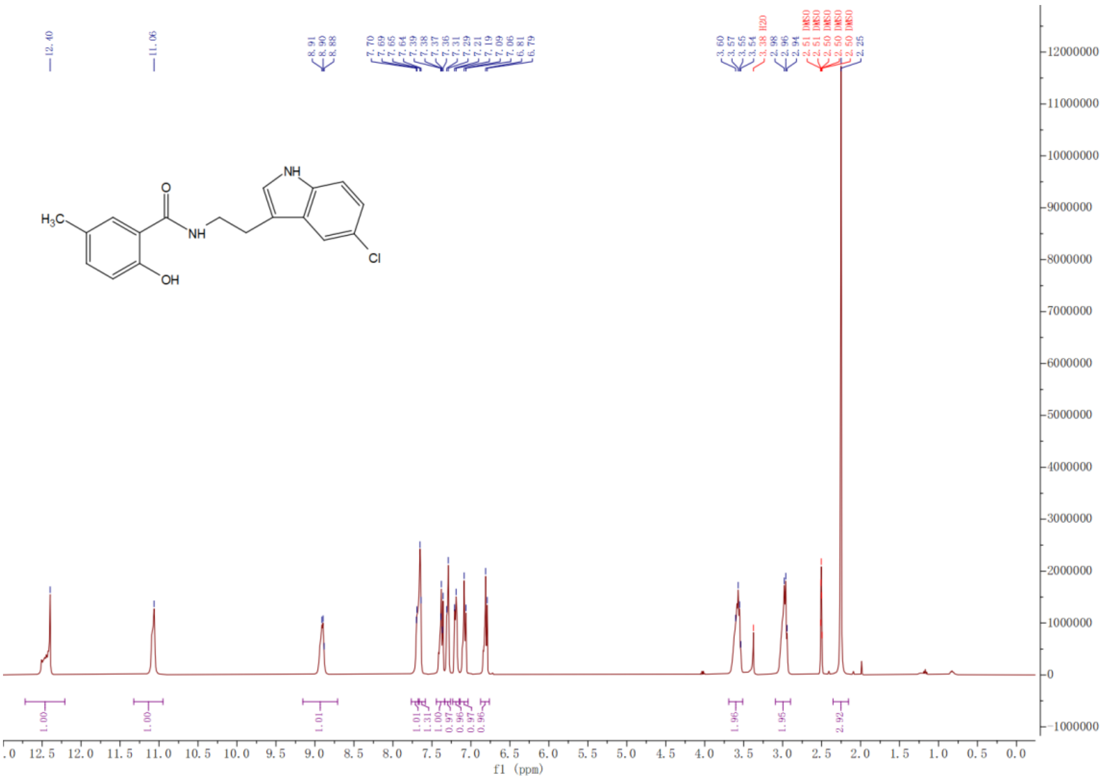

N-(2-(1H-indol-3-yl)ethyl)-2-hydroxy-5-methoxybenzamide (5o):

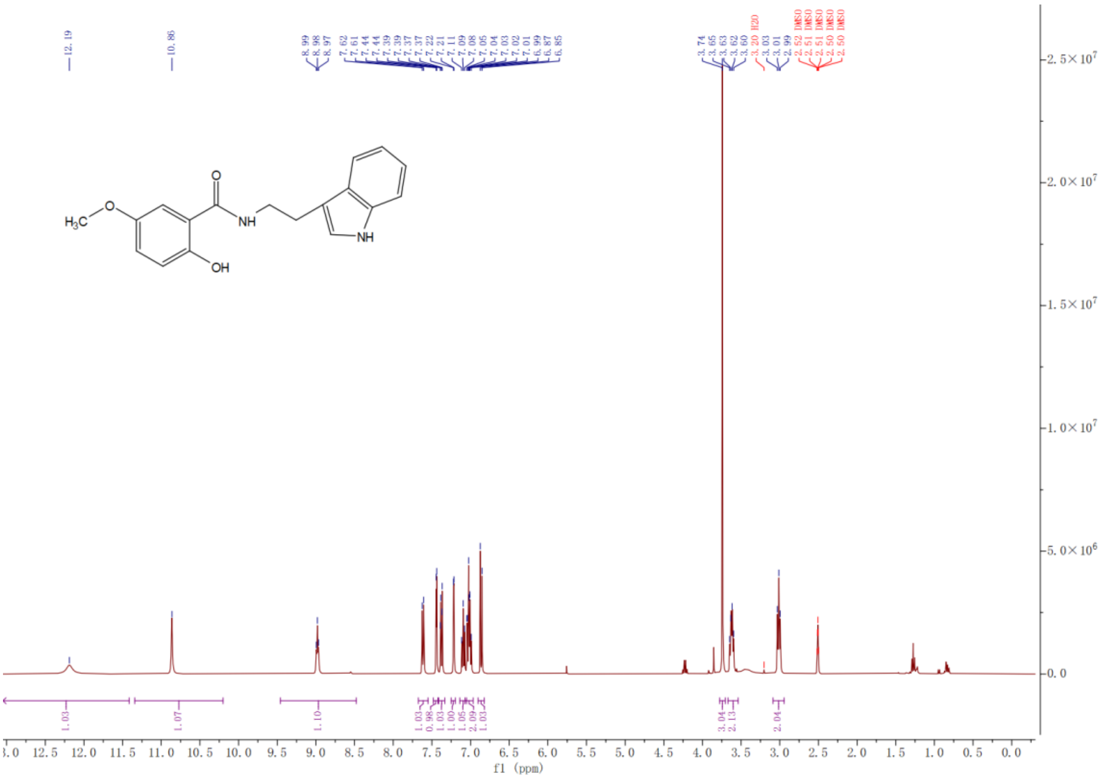

N-(2-(5-methoxy-1H-indol-3-yl)ethyl)-2-hydroxy-5-methoxybenzamide (5p):

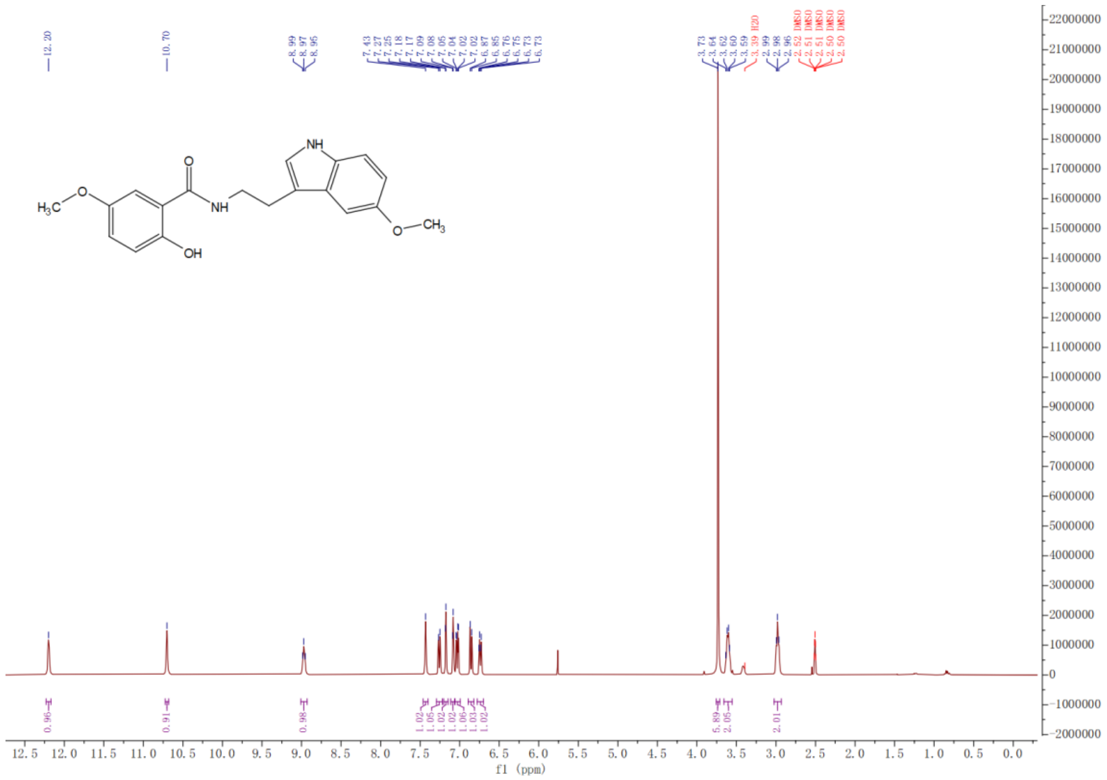

N-(2-(5-chloro-1H-indol-3-yl)ethyl)-2-hydroxy-5-methoxybenzamide (5q):

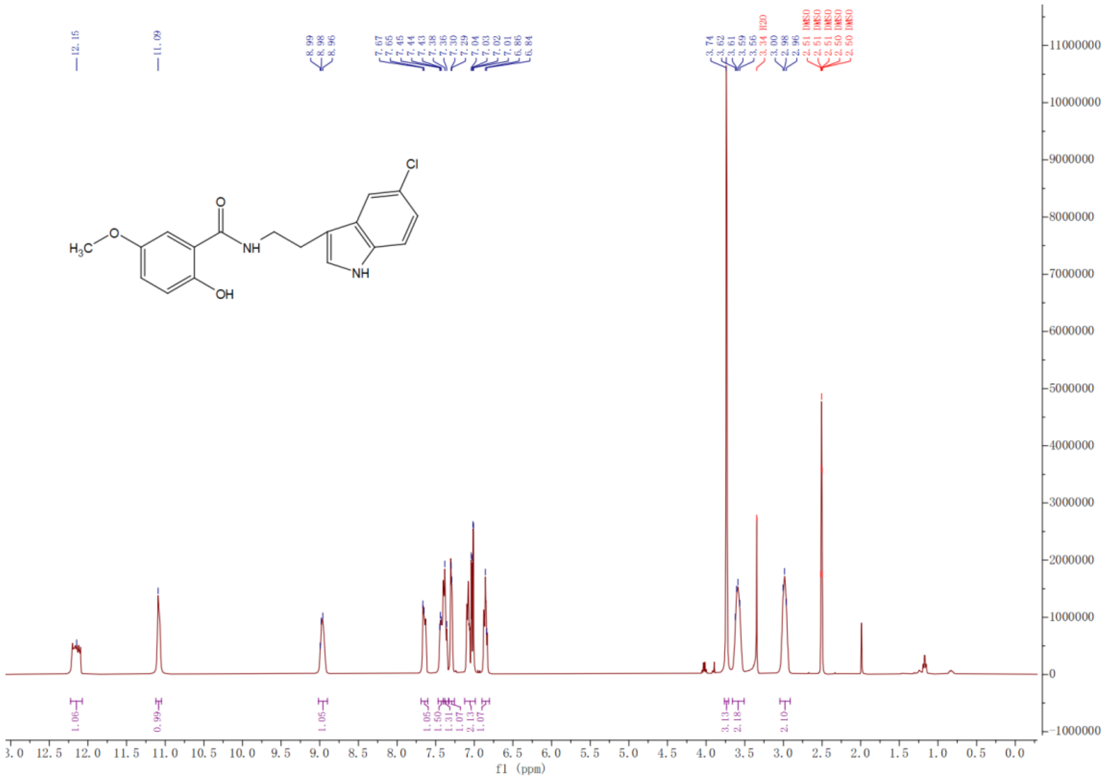

N-(2-(5-methoxy-1H-indol-3-yl)ethyl)-2-hydroxy-4-methylbenzamide (5r):

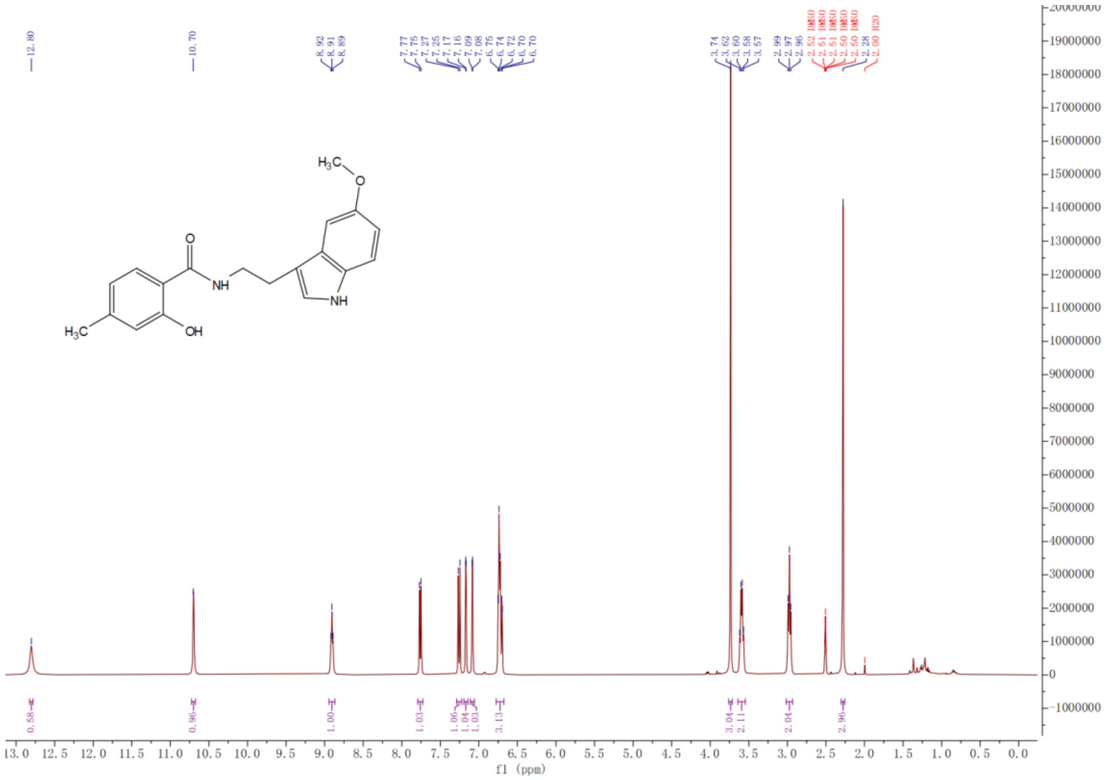

***N*-(2-(1H-indol-3-yl)ethyl)-2-hydroxy-3-methylbenzamide (5s):**

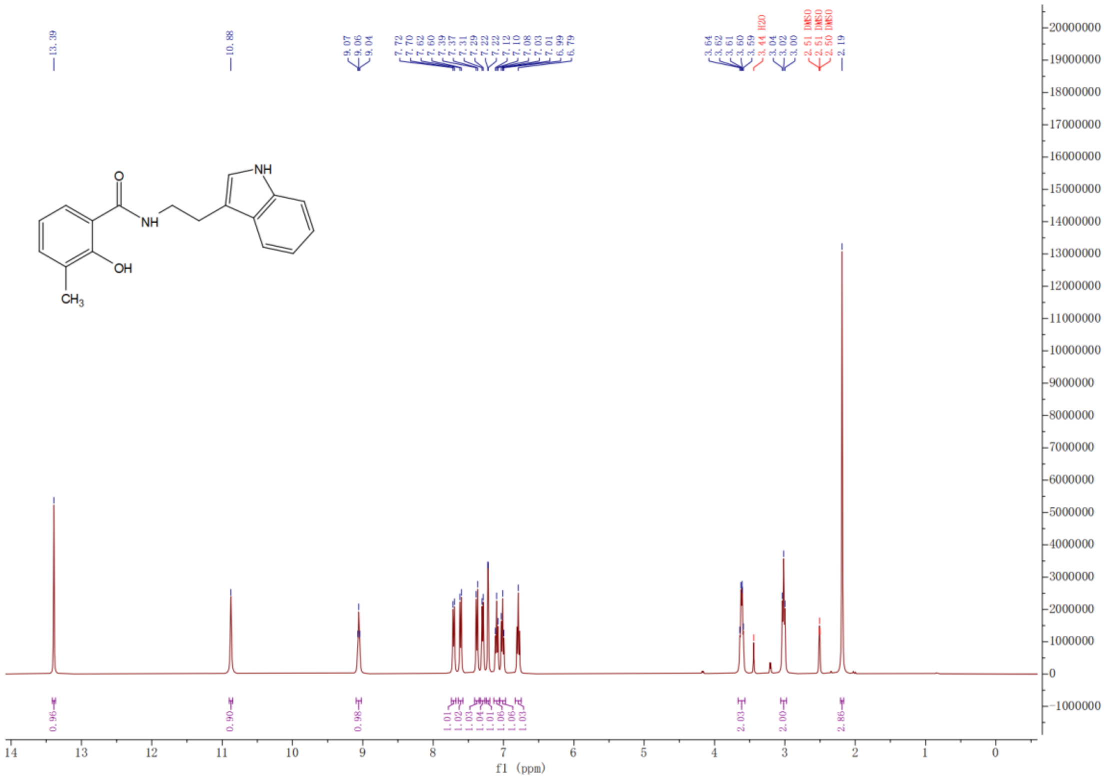

***N*-(2-(5-methoxy-1H-indol-3-yl)ethyl)-2-hydroxy-3-methoxybenzamide(5t):**

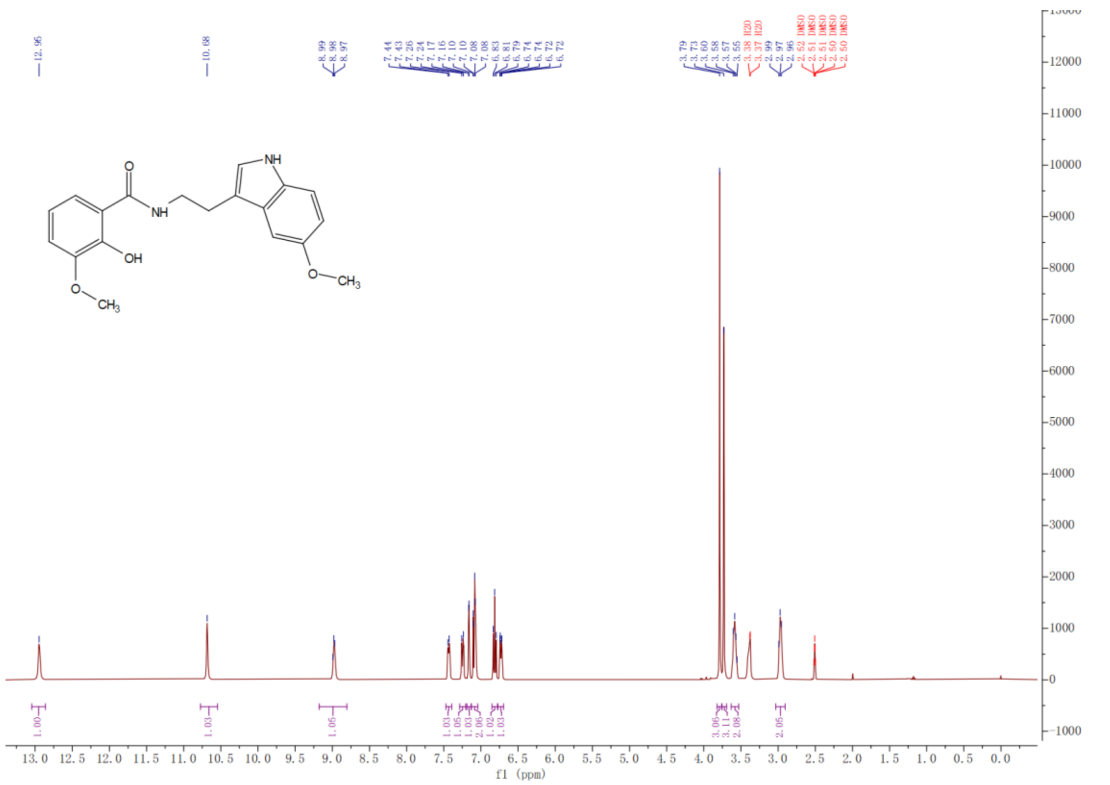

***N*-(cyclohexylmethyl)-2-hydroxybenzamide (3a)**:

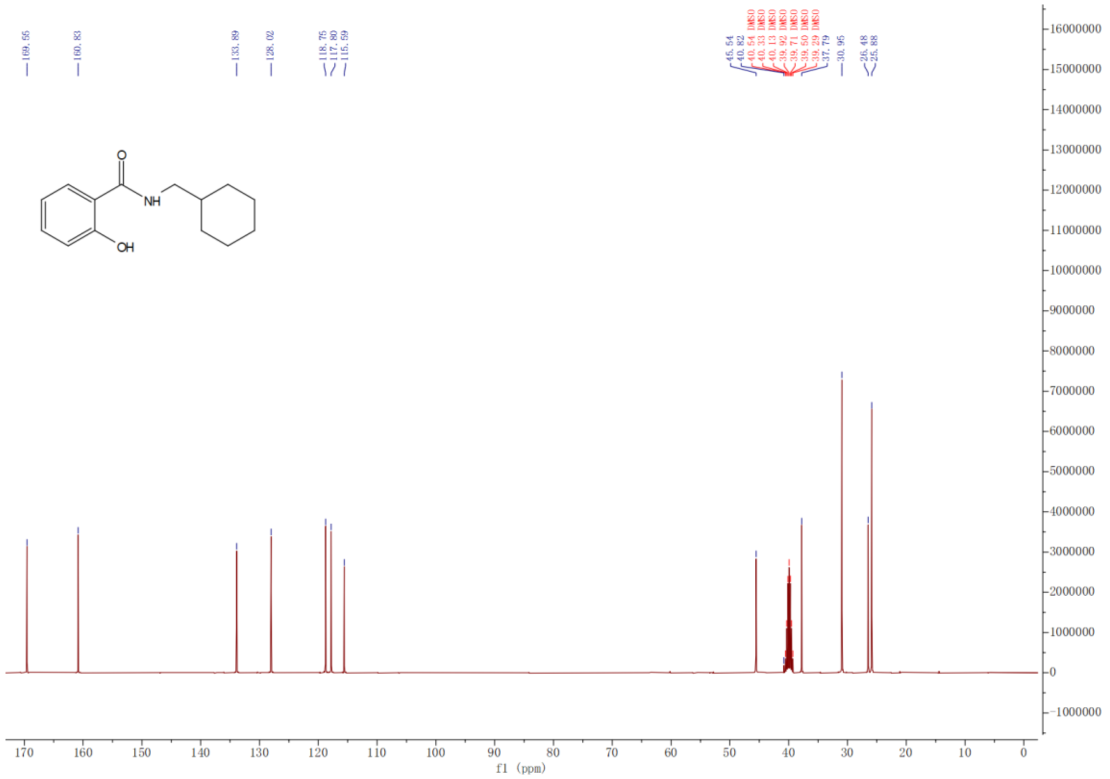

5-Chloro-*N*-(cyclohexylmethyl)-2-hydroxybenzamide (3b):

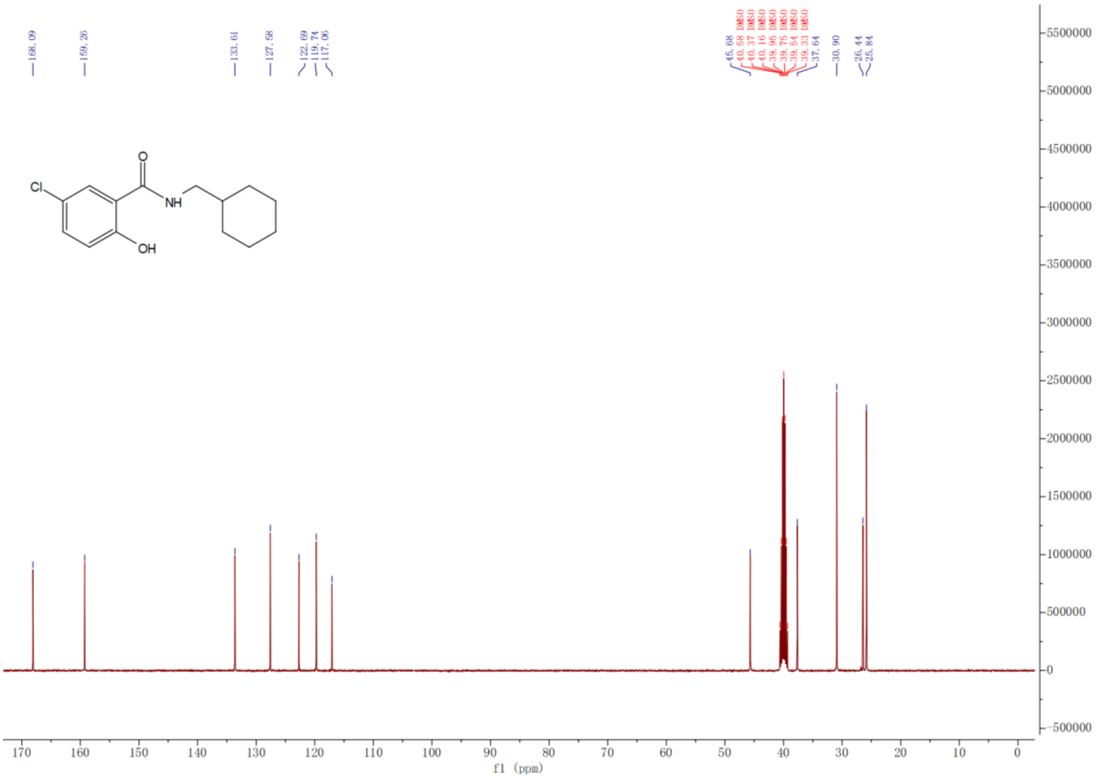

5-Chloro-*N*-cyclopentyl-2-hydroxybenzamide (3c):

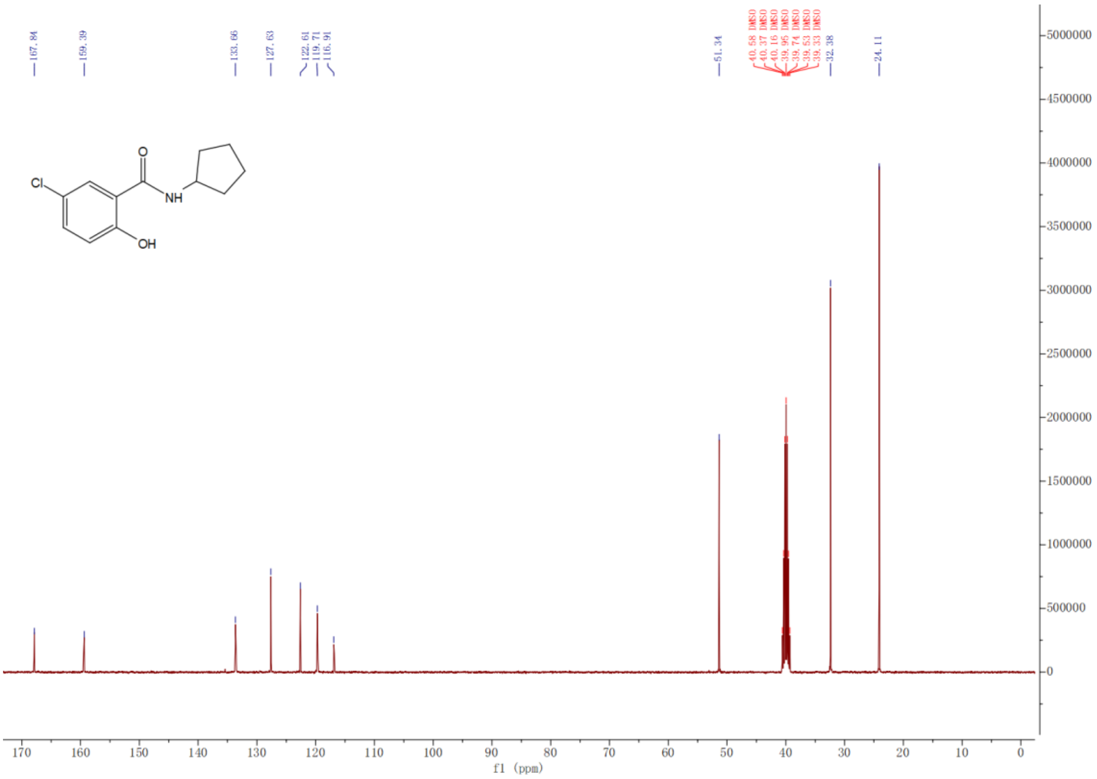

***N*-cyclopentyl-2-hydroxybenzamide (3d)**:

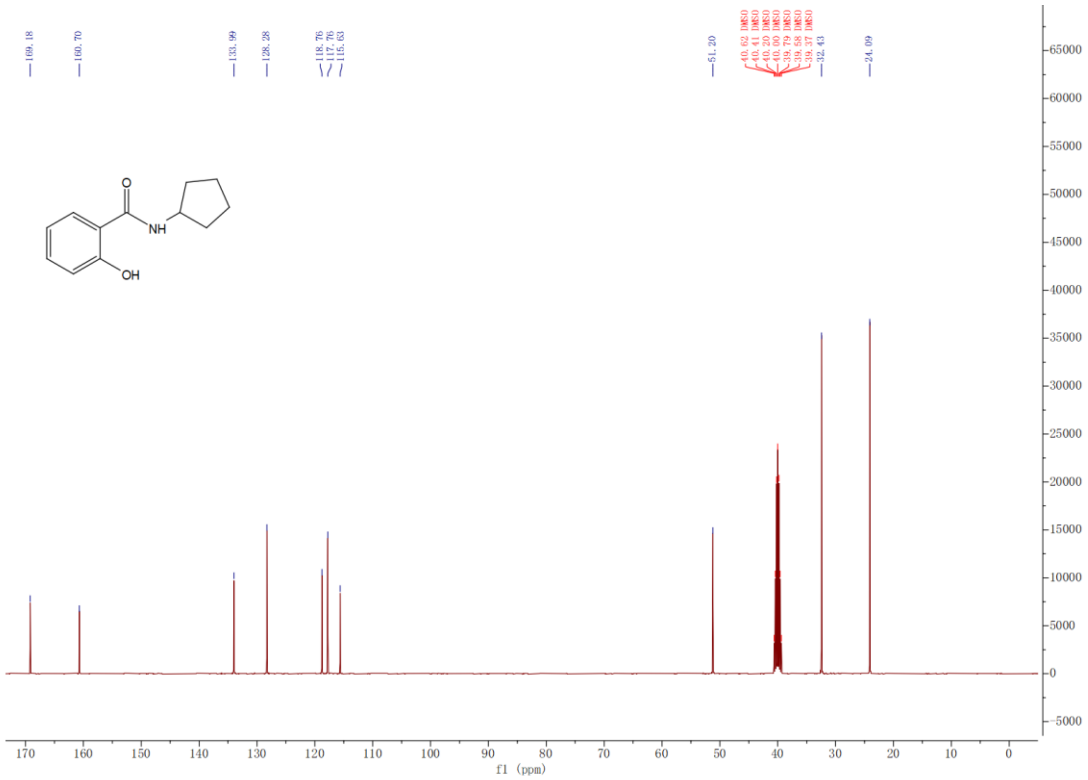

5-Chloro-*N*-cyclohexyl-2-hydroxybenzamide (3e):

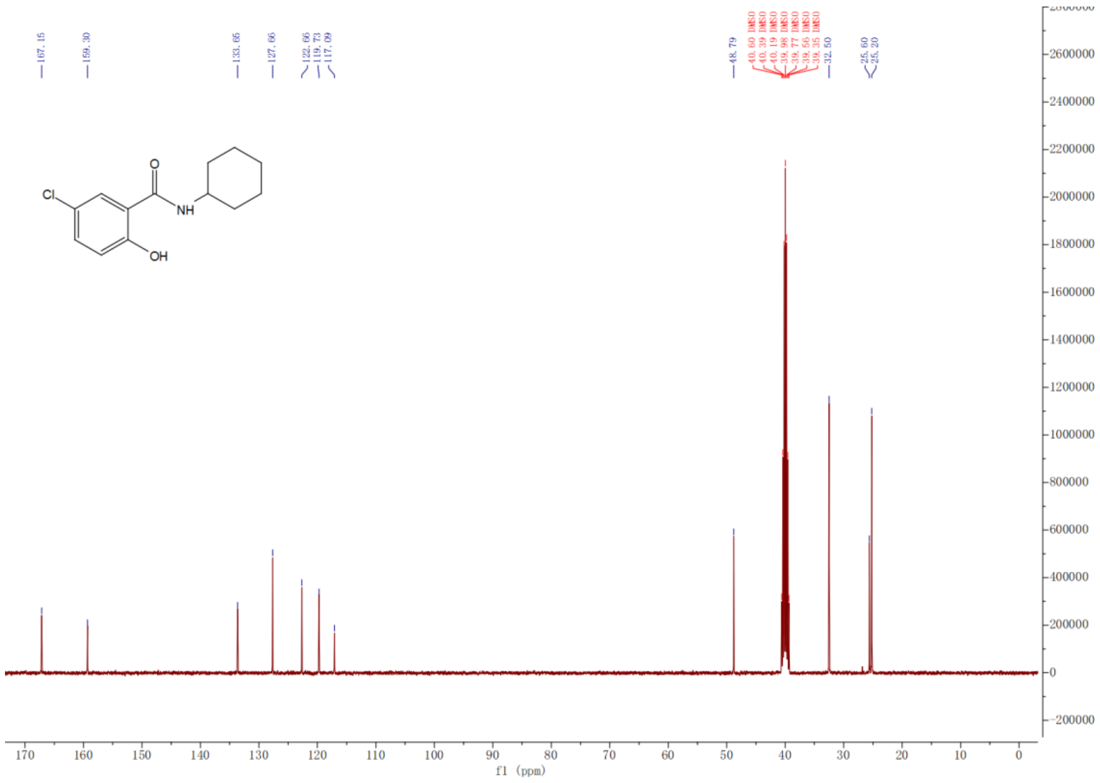

***N*-cyclohexyl-2-hydroxybenzamide (3f)**:

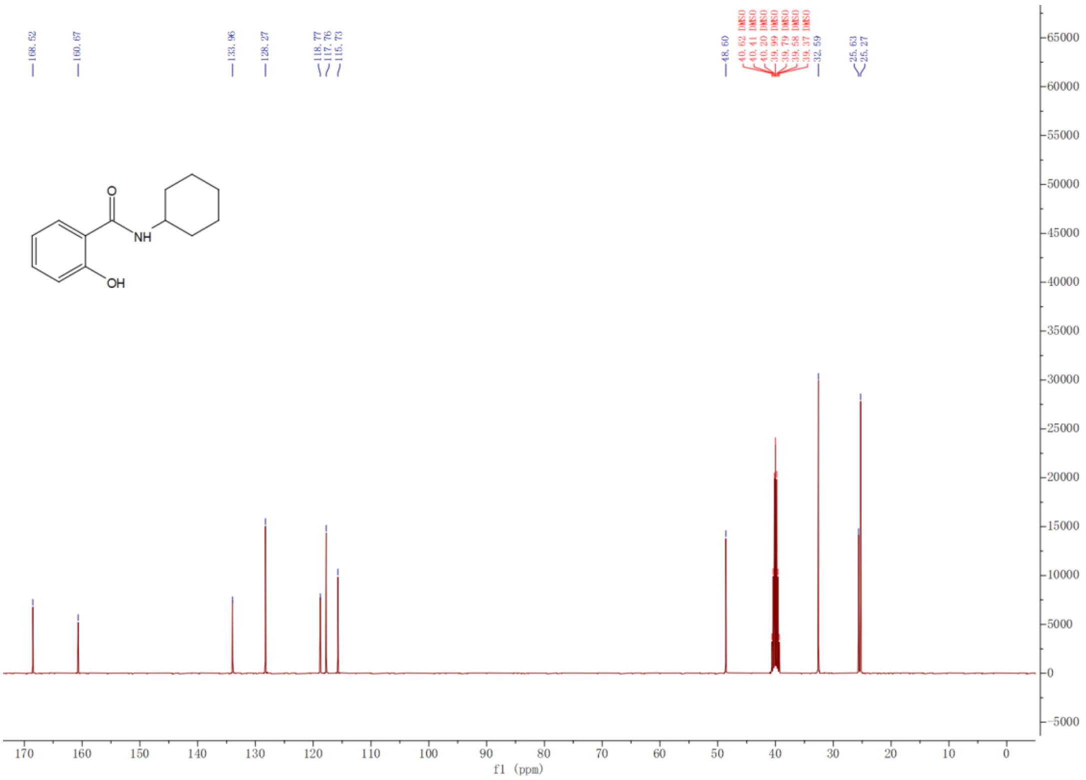

5-Chloro-2-hydroxy-*N*-(tetrahydro-2H-pyran-4-yl)benzamide (3g):

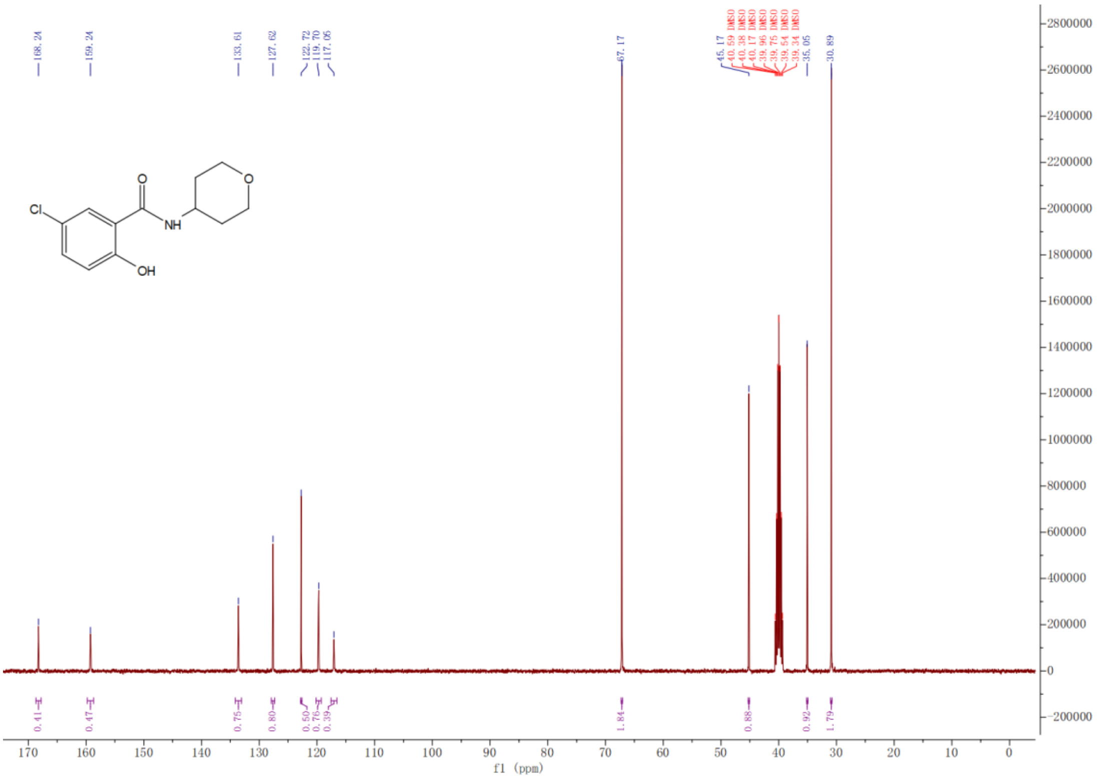

5-Chloro-*N*-hexyl-2-hydroxybenzamide (3h):

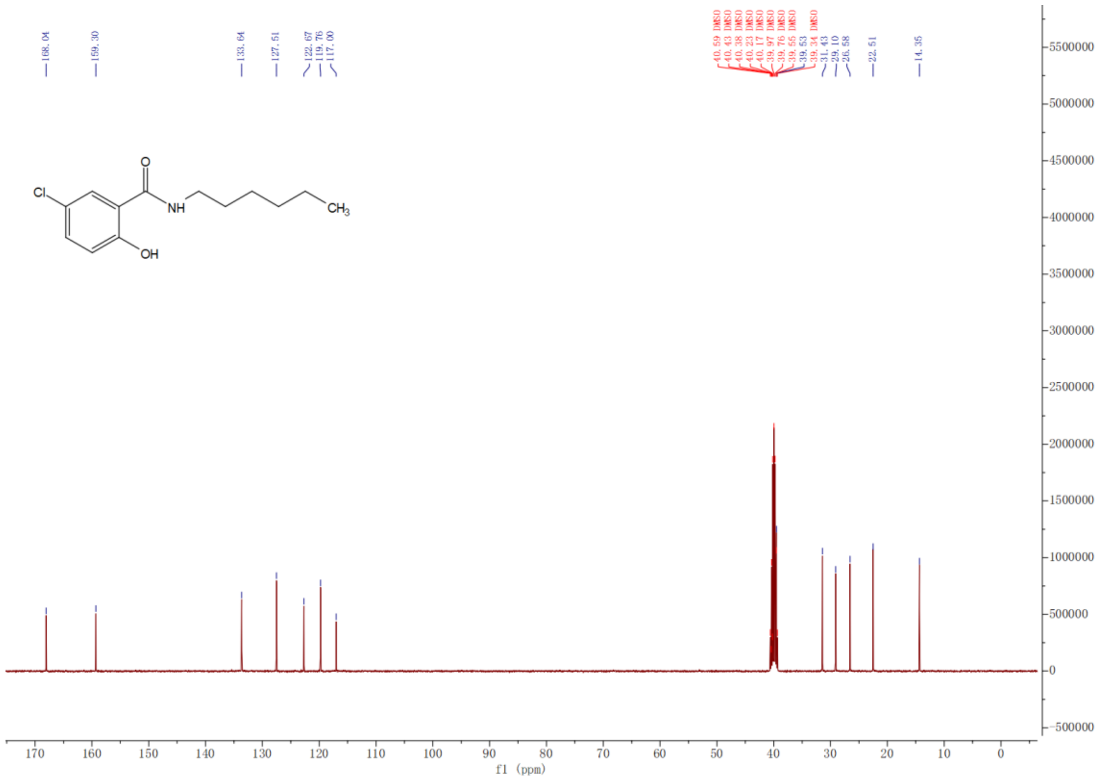

5-Bromo-*N*-hexyl-2-hydroxybenzamide (3i):

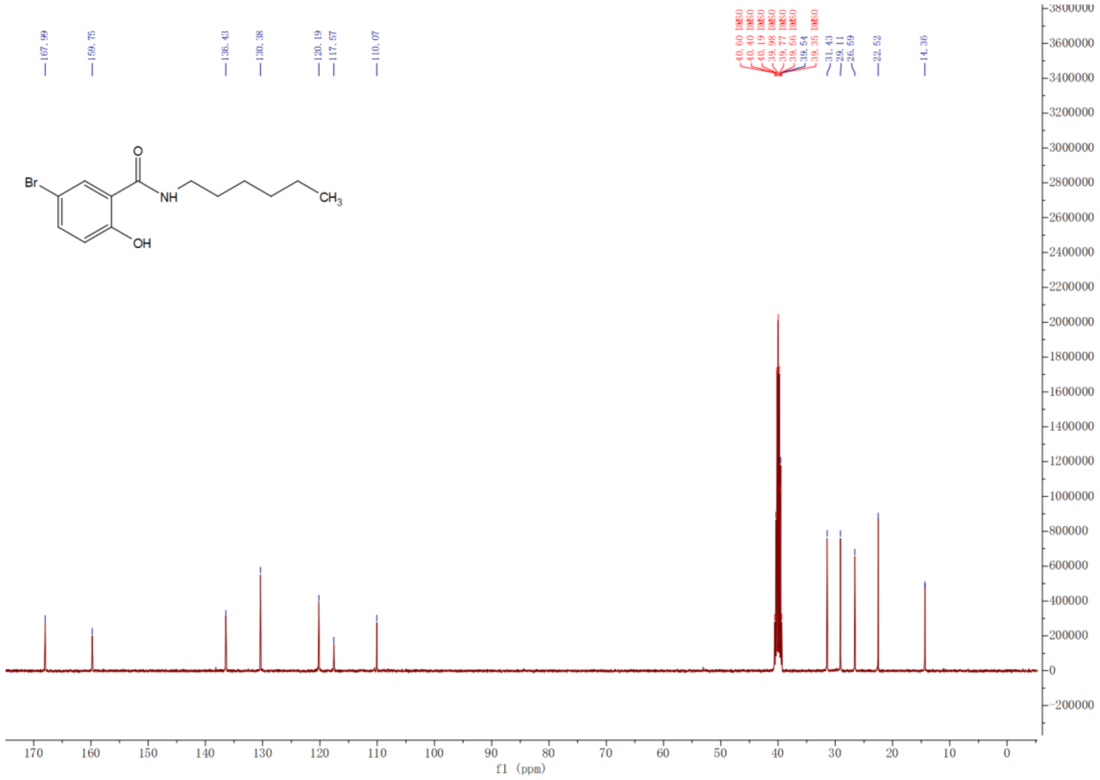

2-Hydroxy-*N*-(phenylmethyl)benzamide (4a):

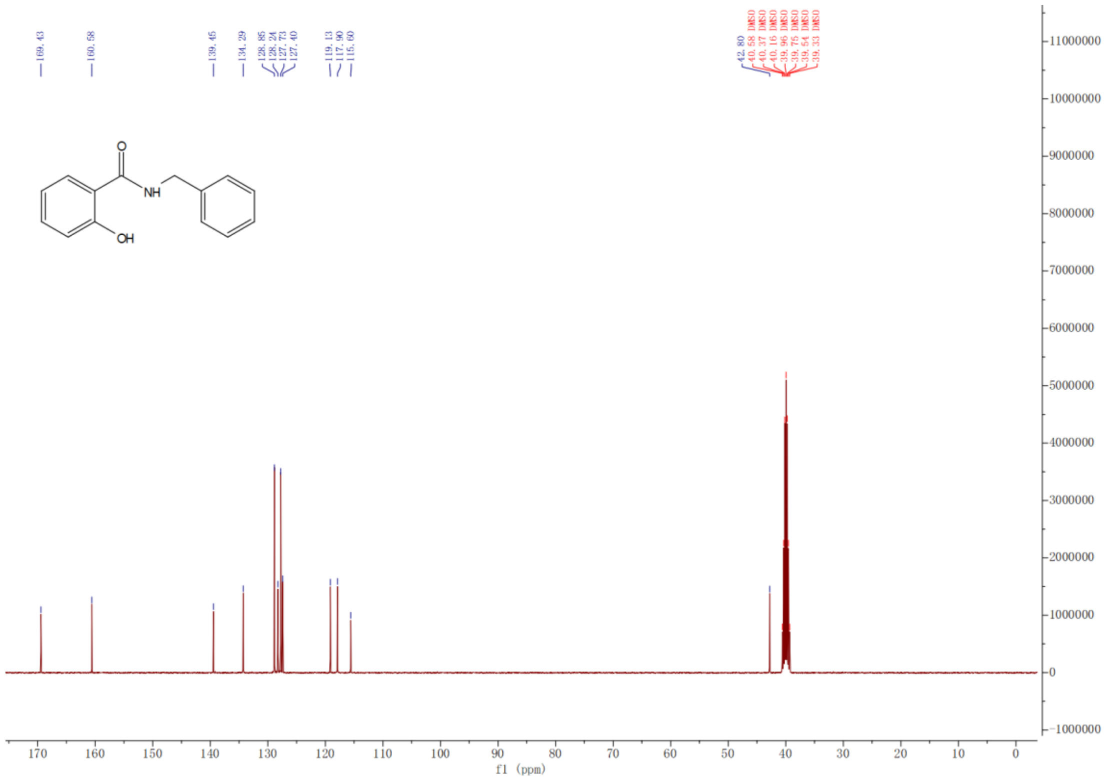

5-Chloro-*N*-[(4-chlorophenyl)methyl]-2-hydroxybenzamide (4b):

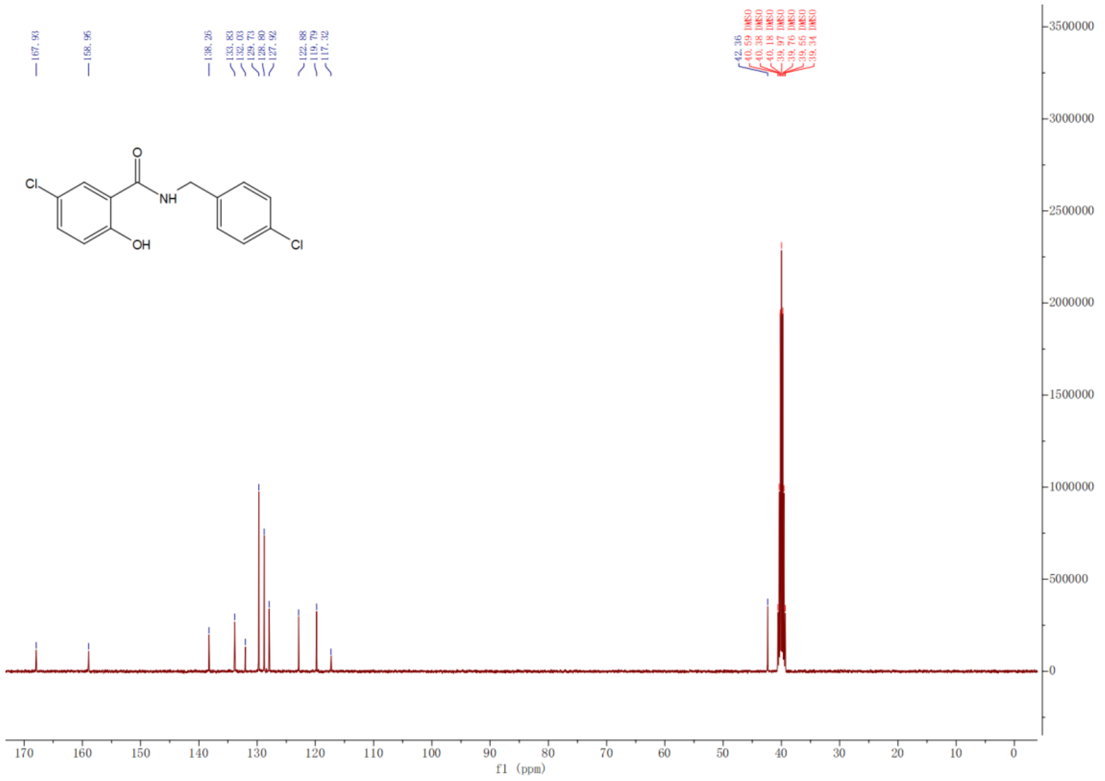

5-Chloro-*N*-[(4-methoxy)methyl]-2-hydroxybenzamide (4c):

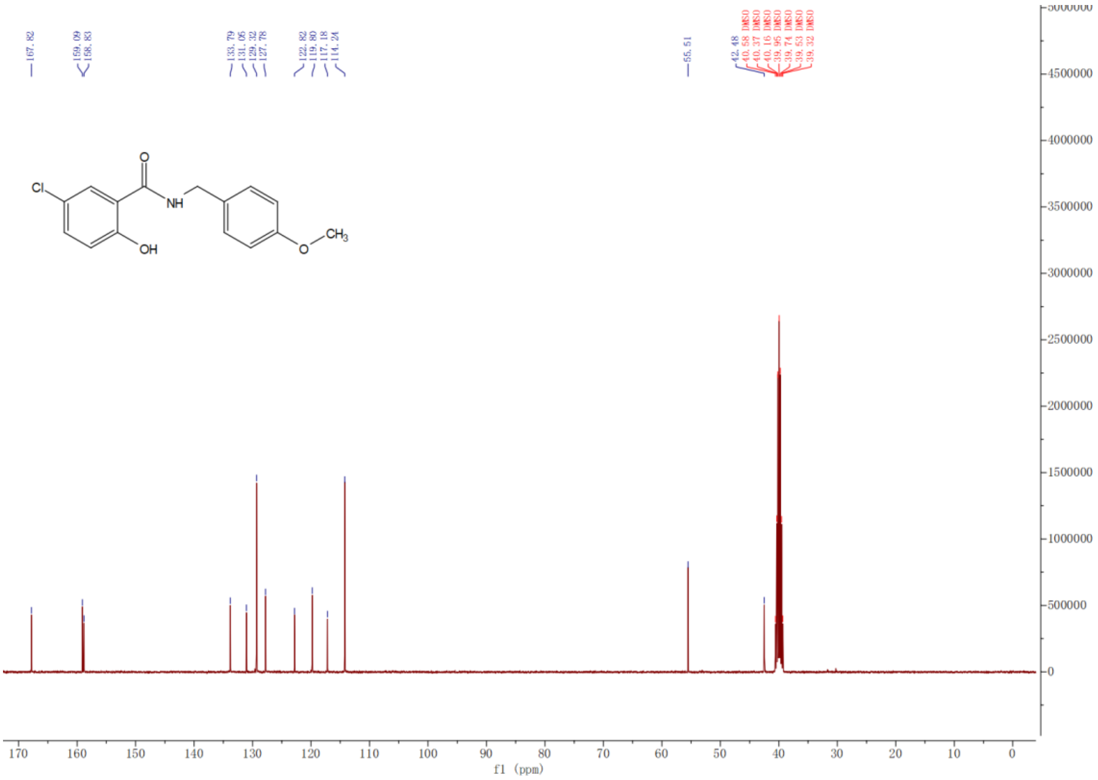

5-Chloro-2-hydroxy-*N*-(2-phenylethyl)benzamide (4d):

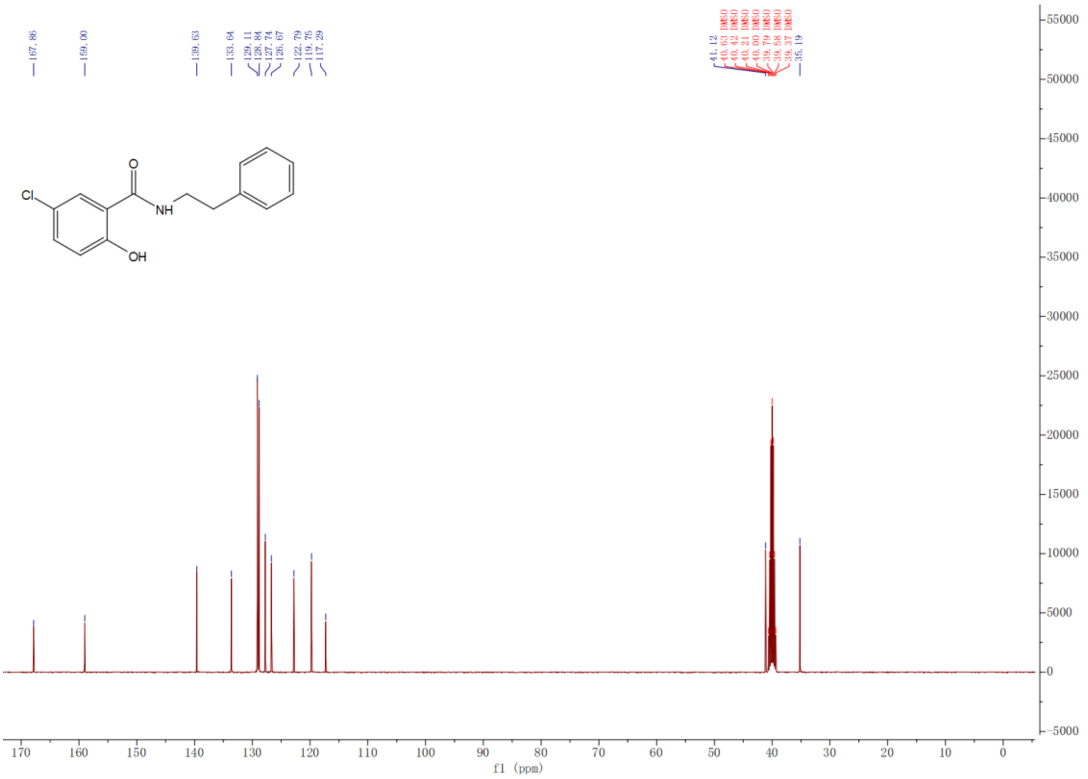

5-Chloro-N-(benzyl)-2-hydroxybenzamide(4e):

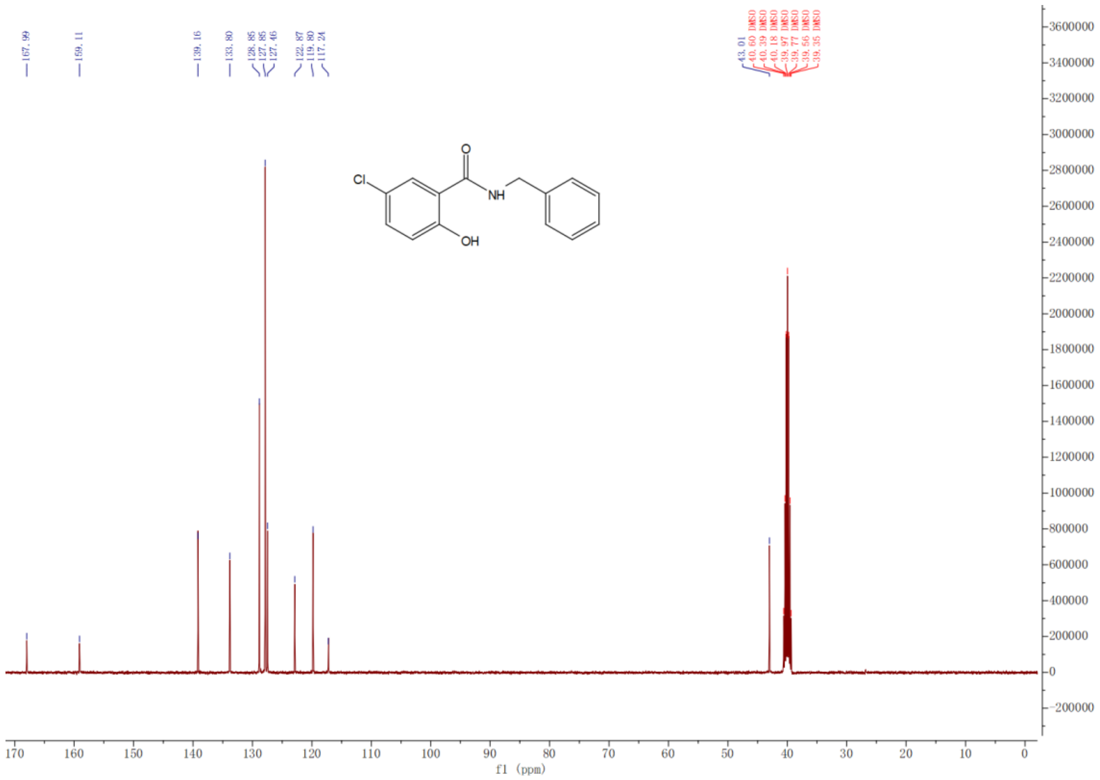

N-(2-(1H-indol-3-yl)ethyl)-2-hydroxybenzamide (5a):

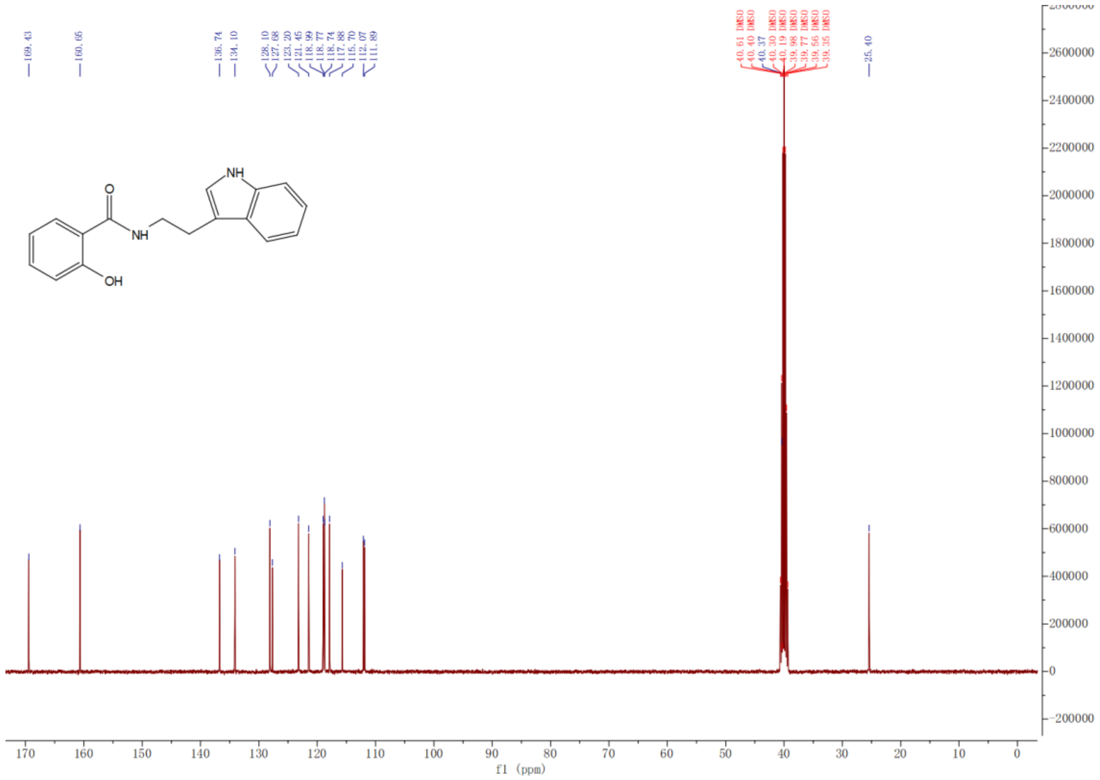

***N*-(2-(1H-indol-3-yl)ethyl)-5-bromo-2-hydroxybenzamide(5b)**:

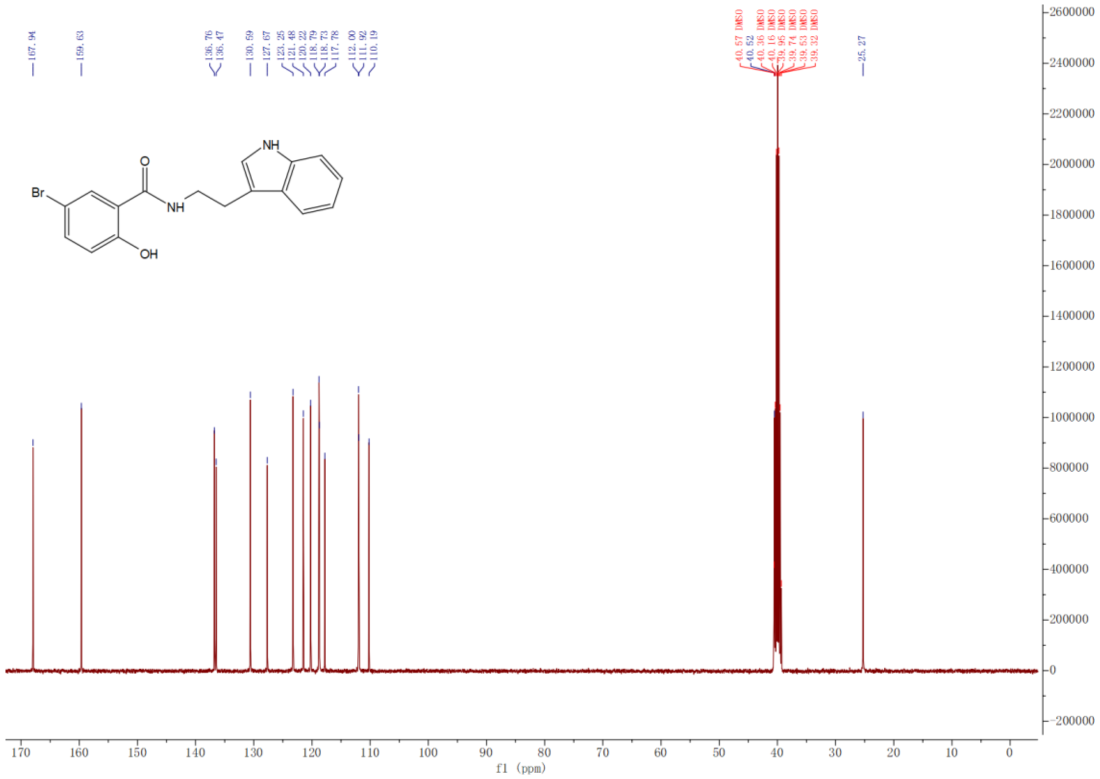

_N_-(2-(1H-indol-3-yl)ethyl)-2-hydroxy-4-methylbenzamide (5c):

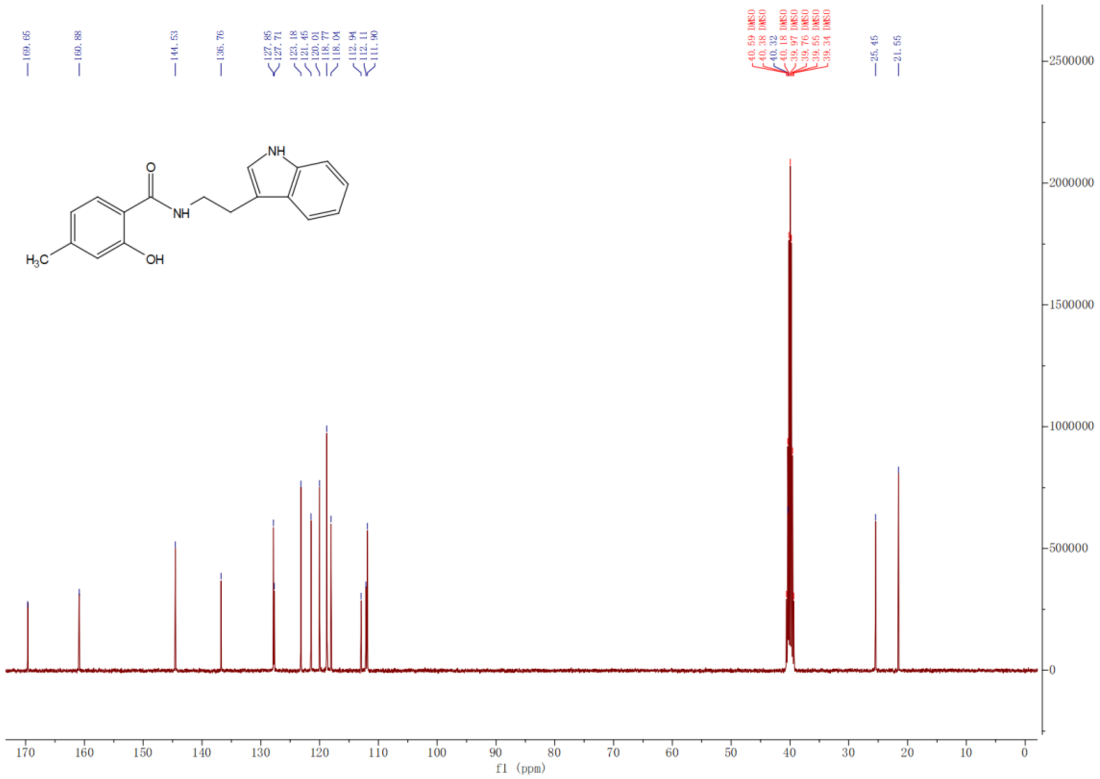

2-hydroxy-_N_-(2-(5-methoxy-1H-indol-3-yl)ethyl)benzamide (5d):

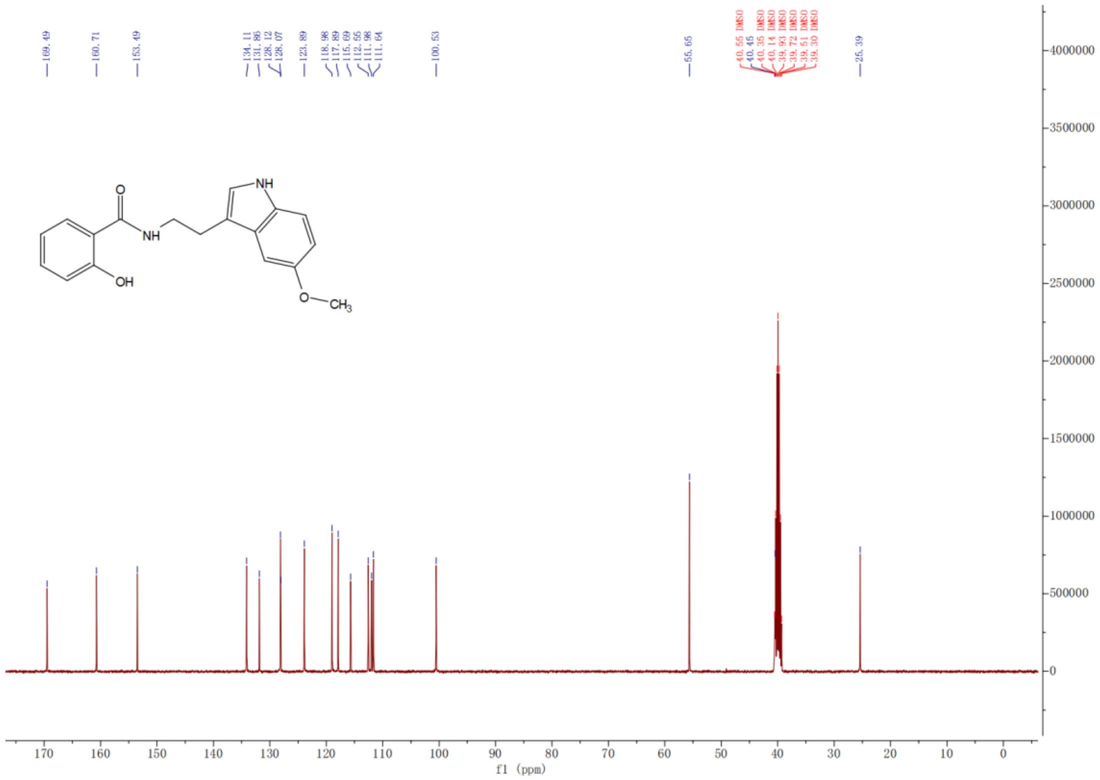

***N*-(2-(5-methoxy-1H-indol-3-yl)ethyl)-2-hydroxy-3-methylbenzamide(5e)**:

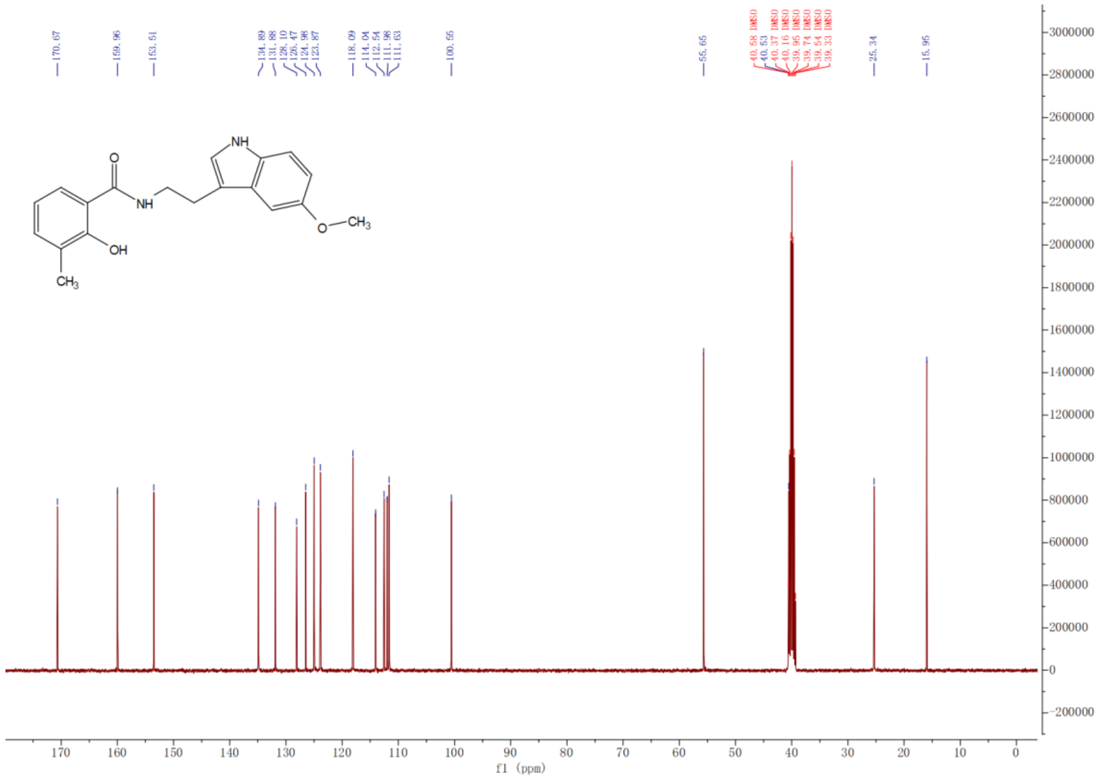

***N*-(2-(5-chloro-1H-indol-3-yl)ethyl)-2-hydroxy-4-methylbenzamide (5f)**:

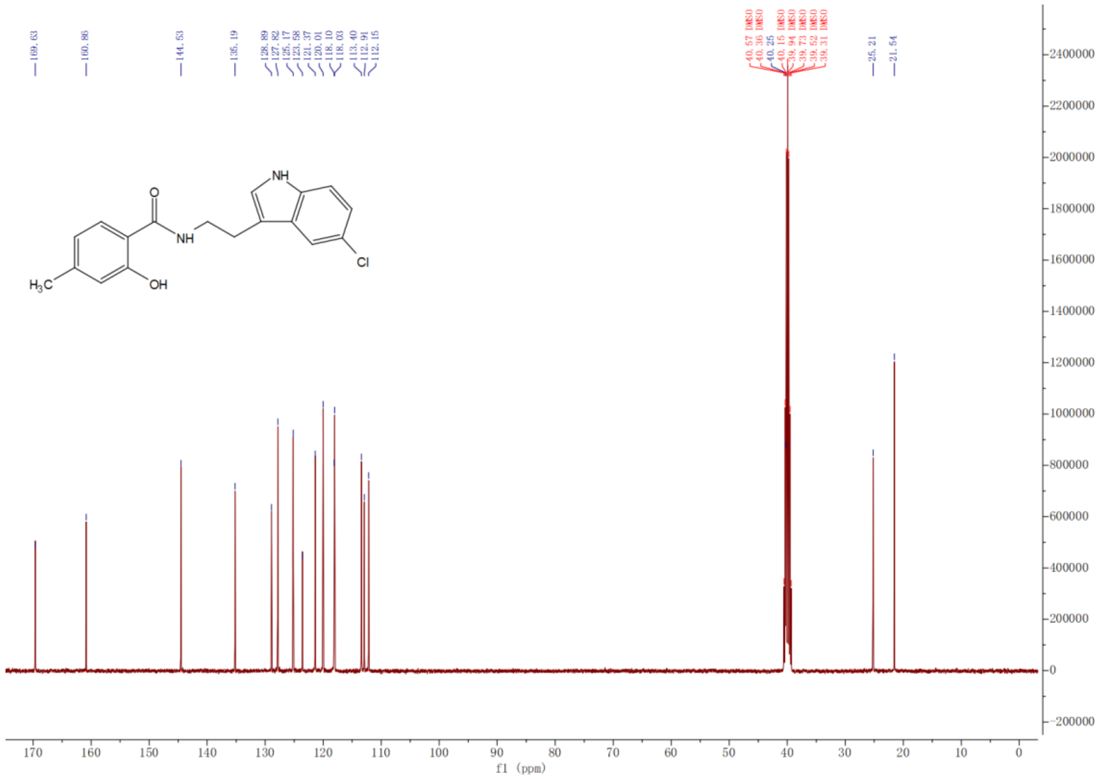

***N*-(2-(5-chloro-1H-indol-3-yl)ethyl)-2-hydroxybenzamide (5g):**

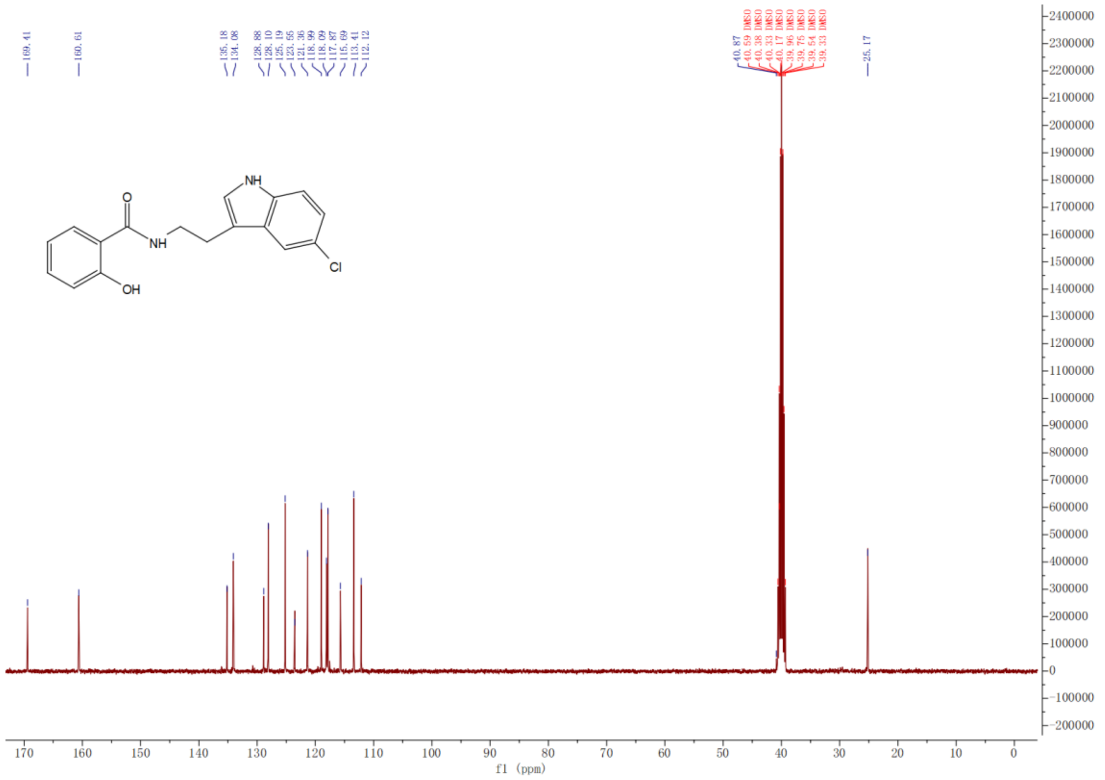

***N*-(2-(1H-indol-3-yl)ethyl)-2-hydroxy-5-methylbenzamide (5h):**

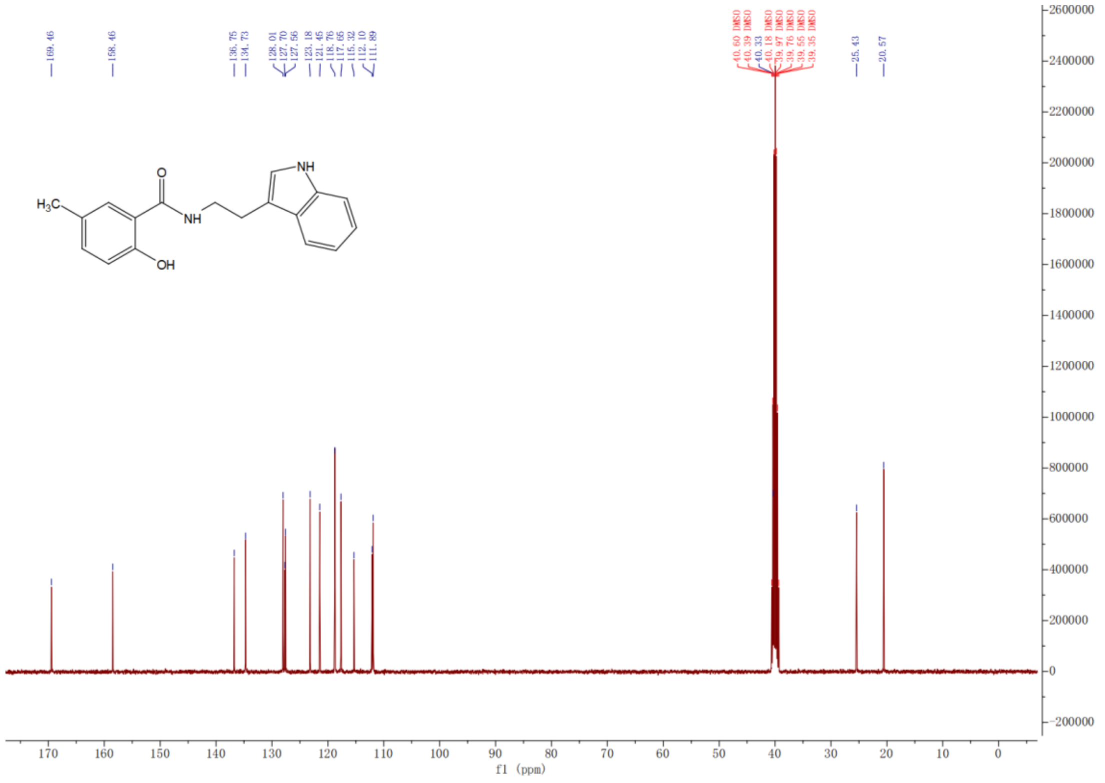

N-(2-(1H-indol-3-yl)ethyl)-5-chloro-2-hydroxybenzamide (5i):

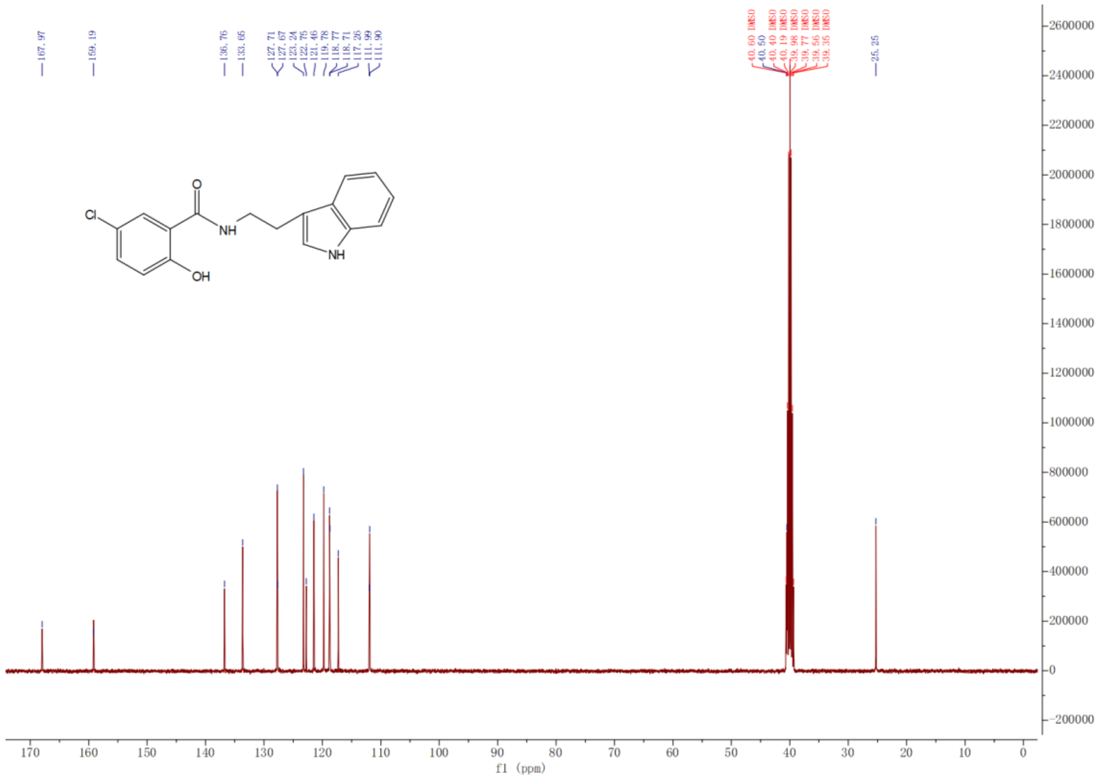

***N*-(2-(5-methoxy-1H-indol-3-yl)ethyl)-5-chloro-2-hydroxybenzamide (5j):**

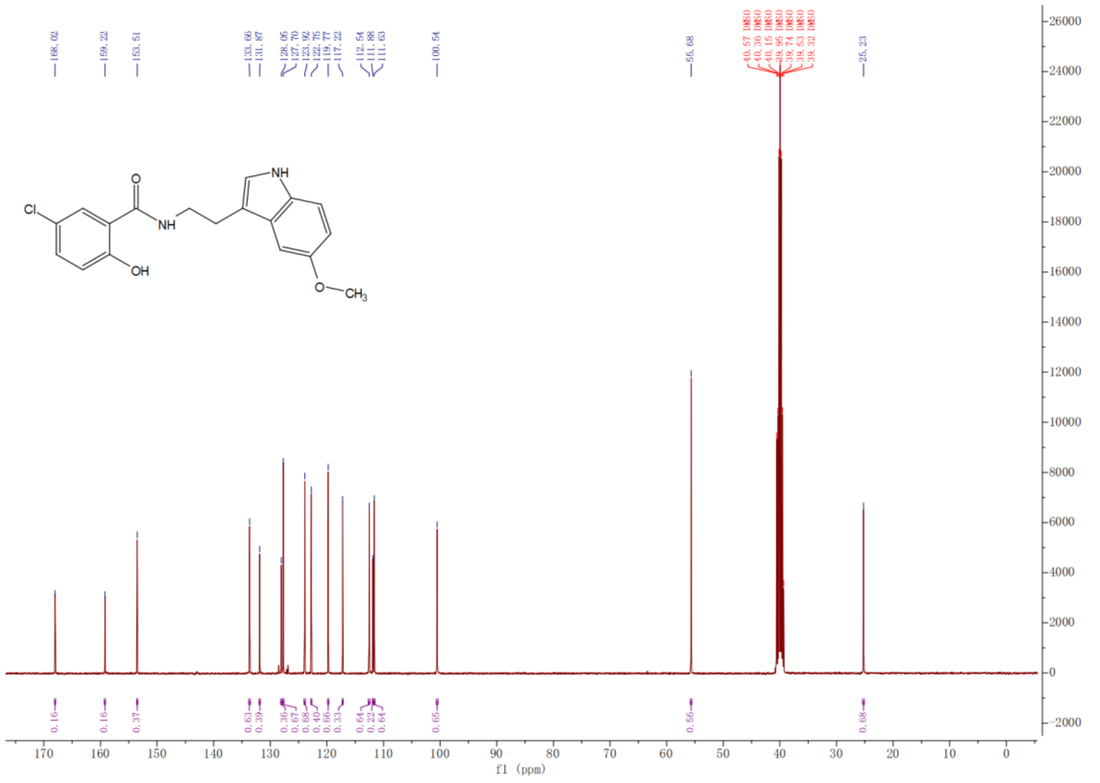

***N*-(2-(5-methoxy-1H-indol-3-yl)ethyl)-5-bromo-2-hydroxybenzamide (5k):**

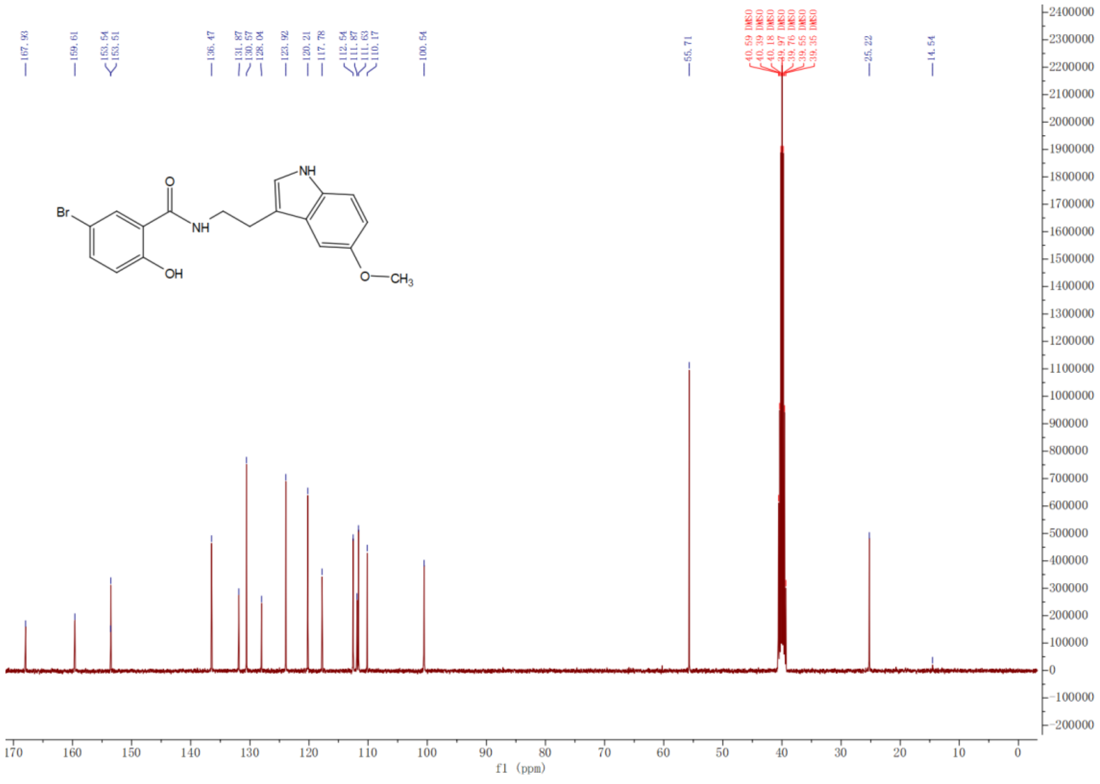

***N*-(2-(5-chloro-1H-indol-3-yl)ethyl)-5-bromo-2-hydroxybenzamide (5l):**

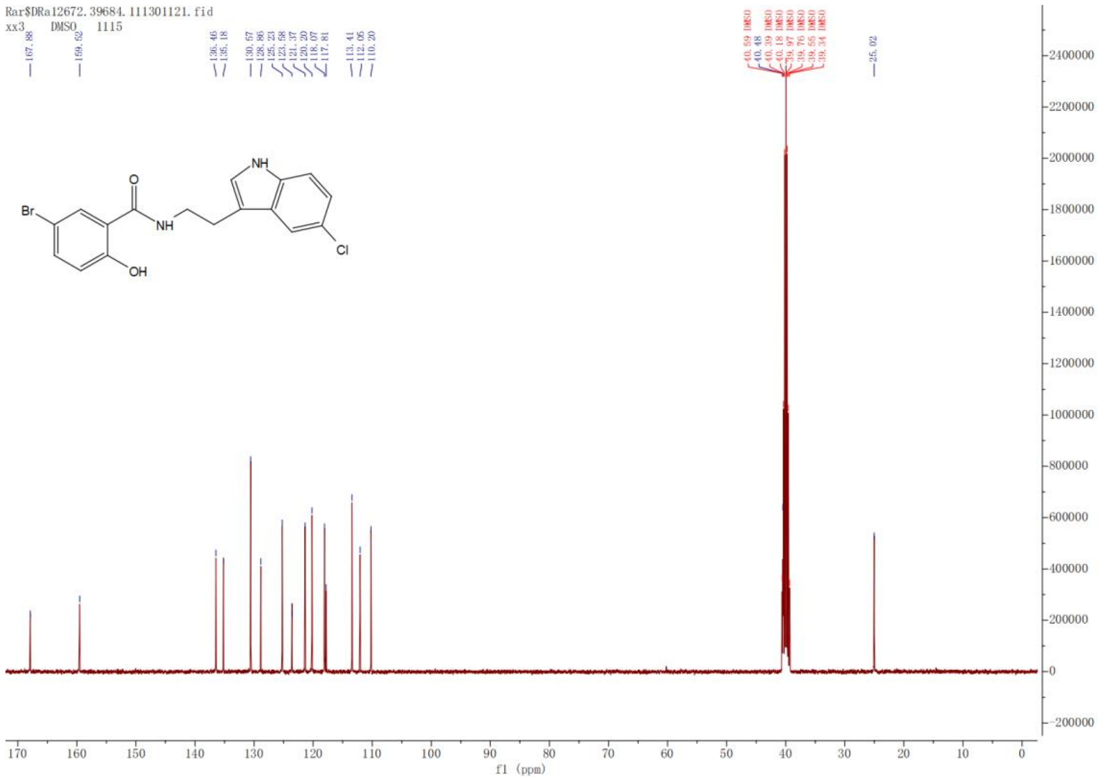

N-[2-(5-Chloro-1H-indol-3-yl)-ethyl]-2-hydroxy-3-methyl benzamide (5m).

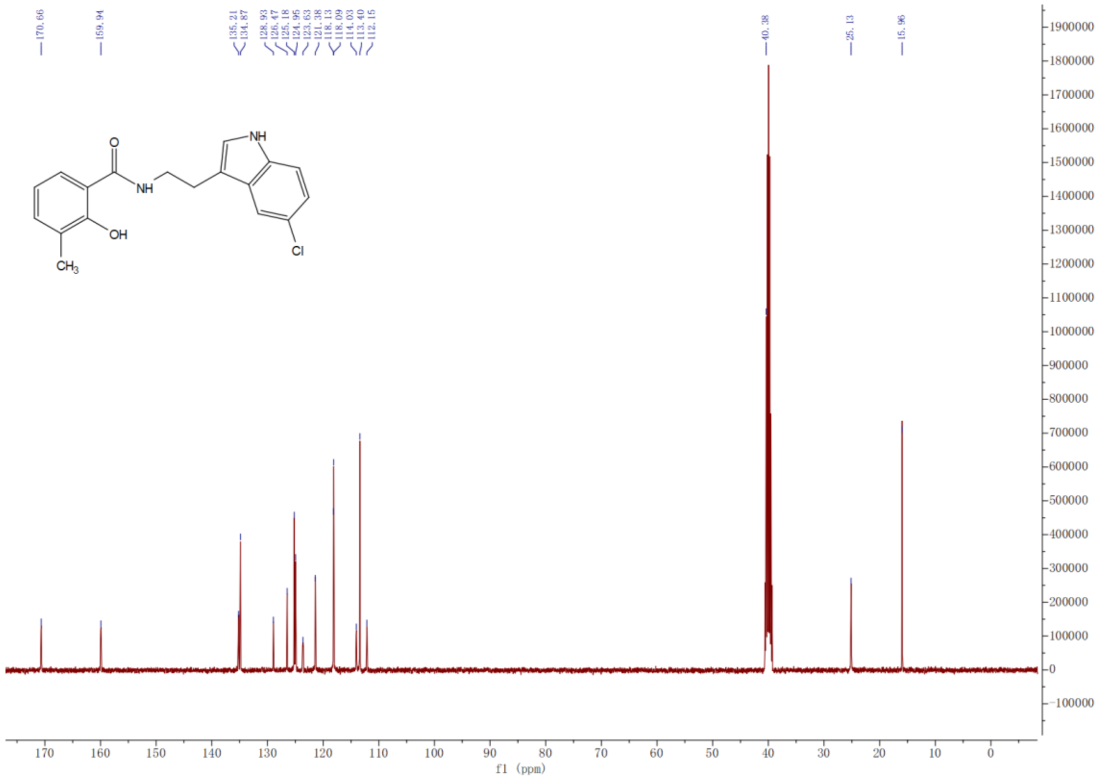

***N*-(2-(5-chloro-1H-indol-3-yl)ethyl)-2-hydroxy-5-methylbenzamide (5n):**

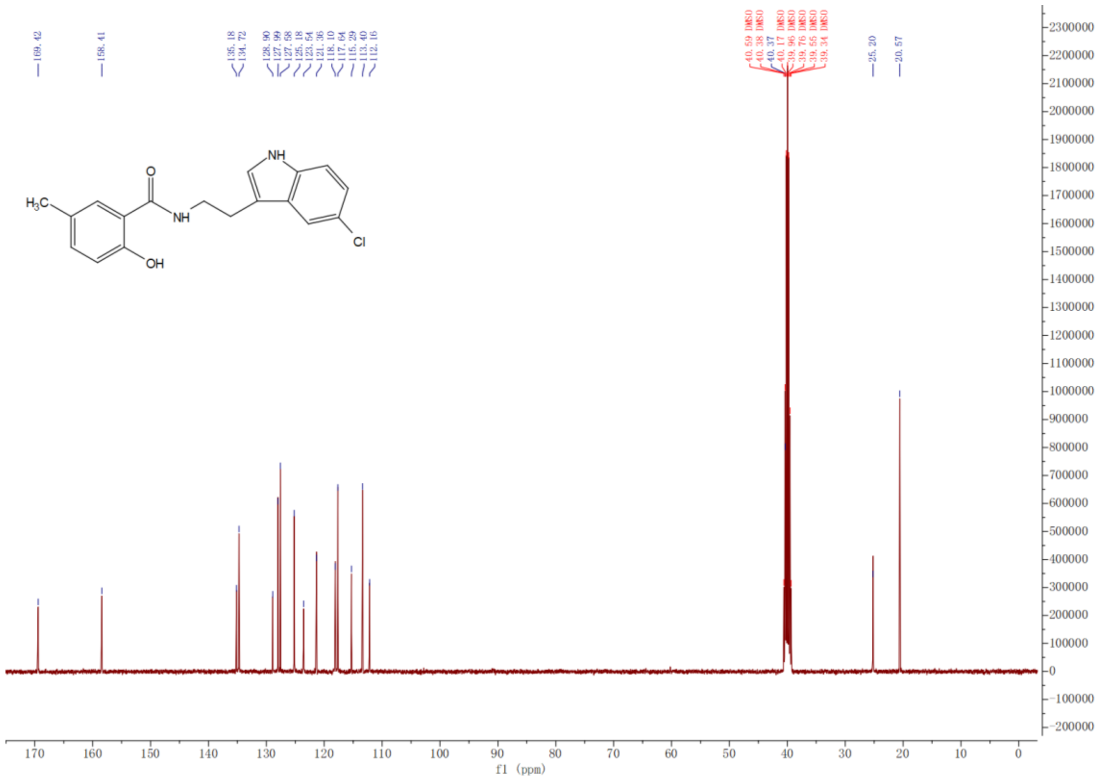

N-(2-(1H-indol-3-yl)ethyl)-2-hydroxy-5-methoxybenzamide (5o):

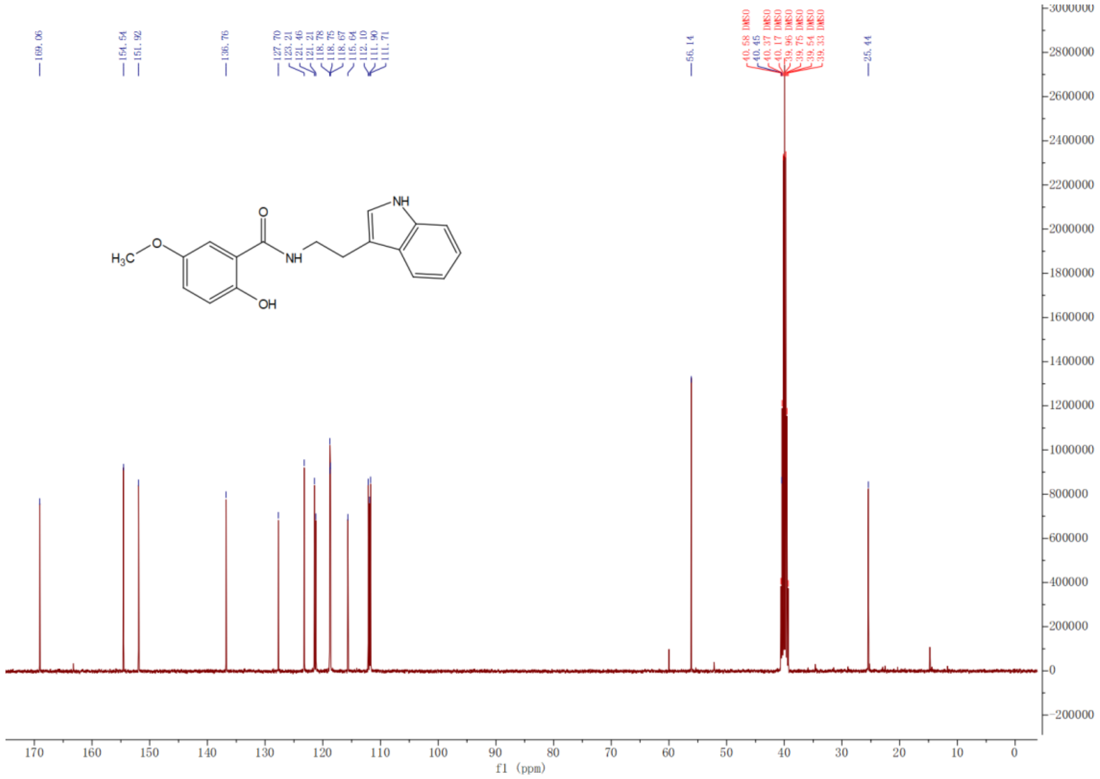

N-(2-(5-methoxy-1H-indol-3-yl)ethyl)-2-hydroxy-5-methoxybenzamide (5p):

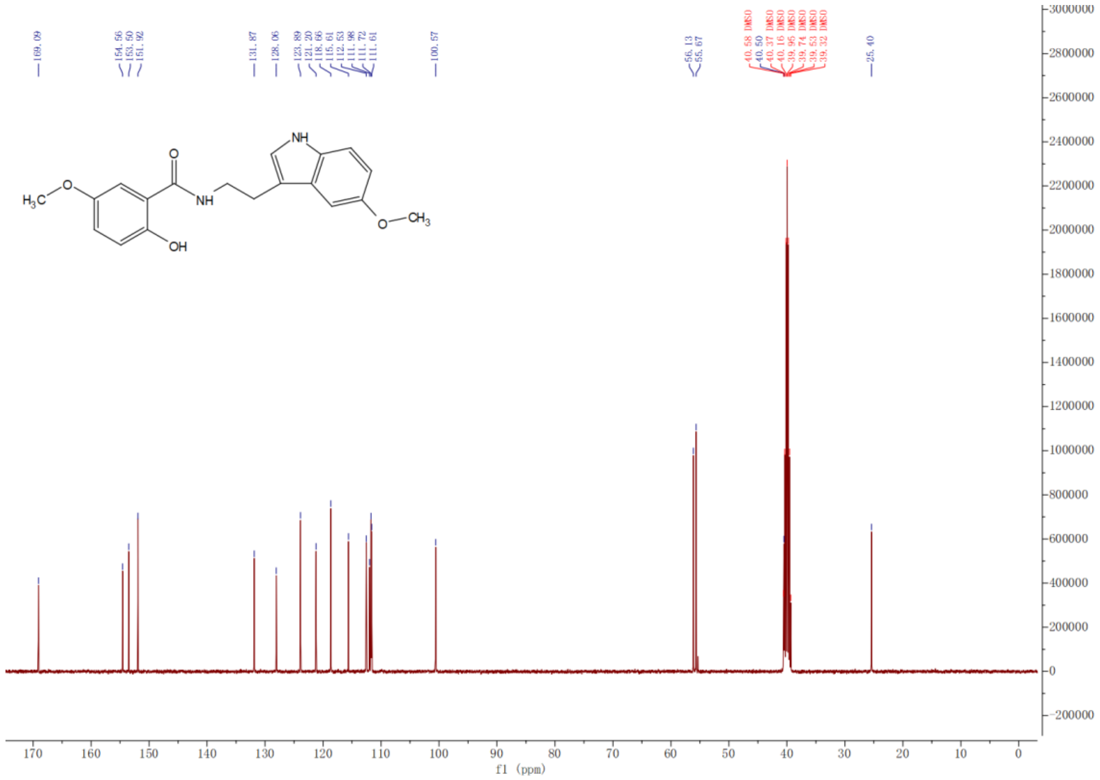

N-(2-(5-chloro-1H-indol-3-yl)ethyl)-2-hydroxy-5-methoxybenzamide (5q):

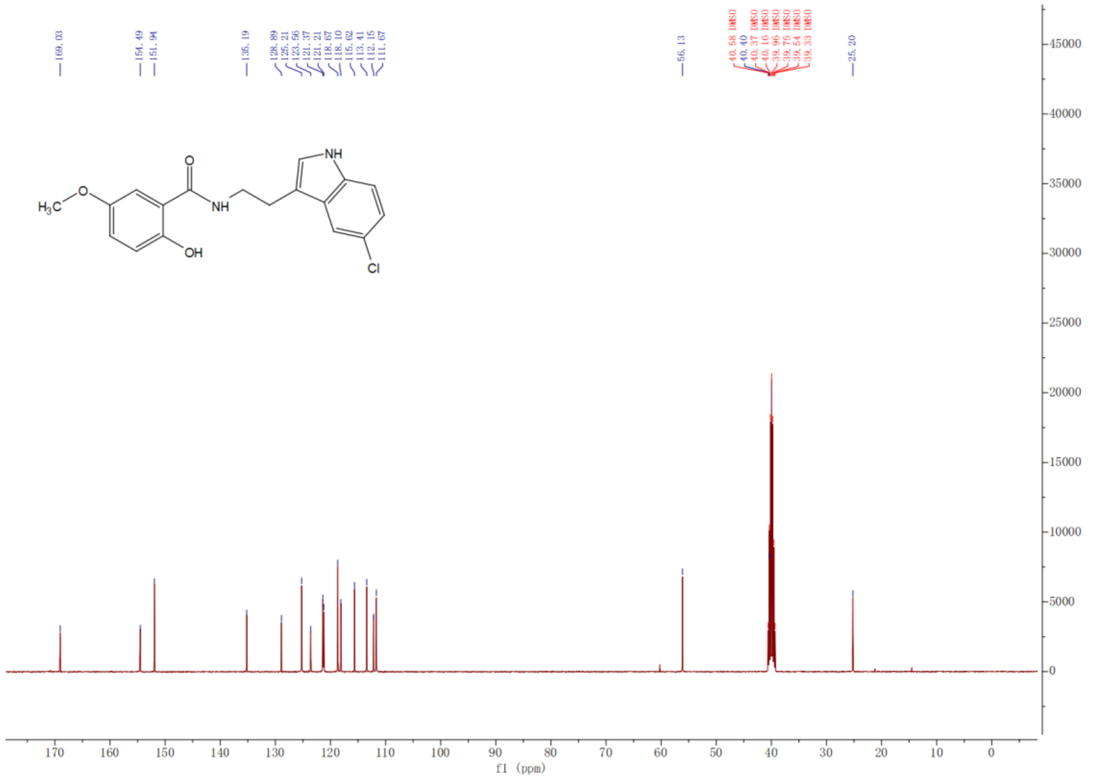

N-(2-(5-methoxy-1H-indol-3-yl)ethyl)-2-hydroxy-4-methylbenzamide (5r):

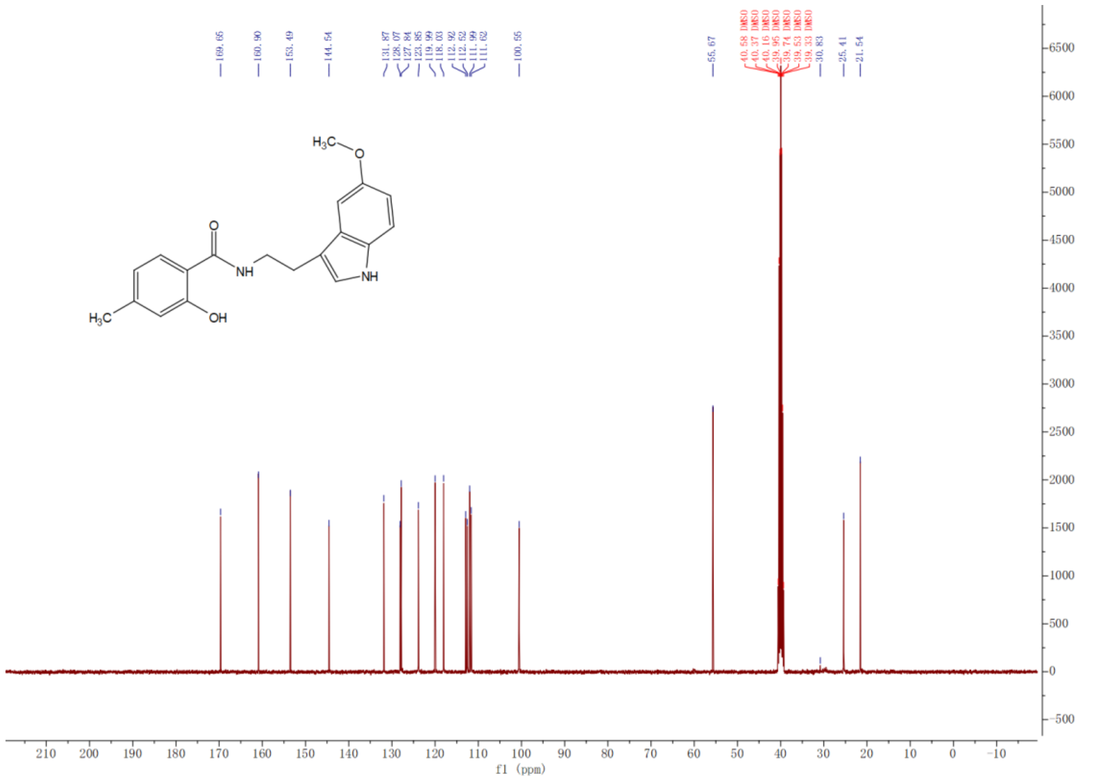

N-(2-(1H-indol-3-yl)ethyl)-2-hydroxy-3-methylbenzamide (5s):

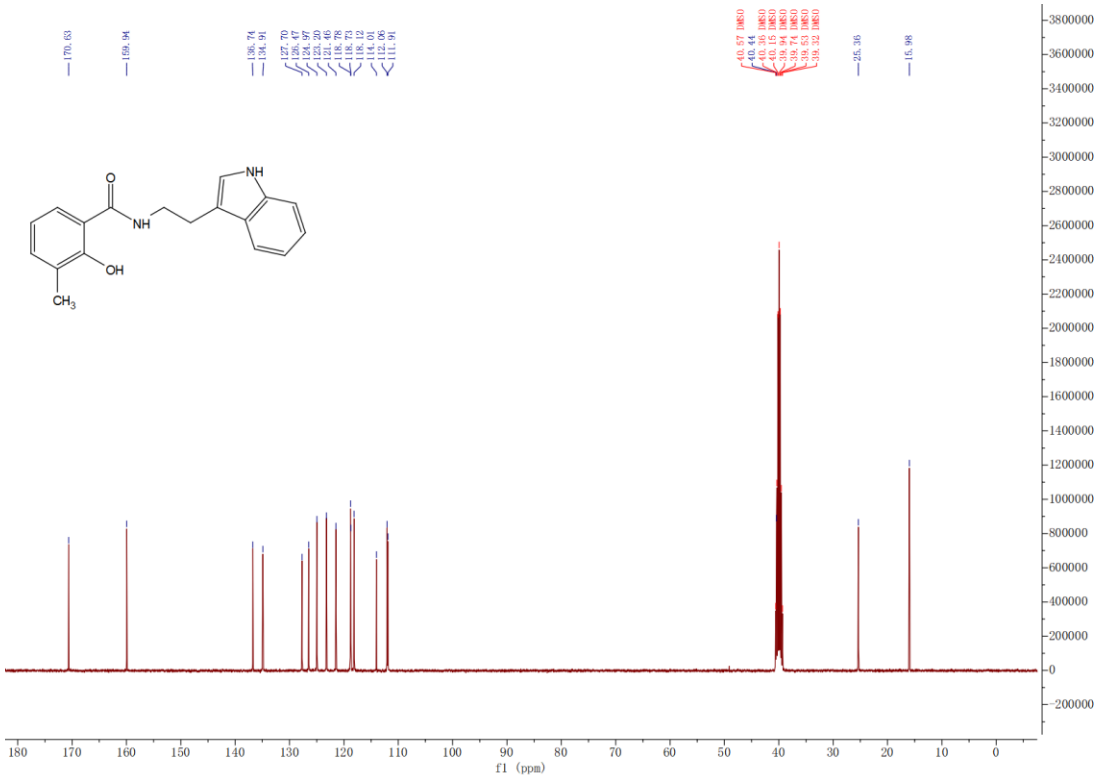

N-(2-(5-chloro-1H-indol-3-yl)ethyl)-2-hydroxy-3-methylbenzamide (5t):

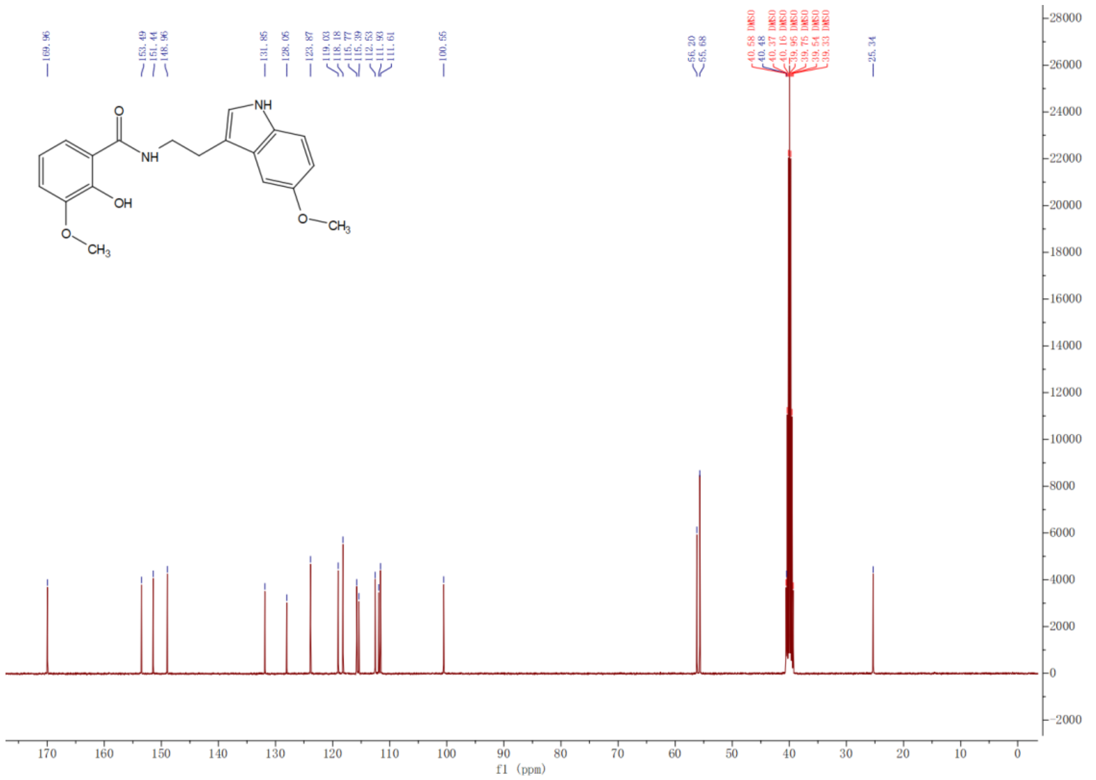

Notes and references

1. N. J. Roy, S. N. Save, V. K. Sharma, B. Abraham, A. Kuttanamkuzhi, S. Sharma, M.Lahiri and P. Talukdar, *Chem. Eur. J.*, 2023, **29**, e202301412.

2. J. M. Xu, J. B. Cabrera, M. C. Ledesma, H. Y. Chen, Y. Xue, E. A. Wold, J. Pachón, J. Zhou and J. S. Céspedes, *Int. J. Mol. Sci.*, 2021, **22**, 1617.

3. K. Madasamy, M. H. Balakrishnan, R. Korivi and S. Mannathan, *J. Org. Chem.*, 2022, **87**, 8752-8756.

4. A.Y. Yu and X. C. Wang, *J. Mol. Struct.*, 2024, **1295**, 136624.

5. J. M. Xu, J. B. Cabrera, H. Y. Chen, J. Pachón, J. Zhou and J. S. Céspedes, *J. Med. Chem.*, 2020, **63**, 3142-3160.

6. M. E. Mitxeltorena, H. F. Romero, S. R. Inza, E. Masiá, M. Nenchova, J. Montesinos, L. M. Gonzalez, G. Porras, M. Orzáez, M. J. Vicent, C. Gil, E. A. Gomez, A. G. Saez and A. Martinez, *J. Med. Chem.*, 2025, **68**, 1179-1194.

7. J. H. Su, G. P. Wu, X. Peng, G. Q. Zhao, Y. Peng, H. H. Zhang, Y. T. Zhao, R. Sun, S. T. Chen, Y. Tian and Z. Wang, *ACS Chem. Neurosci.*, 2023, **14**, 2146-2158.

8. G. P. Wu, Z. C. Hu, B. Li, X. Z. Wei, , L. S. Zhuo, Y. X. Chen, Y. Tian, Y. T. Zhao, Z. Wang, Y. Peng and X. Peng, *Eur. J. Med. Chem.*, 2024, **278**, 116795.

9. X. H. Fan, J. F. Li, X. M. Deng, Y. M. Lu, Y. Y. Feng, S. M. Ma, H. X. Wen, Q. Y. Zhao, W. Tan, T. Shi and Z. Wang, *Eur. J. Med. Chem.*, 2020, **193**, 112217.

10. Y. L. Bai, D. Liu, H. H. Zhang, Y. Y. Wang, D. G. Wang, H. B. Cai, H. X. Wen, G. Q. Yuan, H, An, Y. L. Wang, T. Shi and Z. Wang, *Bioorg. Chem.*, 2021, **115**, 105255.

11. R. D. Xiong, D. X. He, X. P. Deng, J. Liu, X. Y. Lei, Z. Z. Xie, X. Cao, Y. M. Chen, J. M.

Peng and G. T. Tang, *Med. Chem. Commun.*, 2019, **10**, 573-583.
12. Molho and Darius, FR 1207956, 1960-02-19.
